# Excited-state orbital angular momentum enables all-optical molecular spin coherence

**DOI:** 10.1039/d6sc04497b

**Published:** 2026-06-26

**Authors:** Erica Sutcliffe, Jonathan P. Aalto, Ryan G. Hadt

**Affiliations:** a Division of Chemistry and Chemical Engineering, Arthur Amos Noyes Laboratory of Chemical Physics, California Institute of Technology Pasadena California 91125 USA rghadt@caltech.edu

## Abstract

Paramagnetic molecules are promising quantum sensors with dimensions and environmental compatibility inaccessible to solid-state defects. Realizing this promise, however, requires optical methods for initializing and reading out coherent spin dynamics. Ultrafast pump–probe polarization spectroscopy provides such a route, but previous demonstrations have relied on high-symmetry complexes in which ground-state orbital angular momentum enables spin–photon coupling. Here we demonstrate the first ultrafast spin coherence measurements on non-octahedral molecules and show that excited-state orbital angular momentum can instead provide the optical interface in axial tungsten(v)–oxo complexes. Circularly polarized excitation generates room-temperature spin coherence that persists for several nanoseconds, enabling time-domain optical detection of electron paramagnetic resonance (EPR) spectra, including *g* value anisotropy in a polymer matrix, and solution-phase DC magnetic field detection down to 5 mT. This work establishes a route to ultrafast, all-optical spin spectroscopy and quantum sensing in lower-symmetry, chemically tunable coordination complexes.

## Abstract 2

We demonstrate the first ultrafast spin coherence measurements on non-octahedral molecules. Coherence persists for several nanoseconds at room temperature, enabling time-domain optical detection of electron paramagnetic resonance (EPR) spectra.

## Graphical abstract

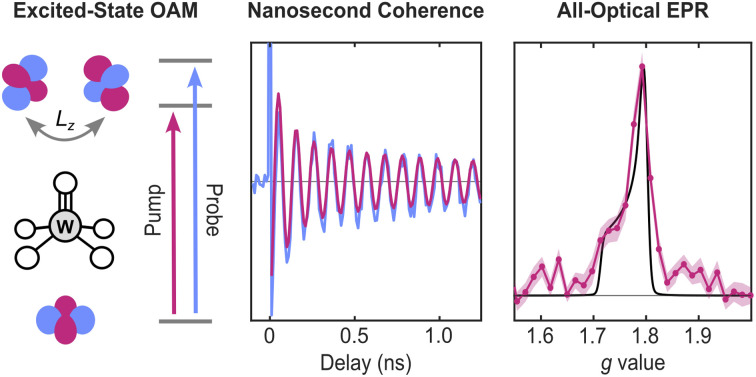

## Introduction

Molecular quantum sensing utilizes the electron spin as a quantum bit (qubit) to enable next-generation sensing modalities.^1–4^ The small size and synthetic tunability of molecules render them especially suitable for *in situ* microscopy relative to nitrogen-vacancy centers and other solid-state architectures,^5,6^ but such applications have heretofore remained elusive due to the lack of optical readout mechanisms and short coherence times, 
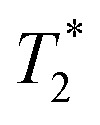
. Our group has aimed to address these drawbacks through the development of time-resolved Faraday ellipticity/rotation (TRFE/R), an ultrafast, all-optical approach to measure electron spin decoherence in the femto-to-nanosecond regime.^7,8^ With several orders of magnitude improvement in both temporal and spatial resolution over typical microwave techniques, TRFE/R has shown promise in elucidating the few-picosecond spin dynamics of octahedral (*O*_h_) iridium(iv) complexes in aqueous solutions at room temperature.^7,8^ In contrast, the fast decoherence of these complexes renders them microwave addressable only at very low temperatures. Optical addressability is achieved with a circularly polarized pump pulse to generate a spin-polarized ensemble of molecules, the decoherence of which is measured through the change in optical polarization of a subsequent probe pulse. Analysis of aqueous solutions and polymer films has highlighted the operative role of molecular tumbling as a primary driver of picosecond decoherence in [IrX_6_]^2−^ (X = Cl, Br). In addition to providing information on decoherence, TRFE/R enables all-optical detection of the molecular electronic *g* value—a highly informative magnetic resonance parameter—from free induction decays measured in a perpendicular magnetic field.

**Fig. 1:**
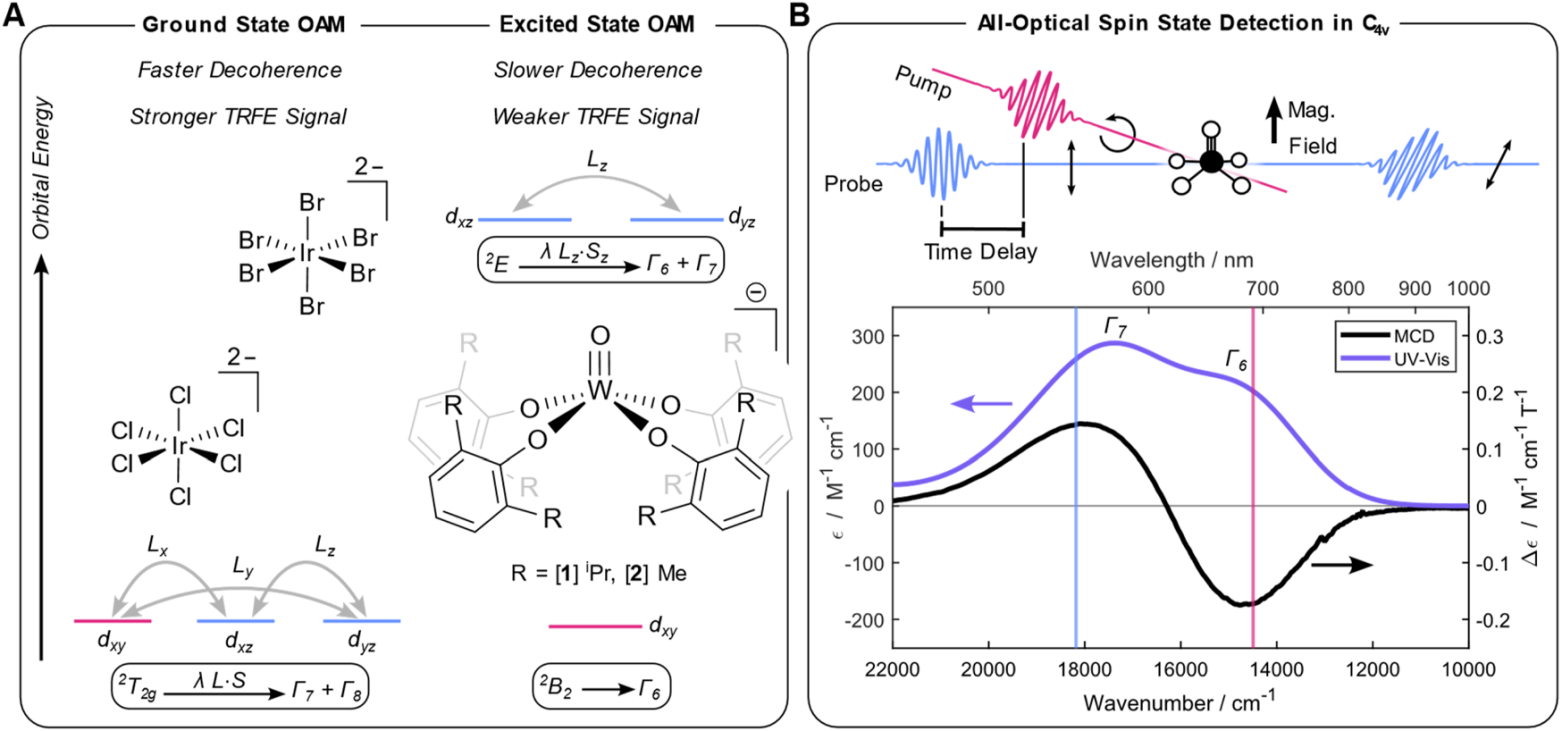
Relationship between OAM and spin dynamics. (A) Origins of GS and ES OAM. (B) Schematic of all-optical spin detection (top). Room-temperature electronic absorption and MCD spectra of 1 in THF (bottom); vertical lines represent pump (red) and probe (blue) wavelengths.

At present, a key limitation of TRFE/R is the dearth of molecular systems with suitable spin–photon coupling. Spin polarization induced by absorption of a circularly polarized photon is made possible through spin–orbit coupling (SOC) and orbital angular momentum (OAM), which presents a challenge in transition-metal complexes since the ligand field typically quenches the OAM of d electrons. However, this challenge can be overcome through judicious design of the coordination environment. In [IrX_6_]^2−^, the low-spin *O*_h_ d^5^ electronic structure leads to a triply orbital degenerate ^2^T_2g_ ground state (GS) formed from the {d_*xy*_, d_*yz*_, d_*xz*_} orbitals (Fig. 1A). These orbitals are related by 90° rotations about *x*, *y*, and *z*, representing an eigenstate of the OAM operators with effective (residual) OAM of *L*′ = 1. Through the efficient spin–photon interaction afforded by this large OAM, spin dynamics can be initialized and read-out optically even at few-micromolar Ir(iv) concentrations.^7^ However, ^2^T_2g_ GSs require high-symmetry molecules, limiting the potential to connect to the rich geometric and electronic structures of coordination complexes. Large GS SOC and OAM also drive spin decoherence,^9–11^ resulting in the picosecond 
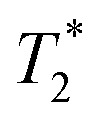
 observed for [IrX_6_]^2−^. Thus, establishing TRFE/R addressability in lower-symmetry systems represents a critical step toward expanding ultrafast, all-optical spin coherence spectroscopy across paramagnetic transition metals ranging from metallaphotoredox catalysts to metalloenzyme active sites.

One avenue to circumvent these issues is to utilize OAM in the excited state (ES). Such a mechanism was recently proposed by the Transue group for optically induced magnetization in a pseudo-*C*_4v_, d^1^ complex [Na(THF)_6_][WO(ODipp)_4_] (Fig. 1A, complex 1).^12^ This work provided detailed spectroscopic characterization using magnetic circular dichroism (MCD) and electron paramagnetic resonance (EPR), predicting strong GS spin polarization *via* circularly polarized excitation. However, direct measurements of optically induced spin polarization, including ultrafast spin decoherence measurements, have yet to be made.

Descent in symmetry from *O*_h_ to *C*_4v_ lifts the triple degeneracy of the t_2g_ orbitals, which results in a ^2^B_2_ (Γ_6_) GS (d_*xy*_) and a ^2^E ES (d_*xz*_/d_*yz*_). The d orbitals of the ^2^E state are eigenstates of the L_*z*_ operator, as they are related through a 90° rotation about the *z* axis, leading to modest ES OAM and SOC within the Γ_6_ and Γ_7_ states (*λ* = *ζ*_*W*(*V*)_ ≈ 3500 cm^−1^).^13^ MCD spectroscopy displays a clear signature of the ^2^E ES, with the two spin–orbit states showing intense, oppositely-signed bands forming a derivative-like shape—a pseudo-A term.^14,15^ Such line-shape is characteristic of degenerate (or nearly-degenerate) ES orbital manifolds related *via* rotation about a mutually orthogonal axis and indicates non-negligible ES OAM, rendering 1 an ideal candidate to test whether ES OAM may be leveraged for TRFE/R spectroscopy in axial systems.

Here we demonstrate nanosecond-scale spin coherence in room-temperature solutions of 1, including record sensitivities to DC magnetic fields. We further probe environmental factors to elucidate the mechanism of decoherence and demonstrate all-optically detected EPR spectroscopy.

## Results

**Fig. 2:**
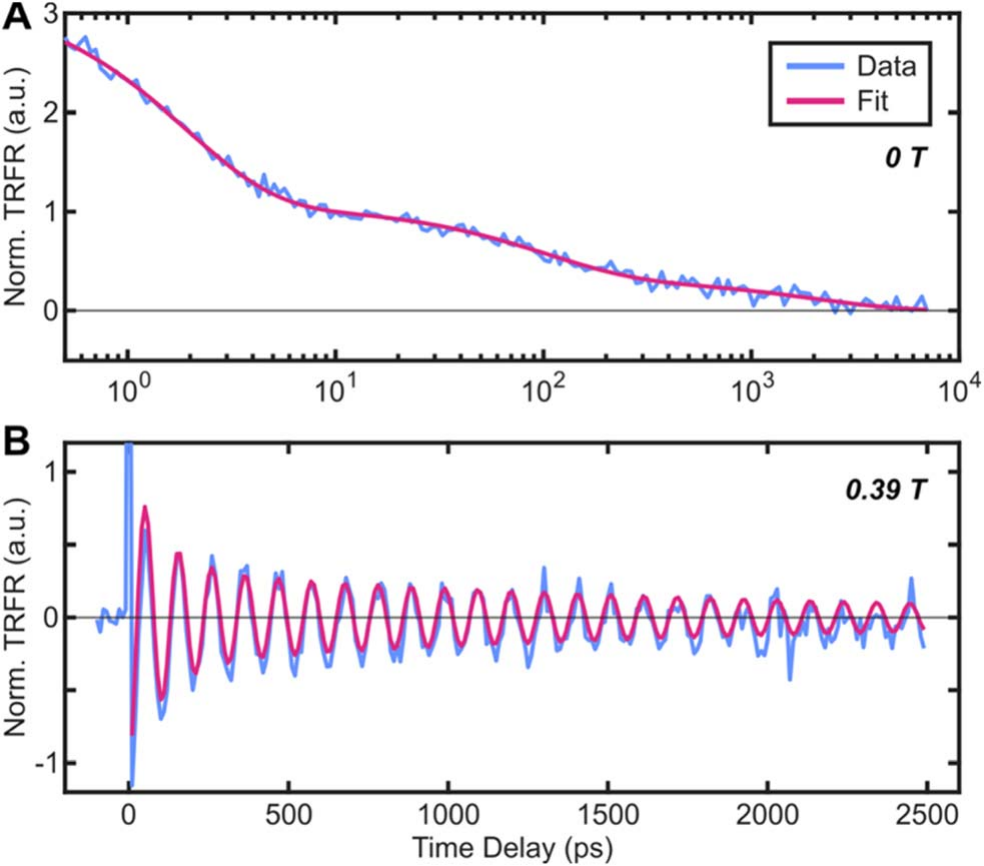
Ultrafast spin dynamics of 1. (A) Decoherence at 0 T. (B) Free induction decay at 0.39 T. Fit to both consists of a triexponential decoherence rate multiplied by a cosine at the Larmor frequency (0 Hz for (A)).

To test the excited-state OAM hypothesis and optically access the spin dynamics of 1 (11 mM in THF), we photoexcite between 680 and 700 nm (Γ_6_ → Γ_6_) and probe the TRFE/R response at 550 nm (Γ_6_ → Γ_7_). These pump/probe wavelengths reside within the steady state MCD pseudo-A term (Fig. 1B). Room-temperature TRFR measurements reveal a signal persisting for several nanoseconds (Fig. 2A), orders of magnitude longer than high-symmetry Ir(iv) complexes.^7,8^ Application of a perpendicular magnetic field (0.39 T for consistency with EPR) induces a clear free induction decay (Fig. 2B), demonstrating that the long-lived signal corresponds to spin polarization. Damped cosine fitting reveals *g*_iso_ = 1.76, consistent with the continuous-wave (CW) EPR spectrum at 77 K in 2-MeTHF (Fig. S4, *g*_iso_ = (*g*_‖_ + 2*g*_⊥_) = 1.772). Thus, this represents the first measurement of ultrafast electron spin coherence in a non-*O*_h_ molecule.

The 0 T decoherence profile fits well to a tri-exponential decay with time constants *τ*_1_ = 1.9(1) ps, *τ*_2_ = 100(16) ps and *τ*_3_ = 2.0(5) ns (error estimated from least-squared fit). Predictably, the signal intensity is noticeably weaker than [IrX_6_]^2−^ due to reduced OAM, limiting the accuracy of the fitted time constants. Beyond the intensity, the fit itself contrasts with the purely monoexponential decays observed in [IrX_6_]^2−^ and the stretched exponentials common in pulse EPR. To determine the origin of these components, we carried out further TRFE/R spectral characterization at 0 T (Fig. S39). With 550 nm probe, TRFE/R give identical traces, albeit with a larger intensity for the former. With a 500 nm probe, the 1.9 ps component switches sign relative to the other two, and no change in this initial component is observed in an applied magnetic field (Fig. S40), unlike *τ*_2_ and *τ*_3_. Thus, *τ*_1_ is not related to spin dynamics, and subsequent analyses focus on the >10 ps time regime, where only GS spin coherence is present. Transient absorption spectroscopy reveals a short electronic lifetime similar to *τ*_1_, suggesting a potential ES origin for the artefact (Fig. S41).

**Fig. 3:**
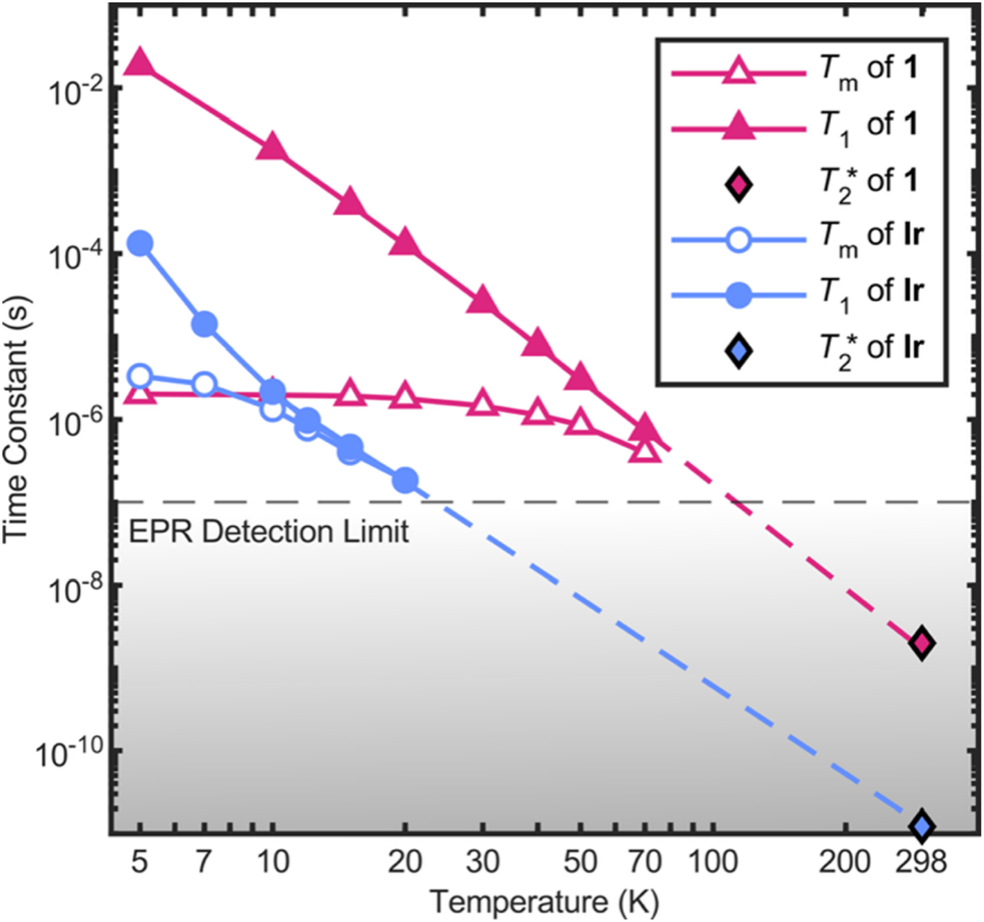
Temperature-dependent spin dynamics. Pulse EPR (403.2 mT) and TRFR (longest component) measurements for [IrBr_6_]^2−^ in 3 : 2 H_2_O : glycerol^8^ and 1 in 2-MeTHF (pulse EPR) or THF (TRFR).

Across a variety of molecular qubit systems, the spin-lattice relaxation rate (1/*T*_1_) imposes an upper limit on decoherence and is proportional to the amount of residual GS OAM.^11,16,17^ Consequently, X-band pulse EPR *T*_1_ values for [IrBr_6_]^2−^, obtained *via* inversion recovery echo sequences at *g*_iso_ in 3 : 2 water : glycerol, are short and fall below the EPR detection limit (∼100 ns) above 20 K (Fig. 3, blue).^8^ By contrast, pulse EPR of 1 in glassy 2-MeTHF at *g*_‖_ (0.40 T) reveals measurable *T*_1_ values up to 70 K (Fig. 3, red), remaining ∼100 times longer than [IrBr_6_]^2−^ at temperatures where direct comparison is possible. Similar *T*_1_ behavior is observed at *g*_⊥_ (0.38 T) (Fig. S11). Extrapolation of *T*_1_ to room temperature yields qualitative agreement with the observed disparity in TRFR decay rates. For both complexes, the spin–spin decoherence times measured *via* Hahn echo sequences (*T*_m_) become *T*_1_-limited at higher temperatures, though this does not necessarily mean 
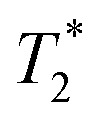
 is dictated solely by *T*_1_ in room-temperature solutions. Unlike 
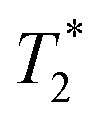
, *T*_m_ is insensitive to static inhomogeneity, but both are still subject to spectral diffusion and slow dynamic noise and so are approximations of the true spin–spin decoherence time *T*_2_. In [IrBr_6_]^2−^, for instance, immobilization in a polymer matrix extended 
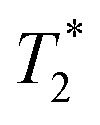
 by an order of magnitude relative to aqueous solution, while viscous water : glycerol solutions displayed intermediate decoherence rates. This suggests the question of whether room-temperature decoherence in 1 is similarly suppressed below the *T*_1_ limit by molecular tumbling or other means.

Having demonstrated TRFR addressability in 1, we sought to elucidate the mechanisms underlying decoherence. While these are increasingly well understood on timescales accessible by pulse EPR, there is little experimental evidence detailing sub-nanosecond decoherence mechanisms in solution. To investigate the impact of molecular tumbling on the observed 
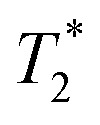
 in 1, polystyrene was added to the THF solution to raise the bulk viscosity from an estimated 0.48 cP to ∼13 cP.^18^ No change in 
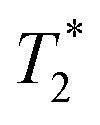
 was observed (Fig. S42). However, this could be due to polystyrene modulating only the bulk viscosity while minimally altering the THF solvation sphere directly surrounding the molecule. The chemical sensitivity of the complex currently precludes the use of other viscous small molecules, such as glycerol, to raise the microscopic viscosity.

With 
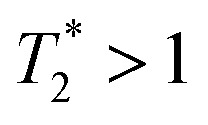
 ns, diffusion-mediated dipole–dipole interactions between electron spins could lead to decoherence. Indeed, the low-temperature pulse EPR shows a decrease in *T*_m_ with concentration (Fig. S12 and S13). However, no change in 
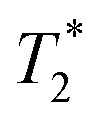
 could be detected above noise at room temperature across a four-fold increase in concentration (Fig. S43).

Hyperfine interactions between the electron spin and adjacent nuclear spins can lead to decoherence, especially with the large gyromagnetic ratio of ^1^H atoms.^19,20^ To address this, deuterated THF was used to remove protons from solvation. However, no change in 
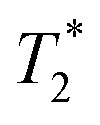
 was observed (Fig. S44). This lack of spin-interaction with solvation is unsurprising given the large steric bulk of the molecule likely providing significant shielding. However, the proton-rich 2,6-diisopropylphenoxide (ODipp) ligands raise the possibility of interaction with proximal nuclear spins. To reduce the number of W-adjacent protons, we replaced the ODipp with 2,6-dimethylphenoxide (ODmp) to form complex 2 (Fig. 1A and SI Section 7). This caused a slight blueshift of the electronic transitions (Fig. S1), likely due to the reduced steric bulk causing a slight contraction of the W–O bonds, but the electronic structure remained broadly the same. Despite this structural modification and associated changes to low-temperature decoherence (Fig. S14 and S15), the room-temperature spin dynamics are identical (within noise) to that of 1 (Fig. S45).

Given the solution-phase nature of these measurements, the solvent itself provides a means to tune the local chemical environment. Butyronitrile has the same viscosity as THF but a significantly larger dielectric constant. However, 
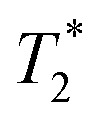
 of 1 is not sensitive to solvent dielectric and does not change significantly in any other solvent tested (Fig. S46).

We further sought to reduce molecular tumbling by encapsulating 1 in a polymethylmethacrylate (PMMA) matrix. This thin-film sample also has a relatively high effective concentration, which suppresses *T*_m_ at low temperature (Fig. S12 and S13). The CW EPR and electronic absorption spectra remain largely unchanged (Fig. S2 and S7), however, aside from modest peak broadening, indicating that the electronic structure is mostly preserved.

**Fig. 4:**
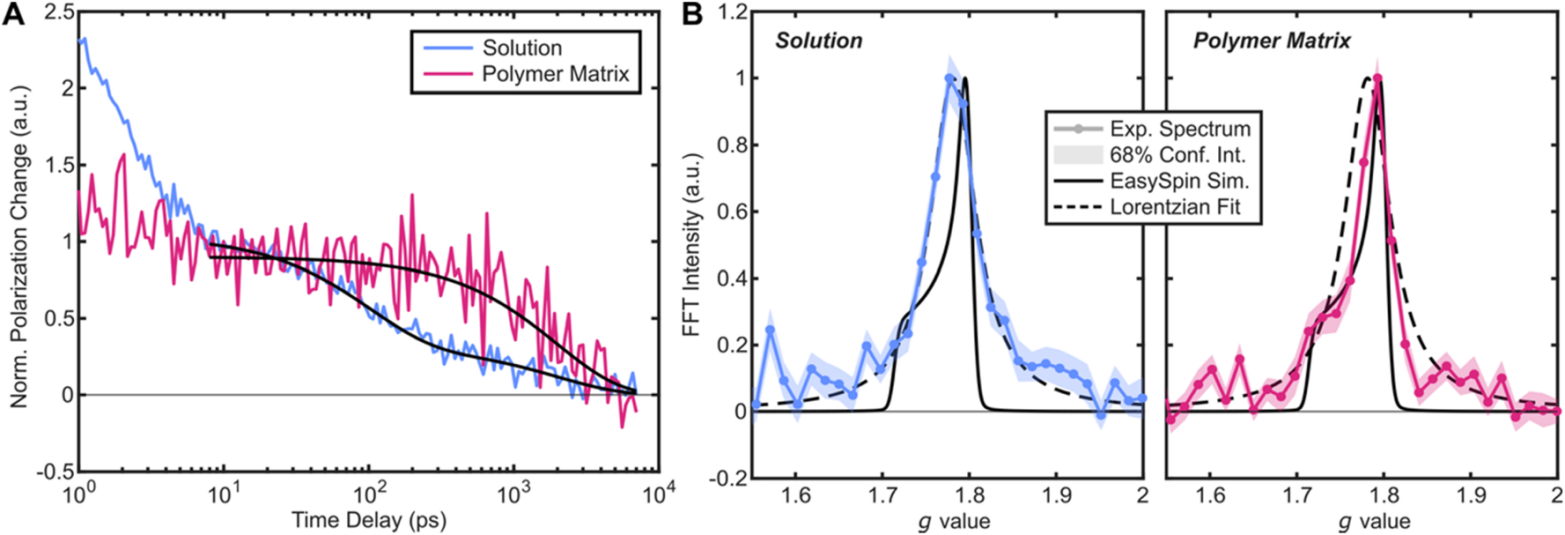
Effect of immobilization on spin dynamics. (A) Decoherence at 0 T for 1 in 11 mM THF solution and a PMMA polymer matrix using TRFR and TRFE, respectively. (B) Ultrafast, all-optical EPR spectra at 5 T for solution and film, error bounds given by standard error on mean. A linear background was subtracted from the spectra to aid comparison to the simulation.

At 0 T, the room-temperature thin film exhibits significantly different spin dynamics than the solution-phase sample (Fig. 4). In particular, the ∼100 ps decay component is absent, and the film data are well described by a single exponential with a time constant of ∼2 ns, matching the slowest component of the solution-phase decay.

At 5 T, both the solution and polymer-matrix samples exhibit long-lived free induction decays, although the noise level prevents reliable time-domain fitting (Fig. S48). The Fourier transform, however, reveals the spectral information enabled by the long 
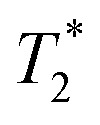
 and a narrow EPR linewidth (Fig. 4B and C). For freely rotating molecules in solution, with their rotational correlation time 
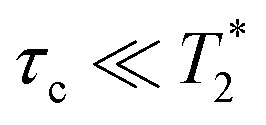
, a Lorentzian lineshape centered on the isotropic *g* value is expected. This is exactly what is observed for 1 in THF: a Lorentzian fit reproduces the spectrum almost perfectly, yielding *g*_iso_ = 1.78, and the linewidth corresponds to 
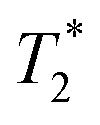
 ≈ 450 ps.

By contrast, the thin-film spectrum exhibits additional structure and is poorly described by a Lorentzian lineshape. Using spin Hamiltonian parameters obtained from EasySpin simulations of the axial 77 K CW EPR spectrum (Fig. S6 and Table S8), we simulated the expected 5 T spectrum for a frozen solution.^21^ The simulated and experimental lineshapes show clear correspondence: the sharp maximum at 1.80 aligns with *g*_⊥_, and the shoulder at 1.71 matches *g*_‖_. Although noise prevents quantitative fitting, and some discrepancy is expected because the spin Hamiltonian can be slightly perturbed across such a large temperature range, the overall agreement is clear.

These results show that *g*-value anisotropy can be observed all-optically in the time domain at room temperature, even when 
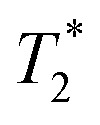
 remains several orders of magnitude below the sensitivity limit of pulse EPR. Optically detected *g*-value anisotropy has previously been reported in bulk CuSO_4_·5H_2_O using magnetic THz dichroism,^22^ but the weak absorption cross section for THz magnetic dipole transitions makes this approach less attractive for sensing than TRFE/R.

**Fig. 5:**
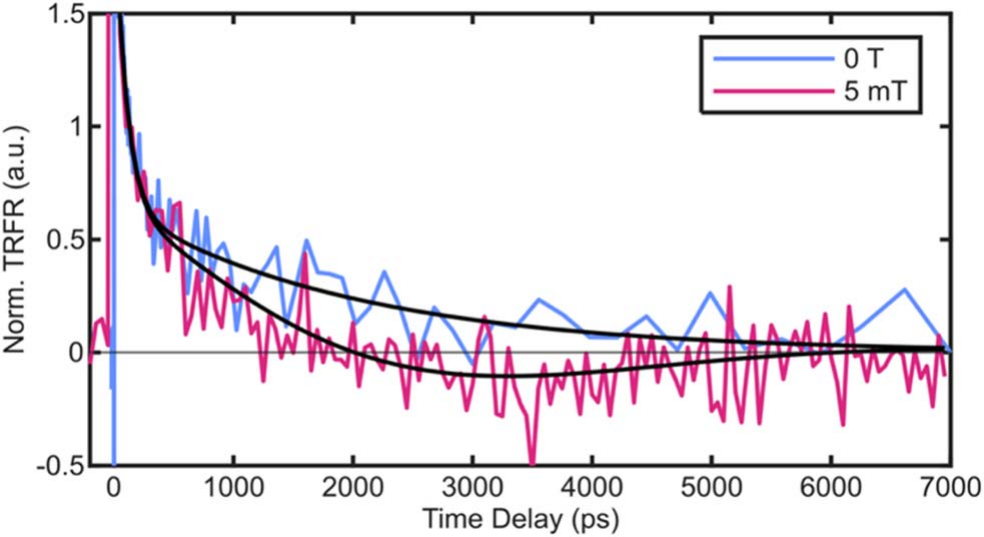
Ultrafast DC magnetic field sensing. Free induction decay for room temperature solution of 1 under 5 mT applied field.

Finally, motivated by the environmental robustness of 
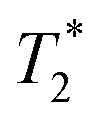
, we tested the limit of time-domain DC magnetic field sensing in solution-phase 1. Fields as low as 5 mT are readily detected (Fig. 5); even smaller changes may be measurable with an appropriate bias field. To our knowledge, this represents the best DC magnetic-field sensitivity yet reported for a molecule that does not rely on a radical pair mechanism.^23^

## Discussion

Room-temperature electron spin decoherence in 1 is biexponential and remains unchanged with spin concentration, solvent dielectric, solvent deuteration, and the number of ligand protons. Immobilization in a polymer matrix removes the fast 100 ps decay component while leaving the slower ∼2 ns component unchanged. Because molecular tumbling and spin–spin interactions primarily affect *T*_2_, these results instead point to *T*_1_-limited decoherence for the ∼2 ns component, consistent with the convergence of *T*_1_ and *T*_m_ in Fig. 3. This behavior differs from that of the [IrX_6_]^2−^ and [Cu(H_2_O)_6_]^2+^ complexes previously studied with TRFE, where decoherence is also driven by molecular tumbling and spin–spin interactions, respectively.^7,8,24^ The environmental insensitivity of 
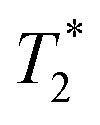
, together with the strong temperature dependence of *T*_1_, also suggests that this qubit may be well suited for temperature sensing and probing ultrafast, light-induced vibrational dynamics, especially given that decoherence can now be measured on the same timescales as vibrational relaxation processes.^25,26^

The disappearance of the ∼100 ps component in the film, however, merits further discussion. Because immobilization largely removes molecular tumbling, the two effects are likely linked. In *C*_4v_, the ^2^B_2_ → ^2^E transition is (*x*, *y*)-polarized, so pump and probe absorption should be strongest for molecules aligned favorably with the photon wavevectors. Through this orientational selectivity, the spin-polarized ensemble of molecules generated by the pump pulse will also be anisotropically oriented. This anisotropy should relax to isotropy on the timescale of *τ*_c_. However, because the GS OAM is low, we expect that molecular rotations will only couple weakly to the spins, allowing the spin ensemble to remain polarized during this process. The probe is subject to the same orientational selection rules, so the initially anisotropic ensemble absorbs more strongly than an isotropic one. In the extreme axial limit, the TRFE/R signal, *η*(*t*), will follow1
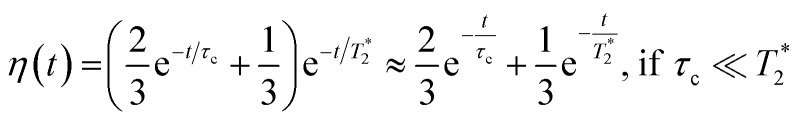


For a fully isotropic distribution, only one third of the molecules are favorably aligned with the light at any given time, producing a decay depending on *τ*_c_. In the polymer matrix, *τ*_c_ is significantly prolonged, leading to a monoexponential decay. The orientational selectivity in this complex is likely not perfectly strict, hence the observation of both *g*_‖_ and *g*_⊥_ in Fig. 4c. However, it is still non-zero, which would only reduce the difference in amplitudes of the two terms in eqn (1). Thus, the ∼100 ps and ∼2 ns decay components could correspond to *τ*_c_ and 
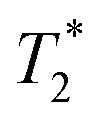
, respectively. Given the large size of 1, such a relatively long *τ*_c_ is not unreasonable. However, further experiments, ideally with systematic tuning of *τ*_c_, would be needed to confirm this hypothesis. Further discussion of potential origins of the biexponential decoherence and contributions from methyl rotors is given in SI Section S6.^28,29^

## Conclusions

In this work, we establish [WO(OR)_4_]^−^ as an optically addressable, room-temperature electron spin qubit. By leveraging ES rather than GS OAM, ultrafast spin dynamics become accessible in lower-symmetry, ligand-tunable molecular complexes rather than only purely octahedral inorganic salts. This strategy also offers a practical advantage in replacing Ir with the more abundant W. Lowering coupling between the spin and its environment by moving OAM to the ES extends 
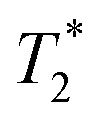
 by nearly two orders of magnitude, showing that a structure–function relationship borne out by prior pulse EPR studies extends to the picosecond regime. The resulting robustness of 
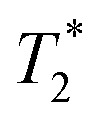
 to environmental perturbation, together with the ability to selectively detect DC magnetic fields down to 5 mT, highlights the sensing promise of this platform. Moreover, if 
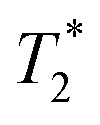
 is indeed *T*_1_-limited, the strong temperature dependence of *T*_1_ suggests a complementary role in all-optical temperature sensing and probing ultrafast vibrational relaxation mechanisms. Finally, immobilization in a polymer matrix removes rotational averaging and provides a free induction decay encoding *g*-anisotropy. To our knowledge, this is the first ultrafast, fully optically detected EPR spectrum reported for a molecular complex. This work, therefore, enables a route to all-optical sensing of magnetic fields, temperature, and molecular spin structure in platforms compatible with both bulk and microscopic samples. Beyond quantum sensing, these results point to an all-optical approach for interrogating paramagnetic species in coordination chemistry, potentially extending magnetic resonance observables into regimes inaccessible to conventional EPR.

## Methods

### Synthesis

All reactions and sample preparations were performed using air-free Schlenk line or nitrogen glove box procedures. Dry solvents were obtained from a Pure Process Technology Solvent Purification System and then degassed and stored over molecular sieves in the glove box. 2,6-Diisopropylphenol (Thermo Scientific), sodium hexamethyldisilazide (Thermo Scientific), allyltrimethylsilane (Oakwood), 2,6-dimethylphenol (TCI America), and tungsten(vi) chloride (Strem) were used as received without further purification. [Na(THF)_6_][WO(ODipp)_4_] (1) and WOCl_3_(THF)_2_ were synthesized according to established procedures.^12,27^

Sodium 2,6-dimethylphenoxide (NaODmp) was prepared *via* deprotonation of 2,6-dimethylphenol (HODmp) with sodium hexamethyldisilazide (NaHMDS) in the glove box. In a 20 mL scintillation vial, 500 mg HODmp (4.09 mmol) was dissolved in 2 mL Et_2_O (solution A). In a separate vial, 790 mg (4.29 mmol) NaHMDS was dissolved in 5 mL Et_2_O (solution B). Both solutions were then frozen in the glove box cold well (77 K). Upon thawing, B was added dropwise to A while stirring. After stirring for another 30 minutes, the suspension was filtered, washed with hexanes (∼10 mL), and dried to yield a white powder (292 mg, 50%). ^1^H NMR (400 MHz, CD_3_CN) *δ* 6.72 (doublet, 2H, *meta*-CH), *δ* 6.00 (triplet, 1H, *para*-CH), 2.03 (singlet, 6H, methyl CH_3_).

[Na(THF)_4_][WO(ODmp)_4_] (2) was prepared in the glove box by reacting 1 eq. WOCl_3_(THF)_2_ with 4.5 eq. NaODmp in THF. In a 20 mL scintillation vial, 51.3 mg (0.113 mmol) WOCl_3_(THF)_2_ was dissolved in 4 mL THF (solution A). In a separate vial, 74.7 mg (0.519 mmol) NaODmp was dissolved in 3 mL THF (solution B). Both solutions were then frozen in the glove box cold well (77 K). Immediately upon thawing, B was added dropwise to A while stirring. After stirring for another 30 minutes, the reaction solution was passed through a syringe filter (0.2 µm pores). The solution was then concentrated under vacuum to ∼4 mL and placed in the glove box freezer (−40 °C) overnight, yielding blue crystals (30 mg, 27%).

### Polymer film preparation

A 50 mg mL^−1^ poly(methyl methacrylate) stock solution was prepared in the glove box by dissolving 118 mg PMMA (Beantown Chemical) in 2.36 mL MeCN and stirring overnight. An 11 mM solution of 1 was then prepared by dissolving 7.5 mg in 500 µL of the PMMA stock. Films were prepared from this solution *via* drop-casting on 1 × 1 cm glass slides (12 drops each). Immediately after drop-casting, the slides were covered with a mason jar to ensure slow evaporation. After 3 hours, transparent blue films were obtained and transferred to the glovebox freezer (−40 °C) for storage. For TRFE/R measurements, films were mounted in an airtight cryostat (Oxford Instruments MicrostatHe), removed from the box, and placed along the path of the laser while pulling vacuum on the cryostat to minimize air exposure. For CW and pulse EPR experiments, films were cut into thin strips and placed in a standard X-band EPR tube. To preserve a nitrogen atmosphere, a plug of vacuum grease was inserted at the upper end of the tube before removing it from the glove box.

### X-ray crystallography

Low-temperature diffraction data (*ϕ*-and *ω*-scans) were collected on a Bruker AXS D8 VENTURE KAPPA diffractometer coupled to a PHOTON II CPAD detector with Mo K_α radiation (*λ* = 0.71073 Å) from an IµS micro-source for the structure of compound V26039 (CSD 2550952). The structure was solved by direct methods using SHELXS and refined against *F*2 on all data by full-matrix least squares with SHELXL-2019 using established refinement techniques.^30–32^ All non-hydrogen atoms were refined anisotropically. All hydrogen atoms were included into the model at geometrically calculated positions and refined using a riding model. The isotropic displacement parameters of all hydrogen atoms were fixed to 1.2 times the *U* value of the atoms they are linked to (1.5 times for methyl groups). All disordered atoms were refined with the help of similarity restraints on the 1,2- and 1,3-distances and displacement parameters as well as enhanced rigid bond restraints for anisotropic displacement parameters.

[Na(THF)_4_][WO(ODmp)_4_] (CSD 2550952, data block V26039) crystallizes in the tetragonal space group *P*4/*ncc* with a quarter of a molecule in the asymmetric unit. The highest electron density maximum is located on the 4-fold rotation axis in a chemically unreasonable location and was not refined.

### EPR spectroscopy

X-band continuous-wave (CW) EPR spectra were collected using a Bruker EMX spectrometer with Bruker Xenon software. All spectra were recorded at a microwave power of 0.14 mW (below the power saturation threshold) and with a modulation amplitude of 2 gauss. All samples were measured at 77 K using a liquid nitrogen immersion dewar. Spin Hamiltonian fits (Table S1) were performed using the EasySpin pepper and esfit functions in MATLAB.

X-band pulse EPR measurements were collected using a Bruker ELEXSYS E580 EPR spectrometer fitted with a Bruker MD4 resonator. Liquid-helium temperature control was achieved with an Oxford Instruments Mercury ITC temperature controller alongside a CF935 flow cryostat. *T*_m_ measurements (Tables S5–S7) were obtained from the two-pulse Hahn-echo sequence (π/2–τ–π–τ–echo), in which the dephasing time *τ* was varied. Inversion-recovery measurements of *T*_1_ (Tables S2–S4) utilized a (π–t–π/2–τ–π–τ–echo) sequence, in which the relaxation time *t* was varied while holding *τ* constant. The duration of all π/2 pulses was 8 ns, while that of all π pulses was 16 ns. The Hahn-echo decay and inversion recovery measurements were collected using two-step and four-step phase cycling, respectively. At each temperature point, the video gain was optimized. The Hahn-echo decay and inversion recovery data were fit to monoexponential (eqn (2)) and stretched exponential (eqn (3)) functions, respectively. For frozen solution samples, Hahn echo decays were fit to 2*τ* > 800 ns to avoid artifacts from intense proton ESEEM features at low time delays.2
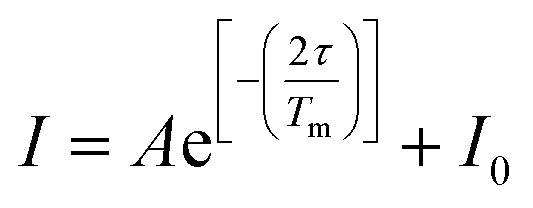
3
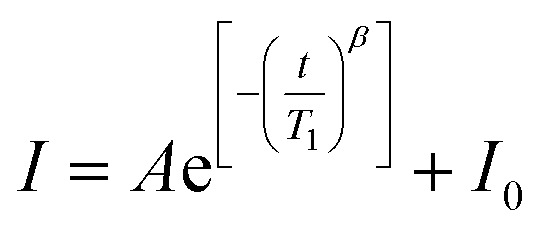


### Static optical spectroscopy

For all solution-phase optical measurements, the sample was held in an airtight quartz cuvette with 2 mm path length, filled under a nitrogen atmosphere. For the TRFE on the thin film, the sample was placed in an optical cryostat under a nitrogen atmosphere and then held under active vacuum over the course of the measurement. All measurements were carried out with the samples at room temperature.

Solution phase absorbance spectra were carried out using a Cary-5000 spectrophotometer. The UV-vis absorption of the film was recorded with a StellarNet fiber-optic spectrophotometer within the glove box.

MCD spectra on solutions were measured using a JASCO J-1700 circular dichroism spectrometer, equipped with a 1.4 T permanent magnet, again using a 2 mm path length cuvette. Circular dichroism spectra were collected for both parallel and antiparallel field directions, then subtracted and halved to leave only the MCD.

### Time-resolved optical spectroscopy

Full experimental details regarding the ultrafast setup are given in ref. 7 but are briefly described here. All transient measurements proceeded using the same Coherent Astrella fs laser system, which produced ∼35 fs pulses centered on 800 nm. Half the pulse power was used to pump an optical parametric amplifier (OPA, Coherent OPerA Solo) to generate pulses centered between 680 and 700 nm. These were used as the pump pulses, and the remaining fundamental was used as the probe, where a moveable retroreflector introduces a time delay in the probe relative to the pump. No difference was observed in TRFE between 680 and 700 nm pump (Fig. S47), and pulses centered on the two have significant overlap given the broad spectral width of the OPA output. Thus, these are used interchangeably throughout this article.

For TRFE/R, a supercontinuum was generated by focusing the heavily attenuated probe into a 2 mm sapphire crystal. After recollimation, the remaining 800 nm was removed by a 750 nm short-pass filter. From here the probe pulses were focused *via* a spherical mirror onto the sample and linearly polarized. After the sample, the transmitted and recollimated probe beam entered the balanced detection scheme. This consisted of either a half-wave plate (TRFR) or both half- and quarter-wave plates (TRFE), a Wollaston prism and a pair of photodiodes. The difference in intensity of the two photodiodes—measured with a lock-in amplifier—was proportional to the degree of polarization change in the probe. To select a particular probe wavelength, a 40 nm wide bandpass filter centered on the desired wavelength was placed immediately after the waveplates. The pump pulse was attenuated, chopped to remove every third pulse and circularly polarized by a photoelastic modulator (PEM) before being focused onto the sample. The sample itself was placed in the room-temperature bore of a superconducting magnet, allowing us to apply transverse fields up to 5 T. Unless noted otherwise, all solution-phase TRFE/R was carried out in THF at 11 mM using 1.1 µJ pump and 550 nm probe. All TRFE on the polymer films used 1.6 µJ pump and 550 nm probe. Traces are normalized to 10 ps to aid comparison.

For TA, the pump and probe lines detailed above were passed into an Ultrafast Systems Helios TA Spectrometer. The 800 nm probe generated a supercontinuum in CaF_2_, the spectrum of which was measured before and after the sample with CCD array spectrometers. The pump beam was chopped, attenuated, and depolarized before focusing onto the sample. A custom MATLAB script was used to perform chirp corrections, plotting, and global fitting of the data to a multi-exponential model.

## Author contributions

ES & JPA collected and analyzed the data. All authors contributed towards experimental design and writing.

## Conflicts of interest

There are no conflicts to declare.

## Supplementary Material

SC-OLF-D6SC04497B-s001

SC-OLF-D6SC04497B-s002

## Data Availability

Data supporting this article – including additional CW and pulse EPR spectra, TRFE decay traces, and transient absorption measurements – have been included as part of the supplementary information (SI). Raw data files are available from the corresponding author upon reasonable request. CCDC 2550952 (2) contains the supplementary crystallographic data for this paper.^33^ Supplementary information is available. See DOI: https://doi.org/10.1039/d6sc04497b.
