# Supplementary material for: Excited-state orbital angular momentum enables all-optical molecular spin coherence
Source: Chem Sci. 2026 Jun 26;17(30):14536–43. doi: 10.1039/d6sc04497b (PMC13326084; doi:10.1039/d6sc04497b)
Supplement: Supplementary file 1 [file SC-017-D6SC04497B-s001.pdf]

Supplementary Information (SI) for Chemical Science.
This journal is © The Royal Society of Chemistry 2026

Supporting Information

Excited-state orbital angular momentum enables all-optical molecular spin coherence

Erica Sutcliffe,[†] Jonathan P. Aalto,[†] Ryan G. Hadt*

Division of Chemistry and Chemical Engineering, Arthur Amos Noyes Laboratory of Chemical Physics, California Institute of Technology, Pasadena, California 91125, United States

[†]Denotes Equal Contributions

*Corresponding Author: rghadt@caltech.edu

Table of Contents

S1. Static Optical Spectroscopy 3
S2. Continuous-Wave Electron Paramagnetic Resonance (CW-EPR) 4
S3. Pulse Electron Paramagnetic Resonance (Pulse EPR) 8
S4. Time Resolved Faraday Spectroscopy (TRFE/R) 35
S5. Transient Absorption (TA) Spectroscopy. 39
S6. Discussion of Decoherence Mechanisms 40
S7. X-Ray Crystallography 41
S8. References. 43

S1. Static Optical Spectroscopy

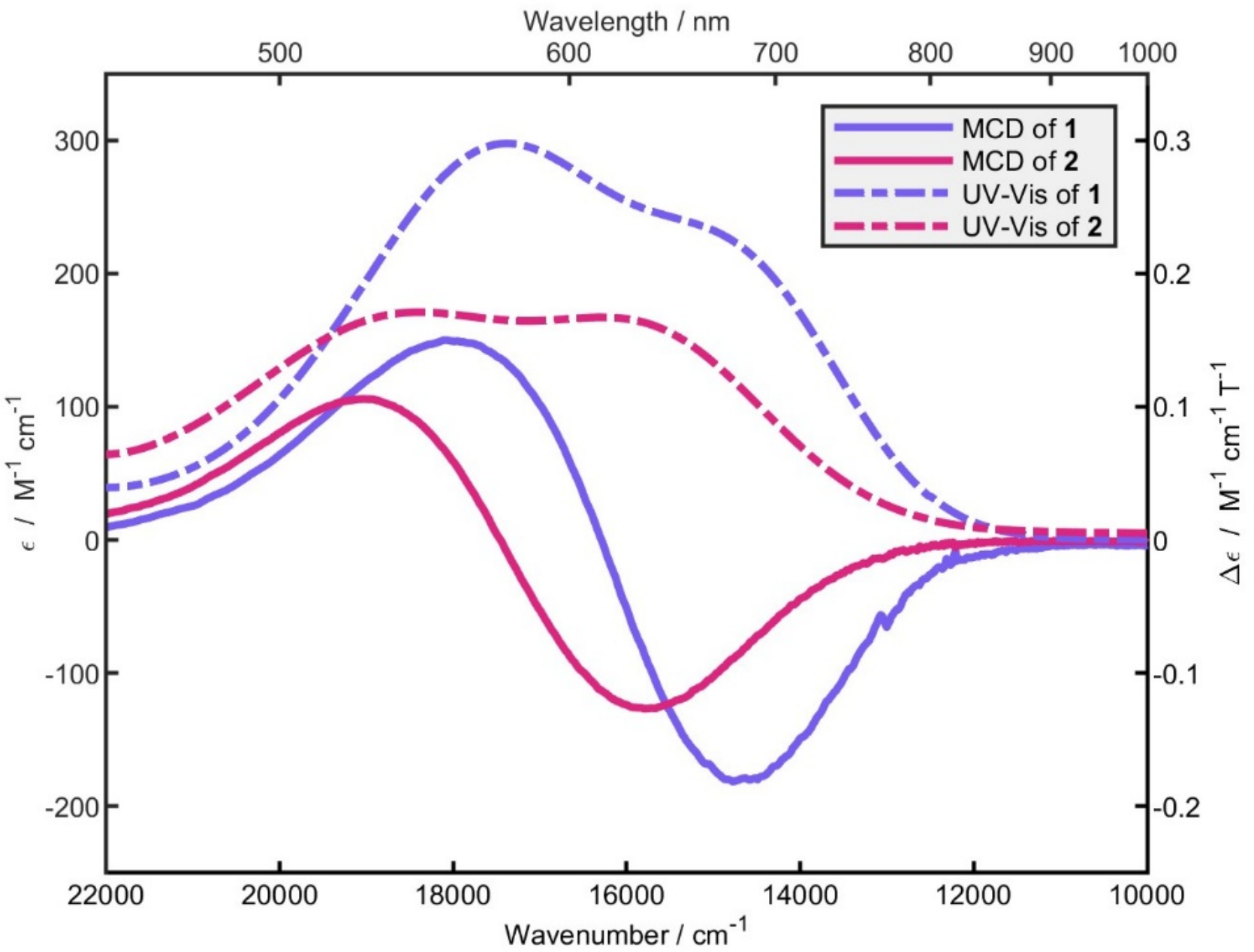

Figure S1: Absorption (dashed) and MCD (solid) spectra of **1** (purple) and **2** (pink) at 11 mM in THF. Collected using a Schlenk cuvette (2 mm path length) at room temperature.

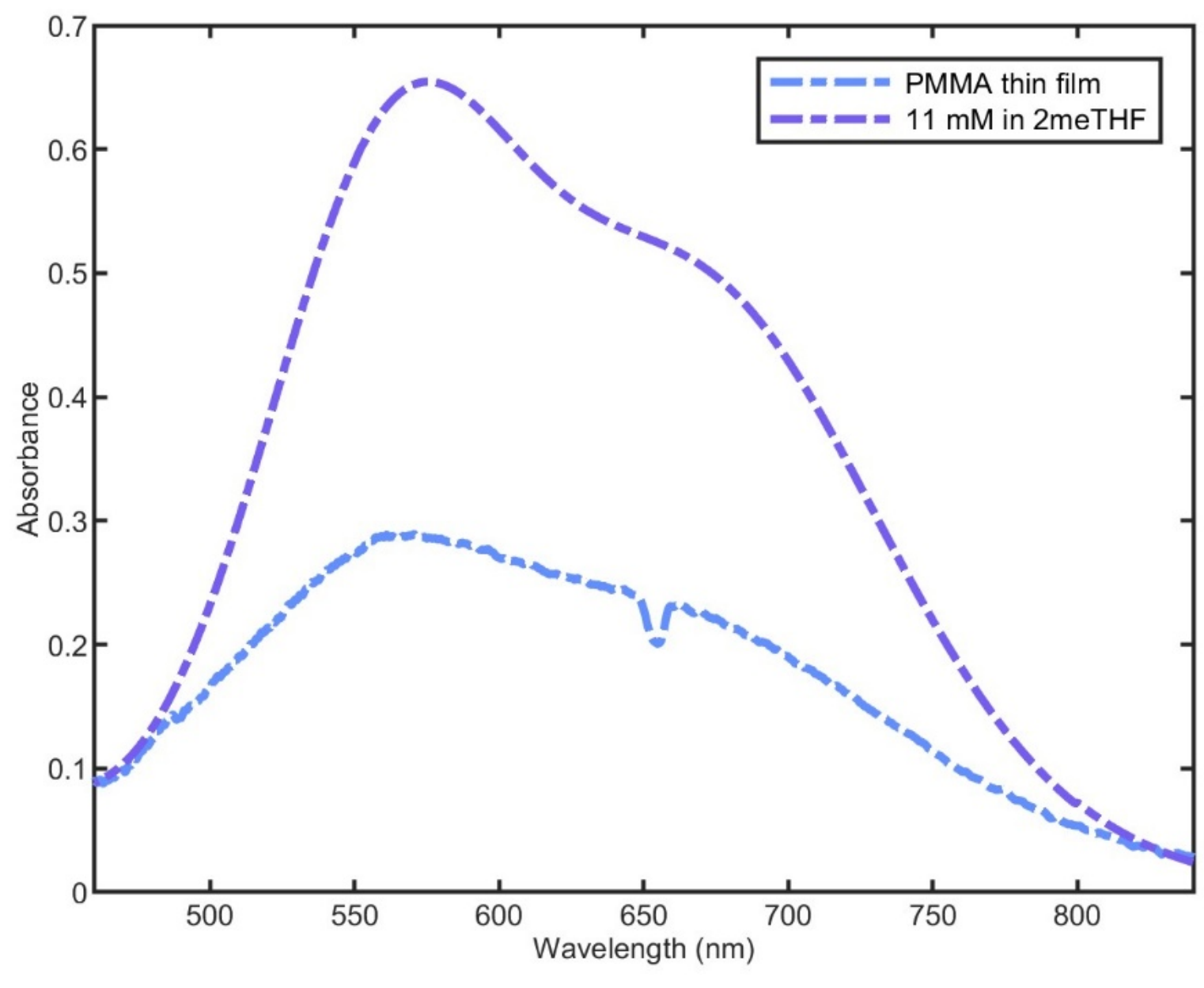

Figure S2: Absorption spectra of **1** in THF (11 mM, purple) and PMMA thin film (blue). The PMMA spectrum was collected by placing the film along the beam path of a StellarNet UV-VIS-NIR spectrometer in the glove box. Feature at 655 nm is an artifact of the setup.

S2. Continuous-Wave Electron Paramagnetic Resonance (CW-EPR)

Table S1: Spin Hamiltonian parameters from EasySpin (pepper) fits

Sample	$g_{\perp}$	$g_{\|\|}$	$A_{\perp}$	$A_{\|\|}$	$g_{strain,\perp}$	lw
**1** in 2-MeTHF (1.1 mM)	1.801	1.715	228	415	0.0033	1.29
**1** in 2-MeTHF (11 mM)	1.801	1.715	228	415	0.0034	1.30
**2** in 2-MeTHF (1.1 mM)	1.813	1.719	224	418	0.0071	1.66
**2** in 2-MeTHF (11 mM)	1.812	1.718	224	416	0.0074	1.68
**1** in PMMA thin film	1.798	1.707	230	410	0.0013	4.33
**1** in butyronitrile	1.799	1.715	229	415	0.0040	1.47
**1** in 1:1 THF:PhMe	1.801	1.715	229	412	0.0032	1.45

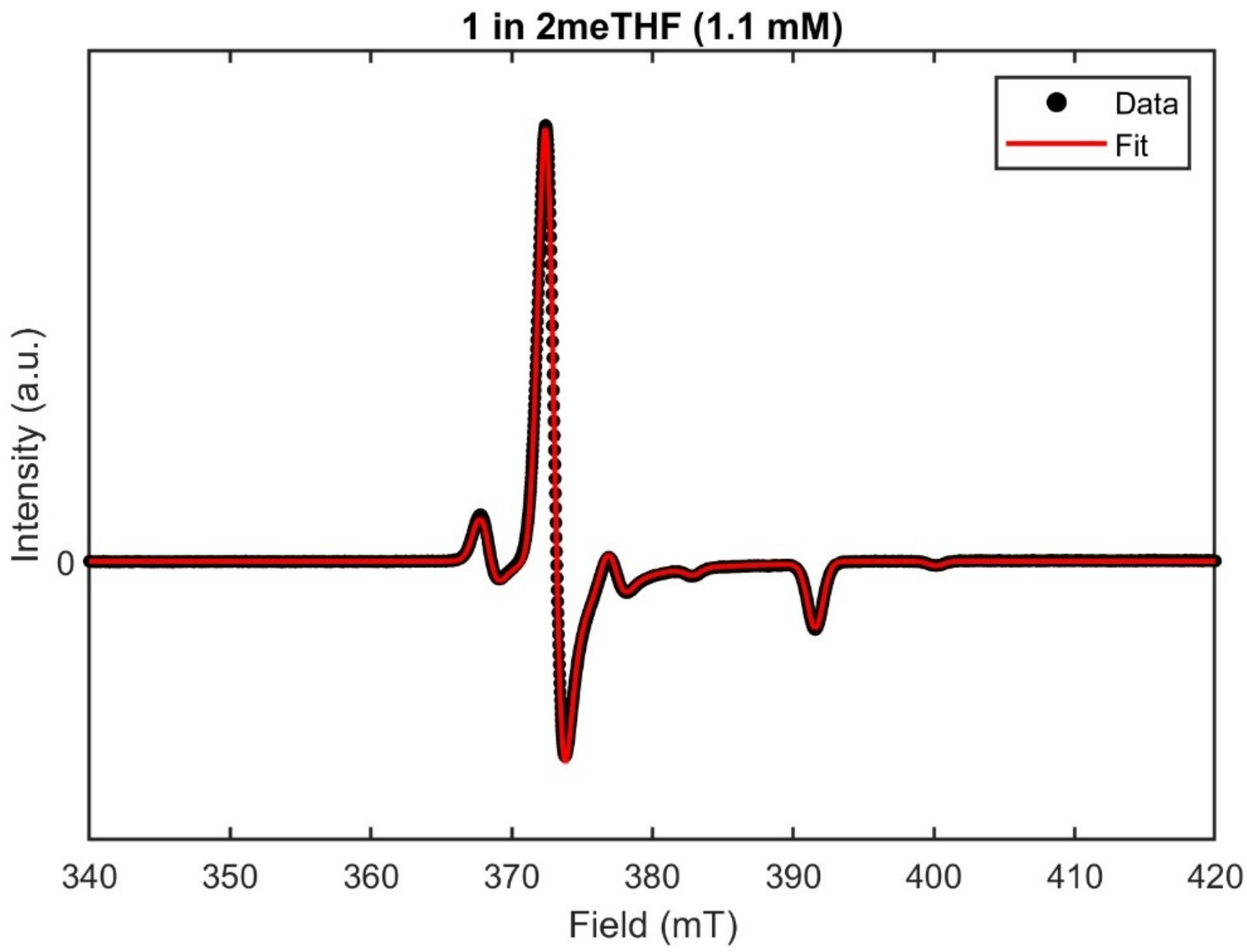

Figure S3: X-band (9.40 GHz) CW-EPR spectrum of **1** in 2-MeTHF (1.1 mM). Collected at 77 K with a modulation amplitude of 2 Gauss and a microwave power of 0.14 mW.

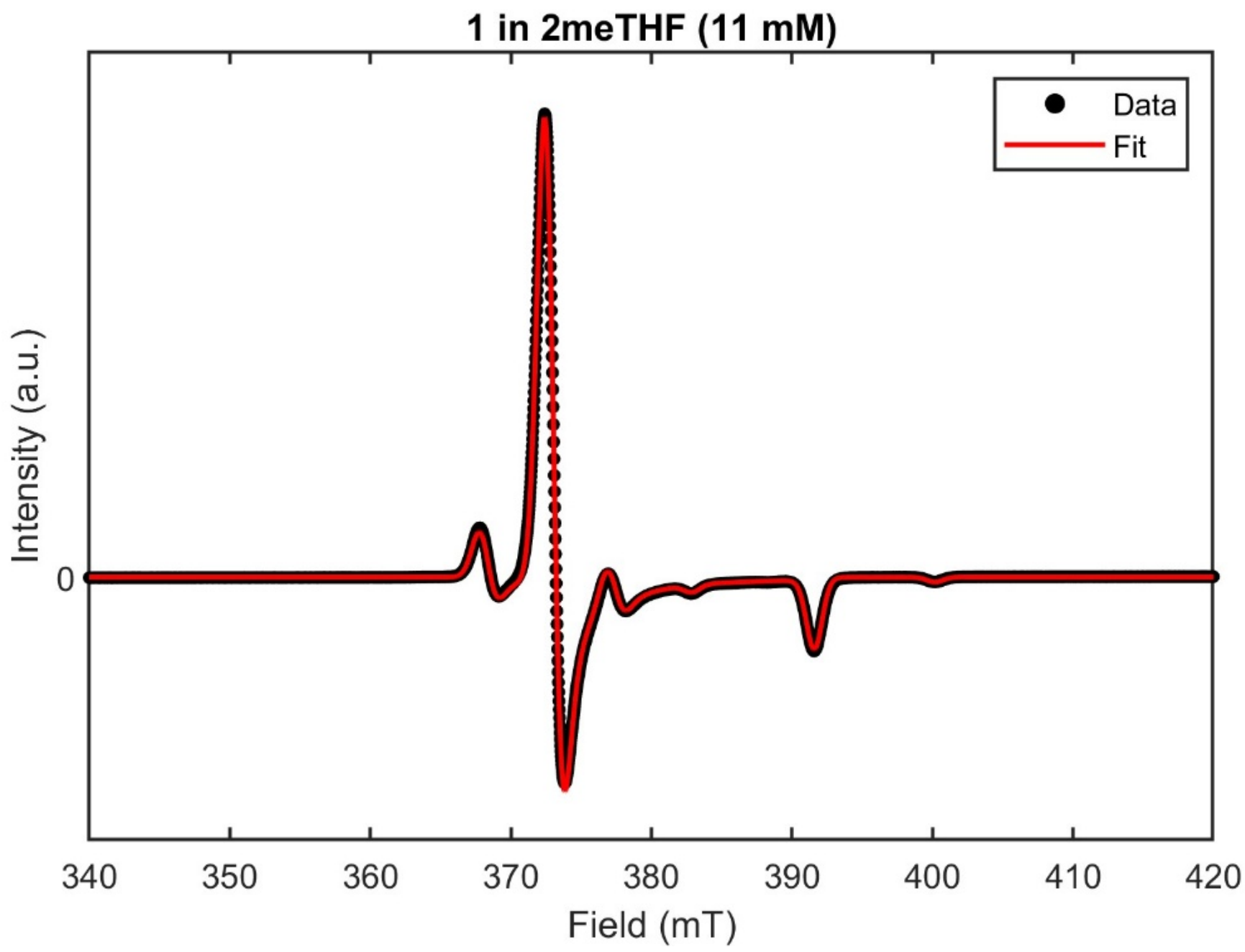

Figure S4: X-band (9.40 GHz) CW-EPR spectrum of **1** in 2-MeTHF (11 mM). Collected at 77 K with a modulation amplitude of 2 Gauss and a microwave power of 0.14 mW.

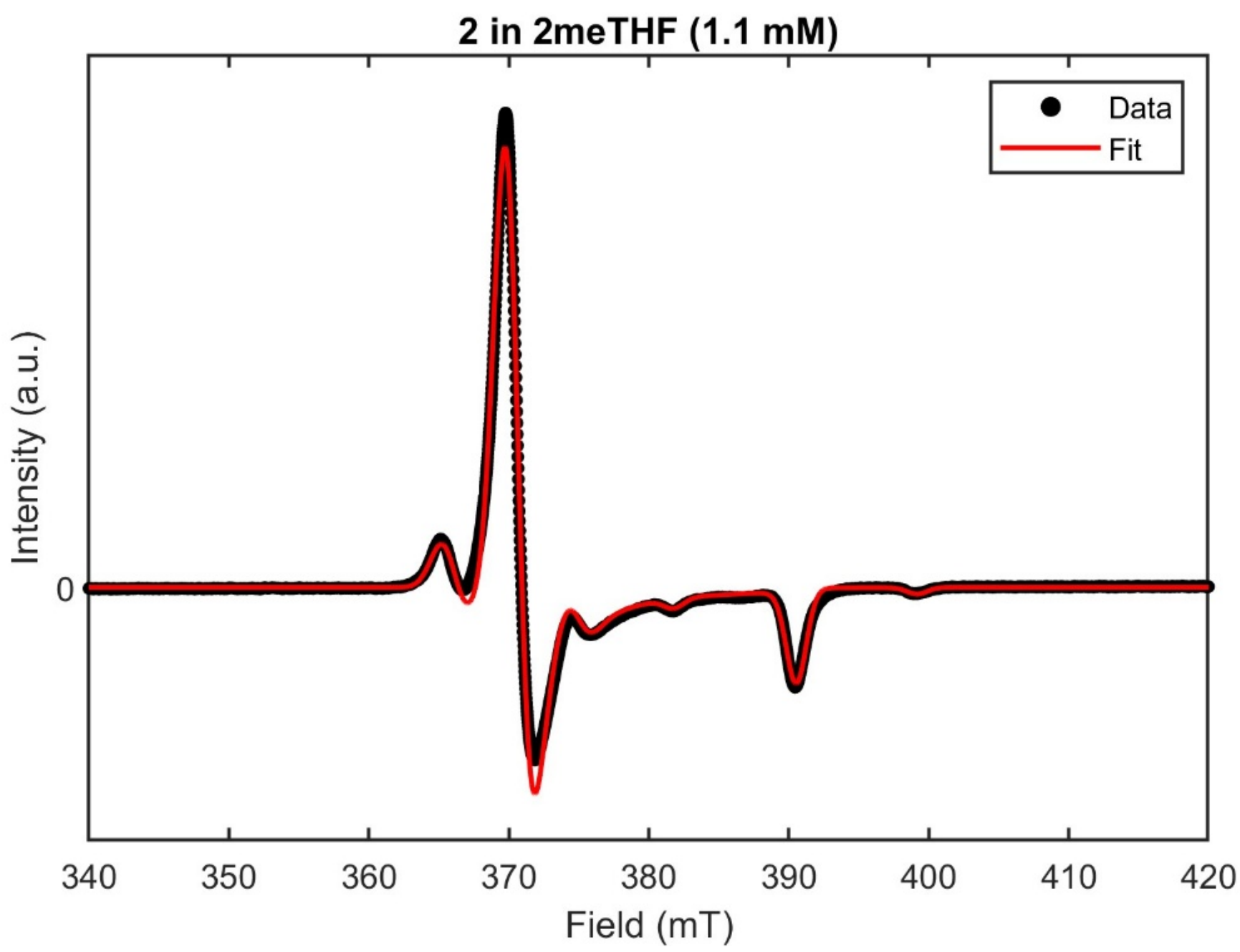

Figure S5: X-band (9.39 GHz) CW-EPR spectrum of **2** in 2-MeTHF (1.1 mM). Collected at 77 K with a modulation amplitude of 2 Gauss and a microwave power of 0.14 mW.

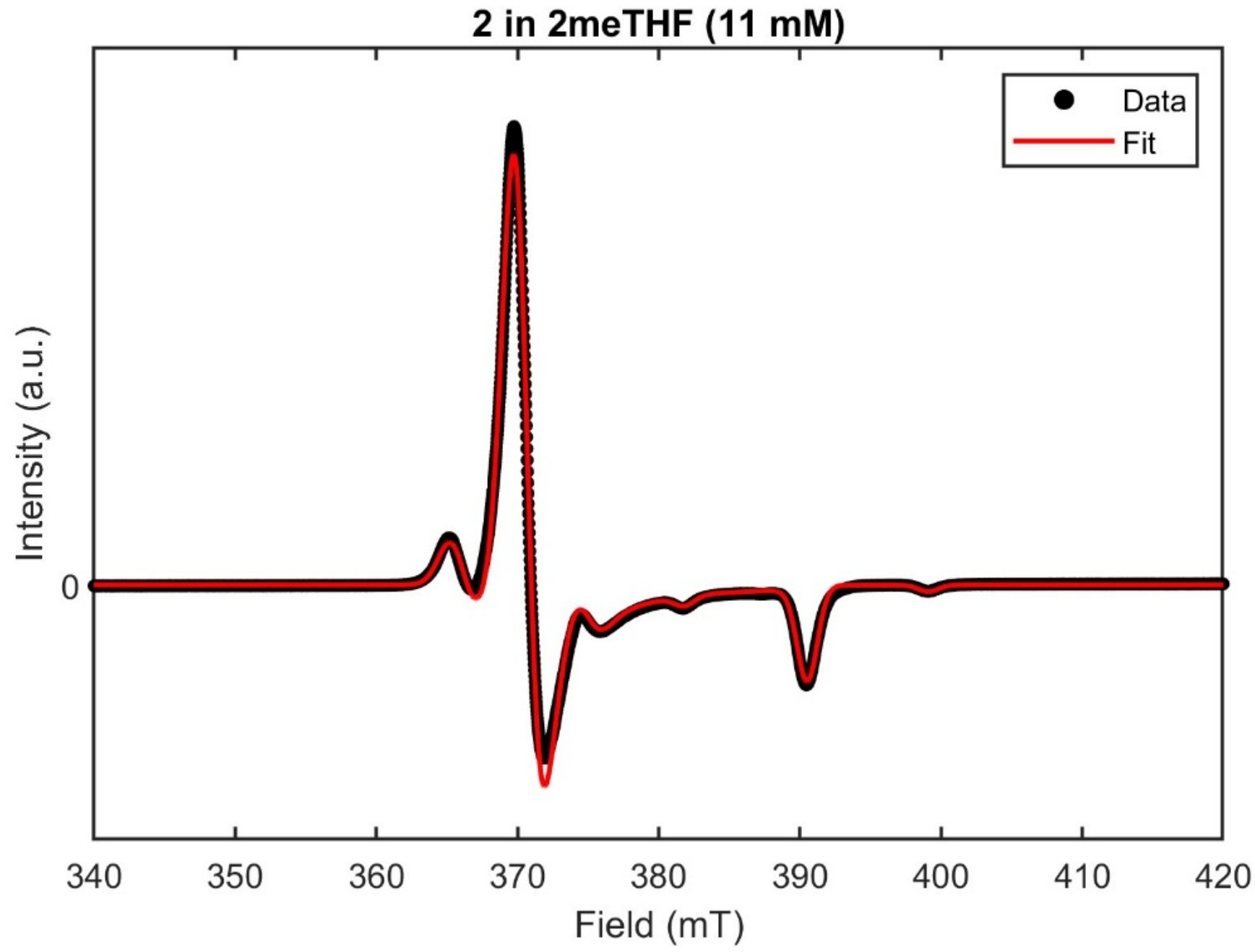

Figure S6: X-band (9.39 GHz) CW-EPR spectrum of **2** in 2-MeTHF (11 mM). Collected at 77 K with a modulation amplitude of 2 Gauss and a microwave power of 0.14 mW.

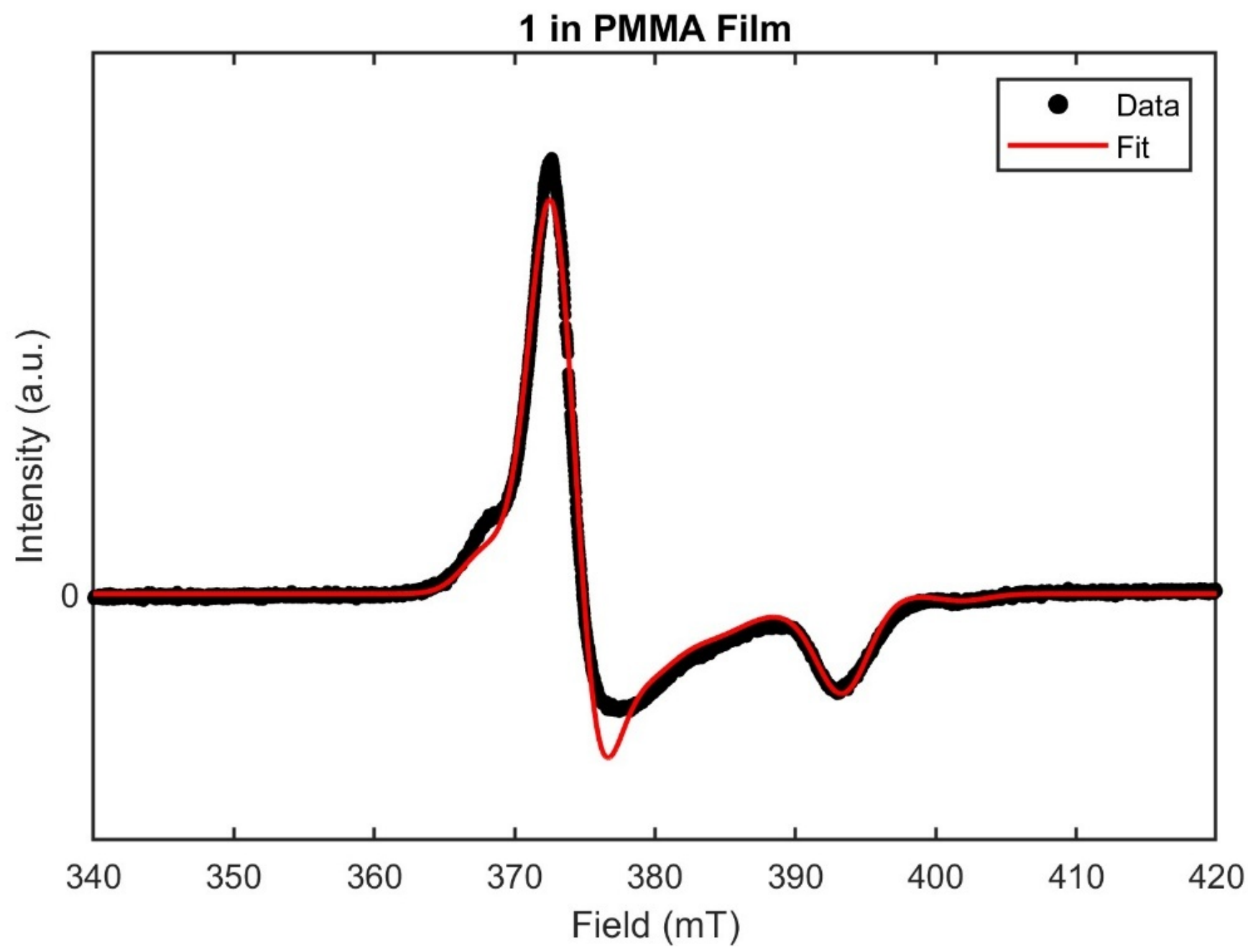

Figure S7: X-band (9.40 GHz) CW-EPR spectrum of **1** in PMMA thin film. Collected at 77 K with a modulation amplitude of 2 Gauss and a microwave power of 0.14 mW.

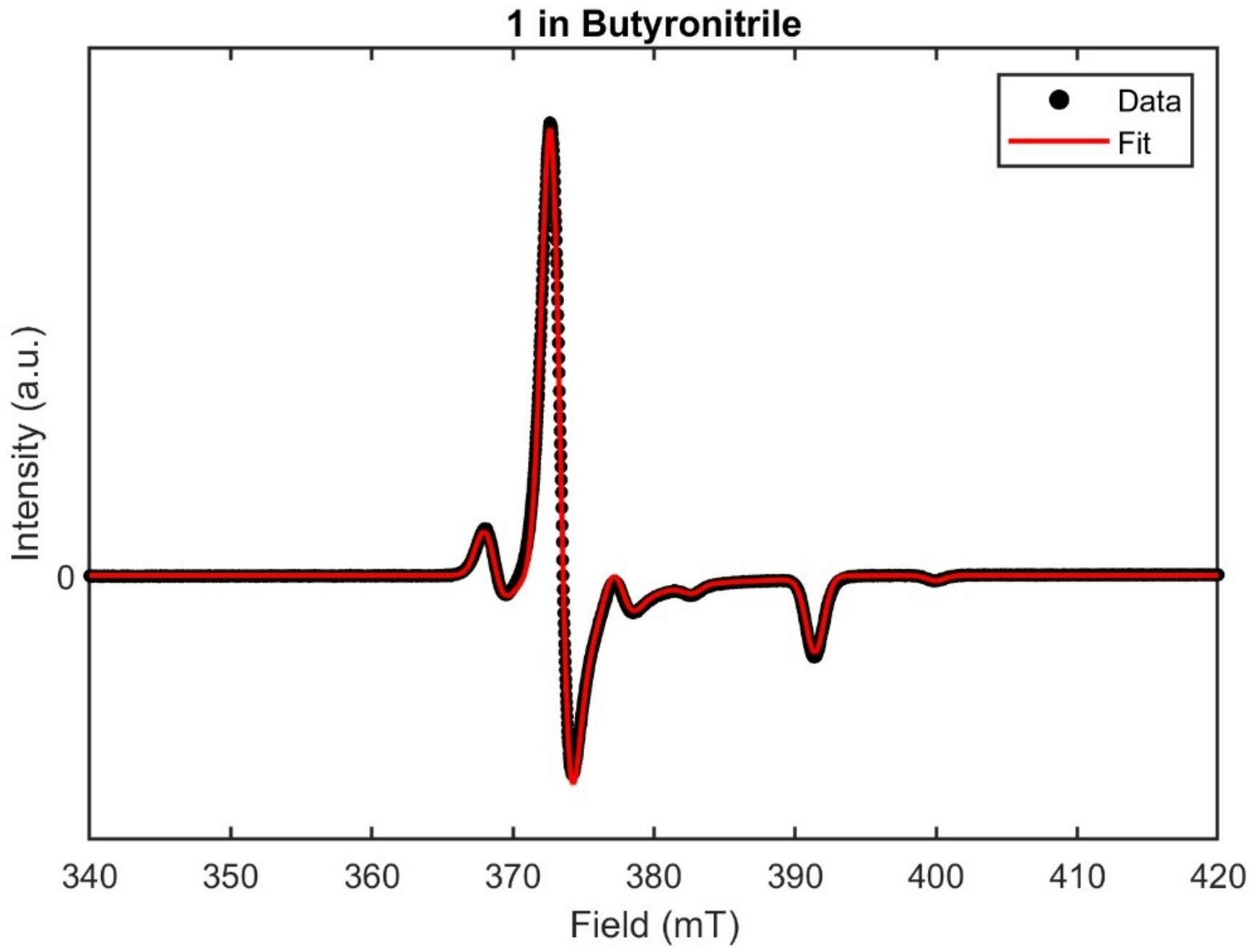

Figure S8: X-band (9.39 GHz) CW-EPR spectrum of **1** in butyronitrile. Collected at 77 K with a modulation amplitude of 2 Gauss and a microwave power of 0.14 mW.

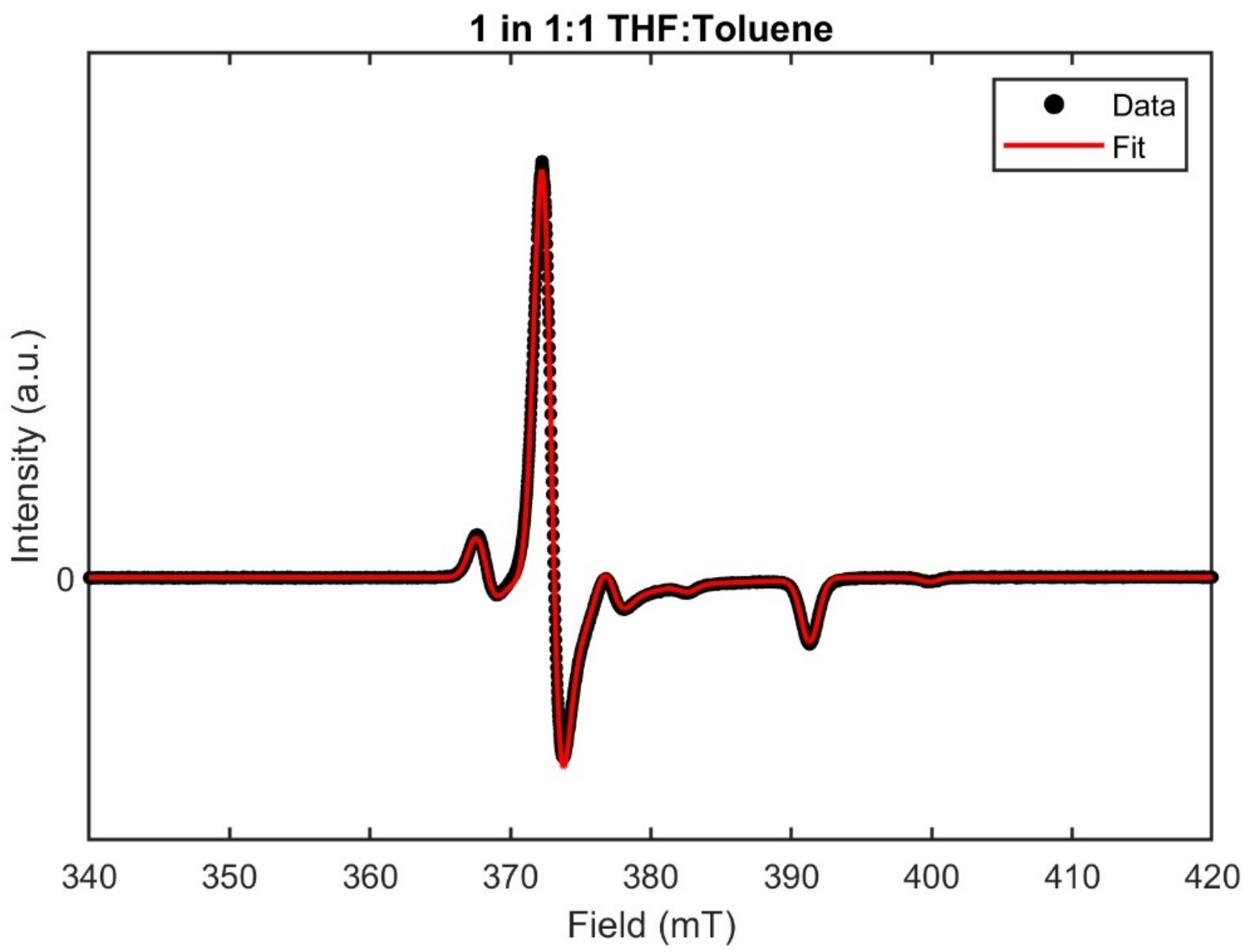

Figure S9: X-band (9.39 GHz) CW-EPR spectrum of **1** in 1:1 THF:toluene. Collected at 77 K with a modulation amplitude of 2 Gauss and a microwave power of 0.14 mW.

S3. Pulse Electron Paramagnetic Resonance (Pulse EPR)

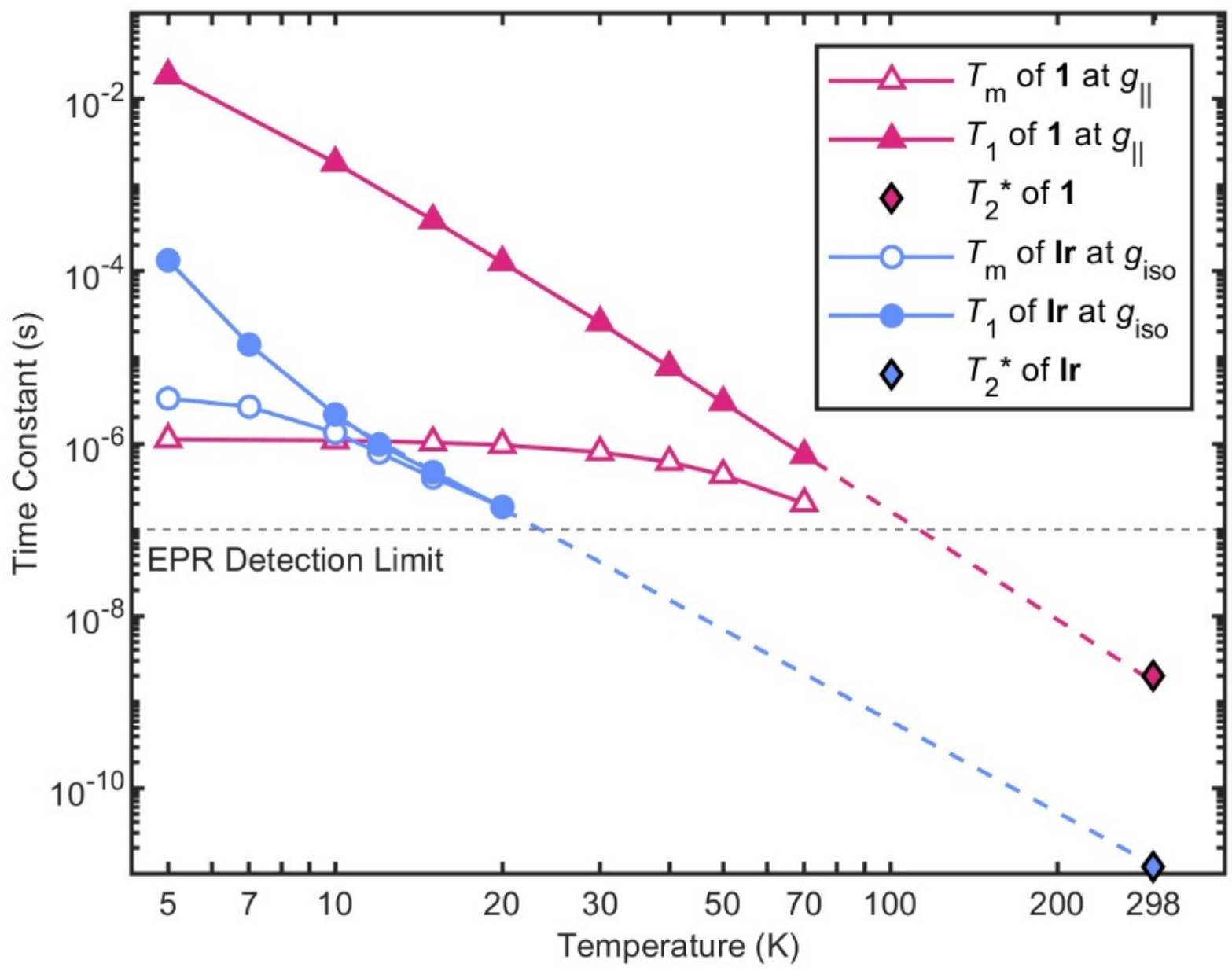

Figure S10: T_1 and T_m data for **1** at $g_{||}$ (11 mM in 2-MeTHF, pink) and K_2IrBr_6 (2 mM in 3:2 H_2O:glycerol, **Ir**, blue) at g_{iso}. Dashed line represents an extrapolation of a linear fit to T_1 between 30 and 70 K for **1** and between 10 and 20 K for **Ir**, while diamonds denote room-temperature TRFE/R data (longest component). Error bars are smaller than markers.

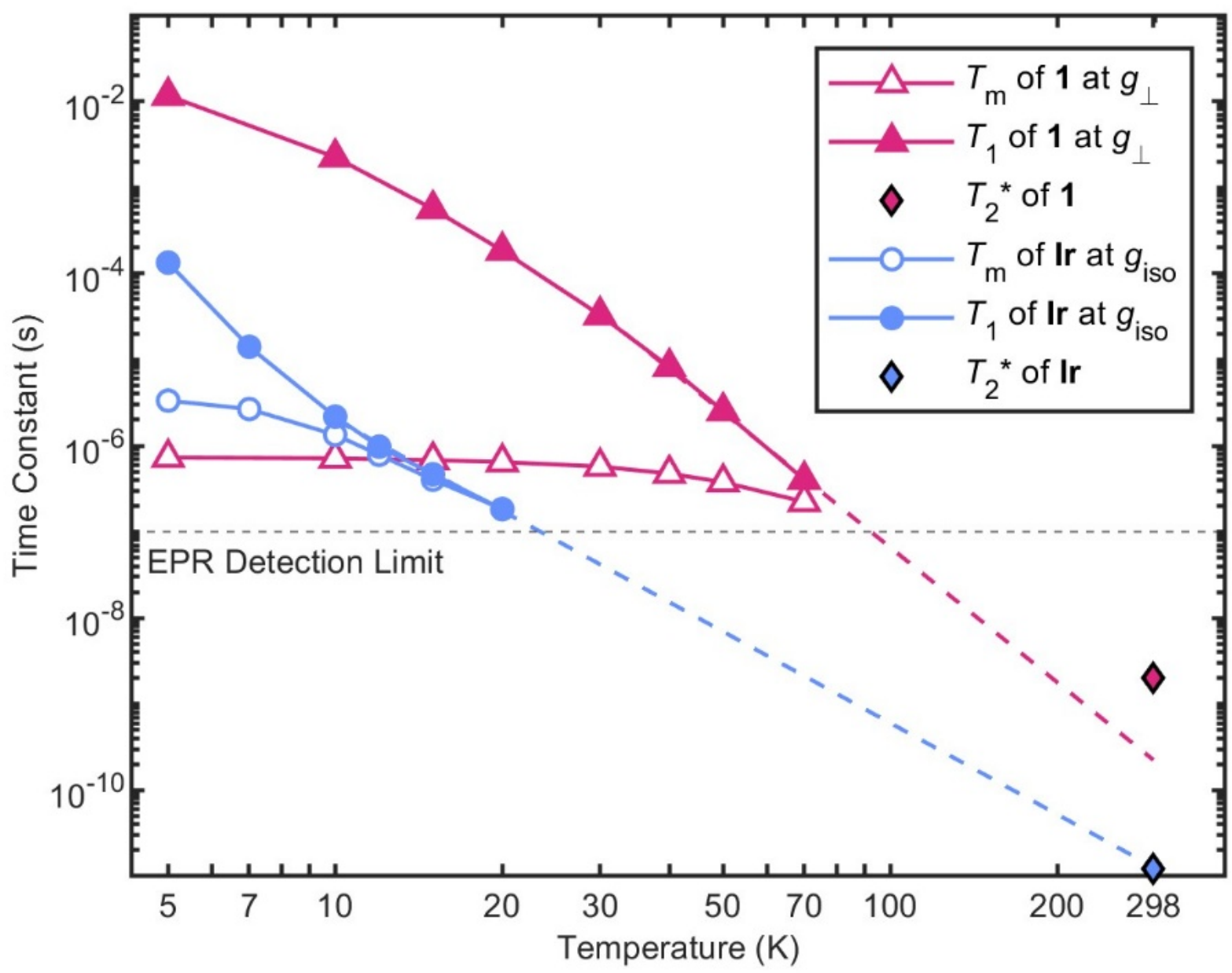

Figure S11: T_1 and T_m data for **1** at $g_{\perp}$ (11 mM in 2-MeTHF, pink) and K_2IrBr_6 (2 mM in 3:2 H_2O:glycerol, **Ir**, blue) at g_{iso}. Dashed line represents an extrapolation of a linear fit to T_1 between 30 and 70 K for **1** and between 10 and 20 K for **Ir**, while diamonds denote room-temperature TRFE/R data (longest component). Error bars are smaller than markers.

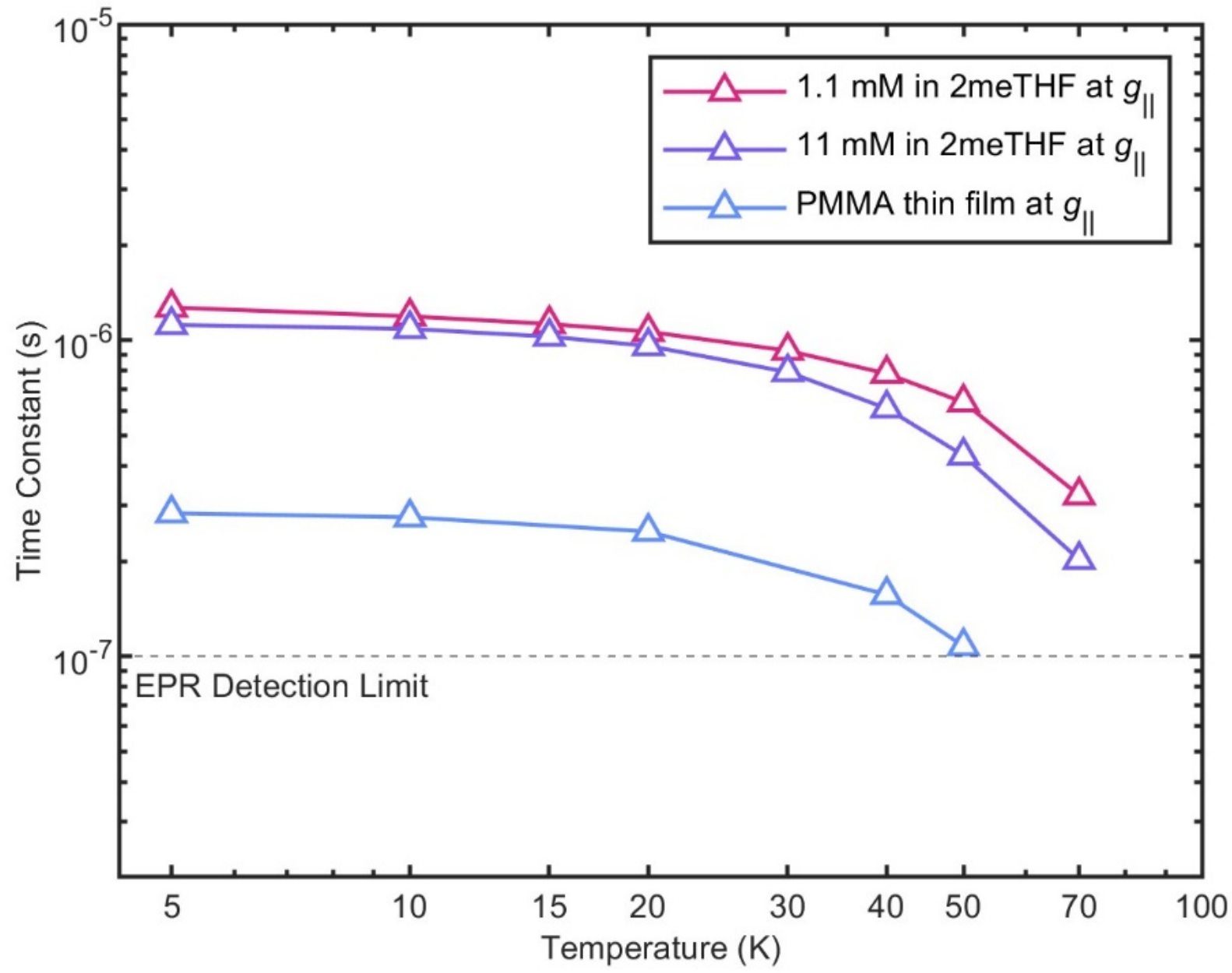

Figure S12: Pulse EPR T_m of **1** at $g_{||}$ for 1.1 mM in 2-MeTHF (pink), 11 mM in 2-MeTHF (purple), and PMMA thin film (blue). Rapid low-temperature decoherence in the PMMA film is likely due to the high concentration of spins. Error bars are smaller than markers.

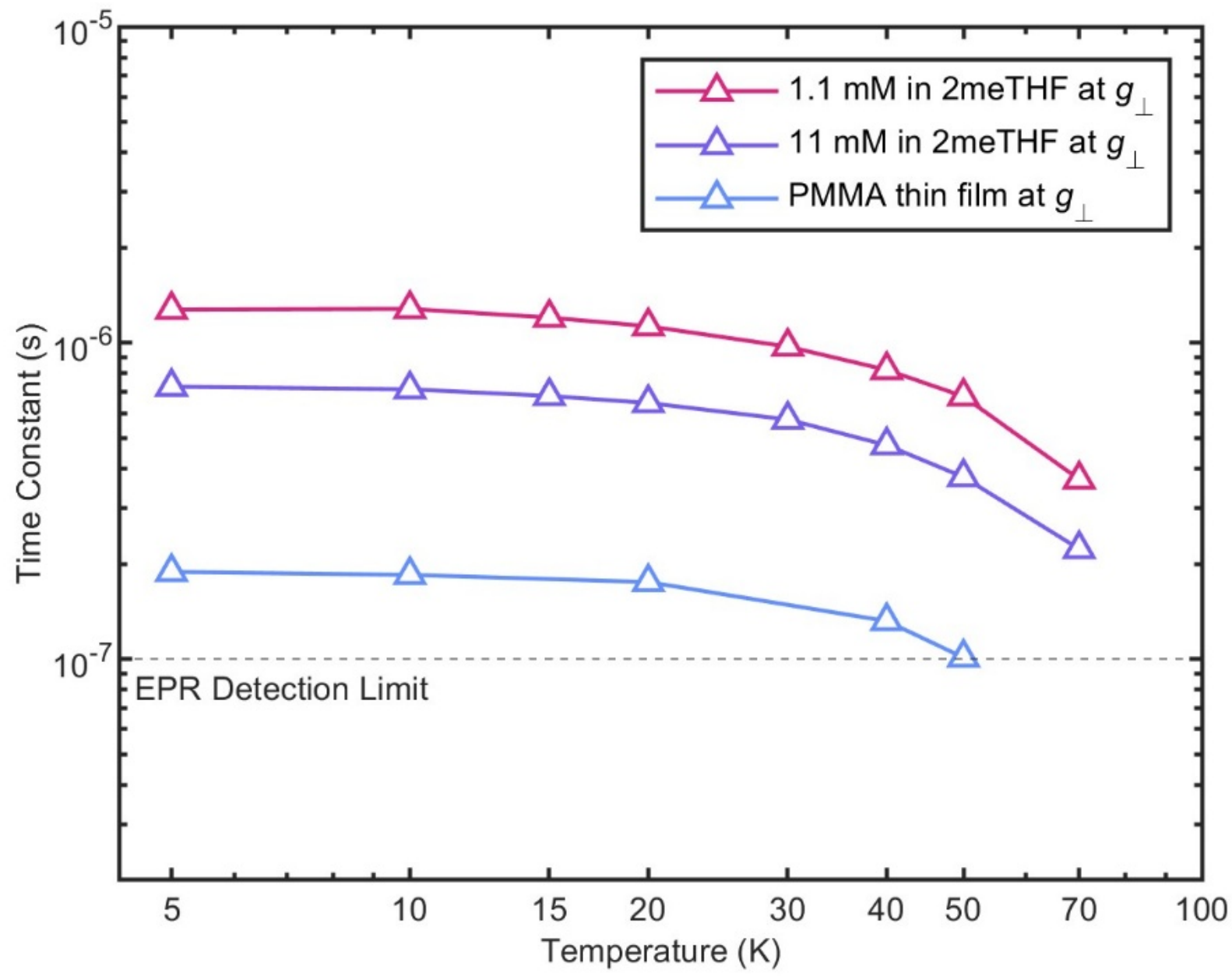

Figure S13: Pulse EPR T_m of **1** at $g_{\perp}$ for 1.1 mM in 2-MeTHF (pink), 11 mM in 2-MeTHF (purple), and PMMA thin film (blue). Rapid low-temperature decoherence in the PMMA film is likely due to the high concentration of spins. Error bars are smaller than markers.

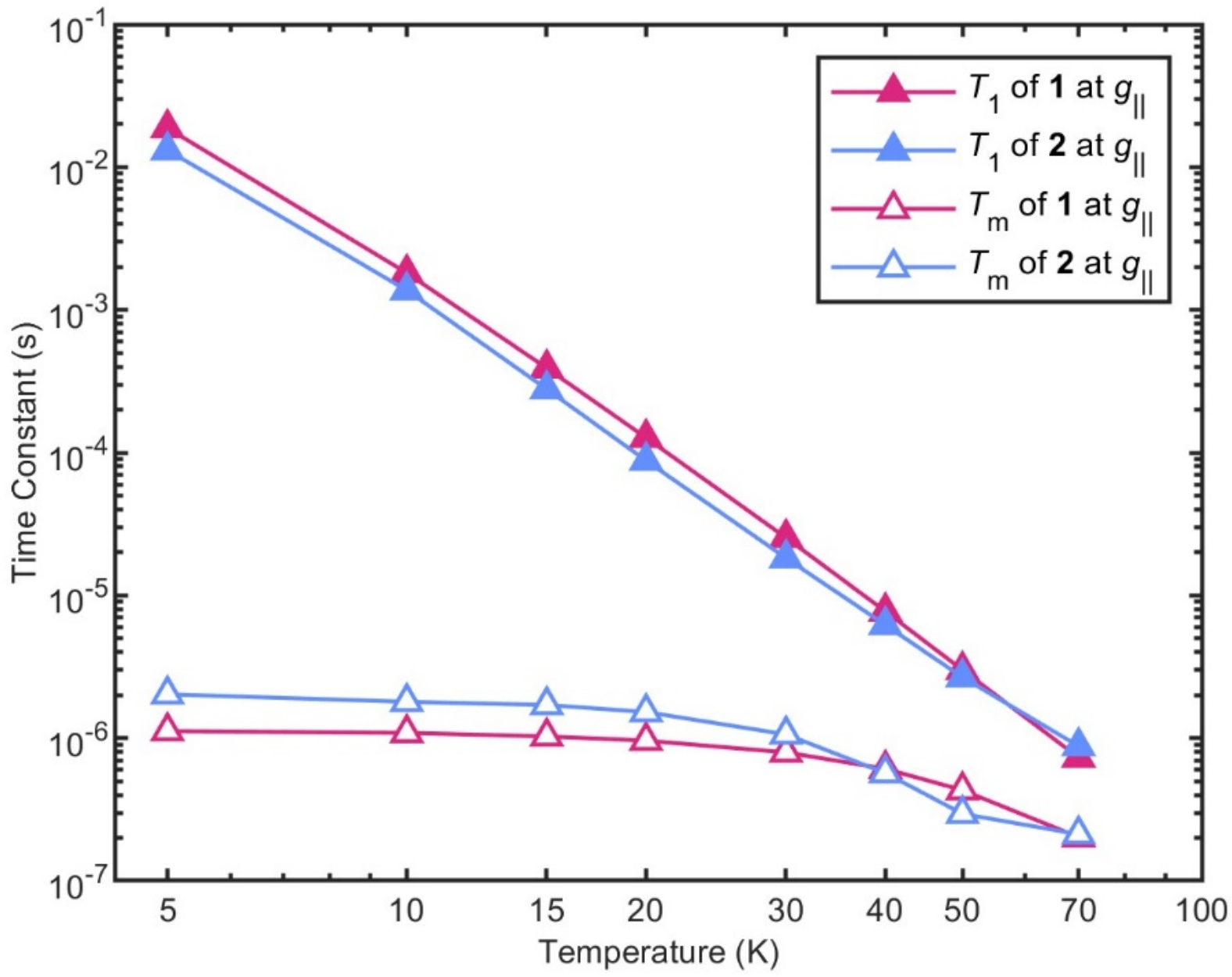

Figure S14: X-band pulse EPR (T_1 and T_m) data for **1** and **2** at $g_{||}$ (11 mM in 2-MeTHF).

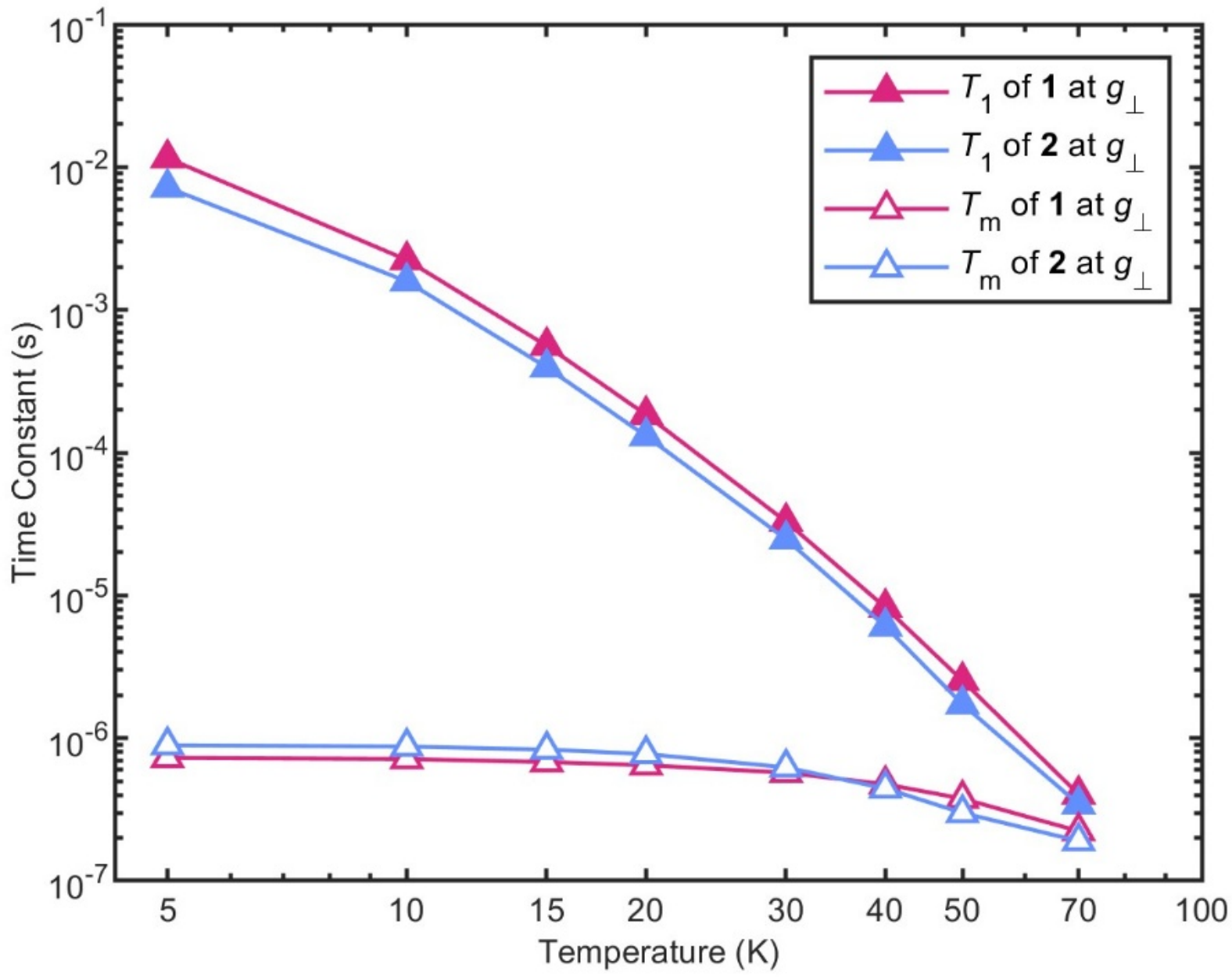

Figure S15: X-band pulse EPR (T_1 and T_m) data for **1** and **2** at $g_{\perp}$ (11 mM in 2-MeTHF).

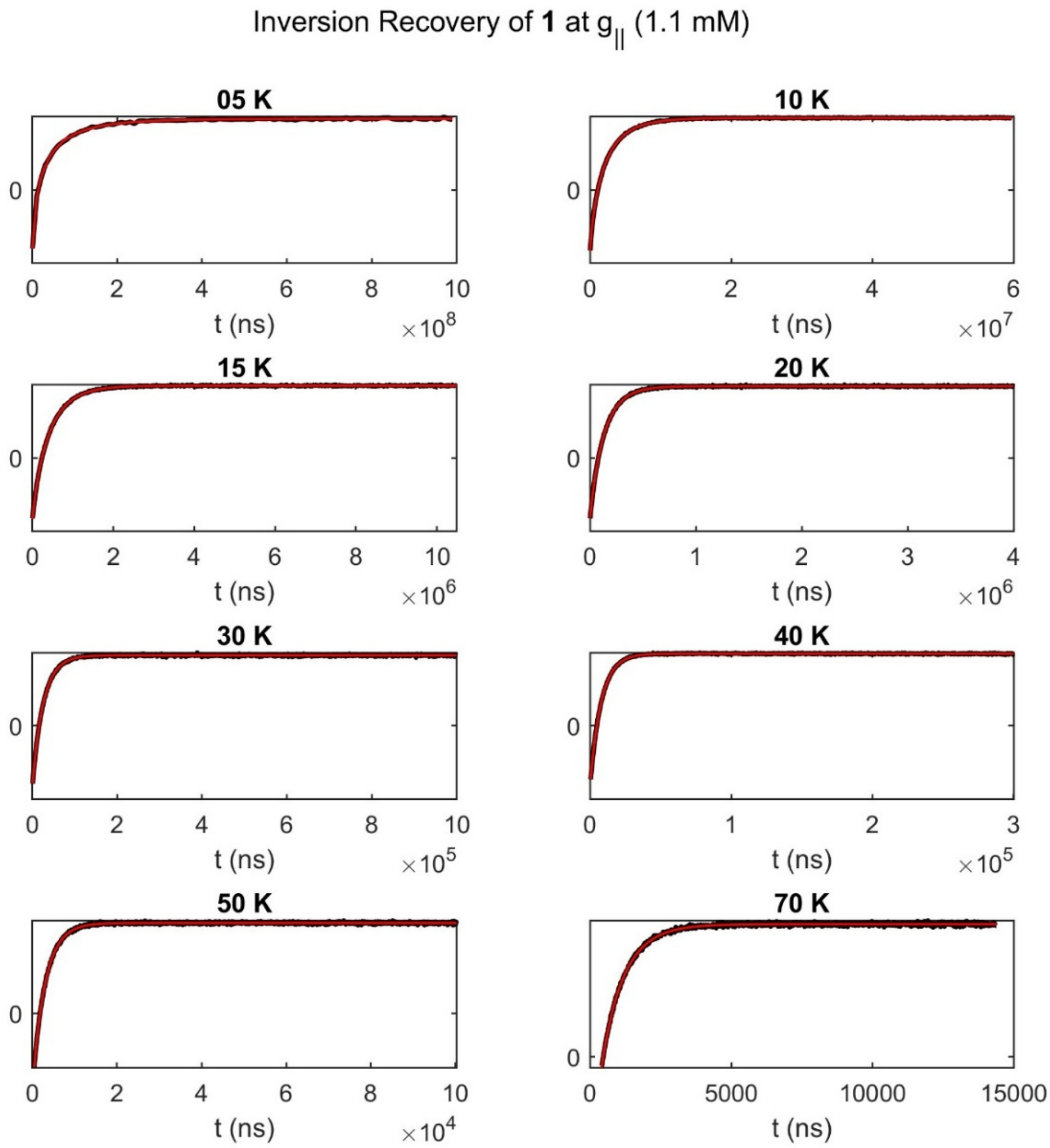

Figure S16: Inversion recovery of **1** (1.1 mM in 2-MeTHF) at $g_{||}$. 9.69 GHz, 4032 G

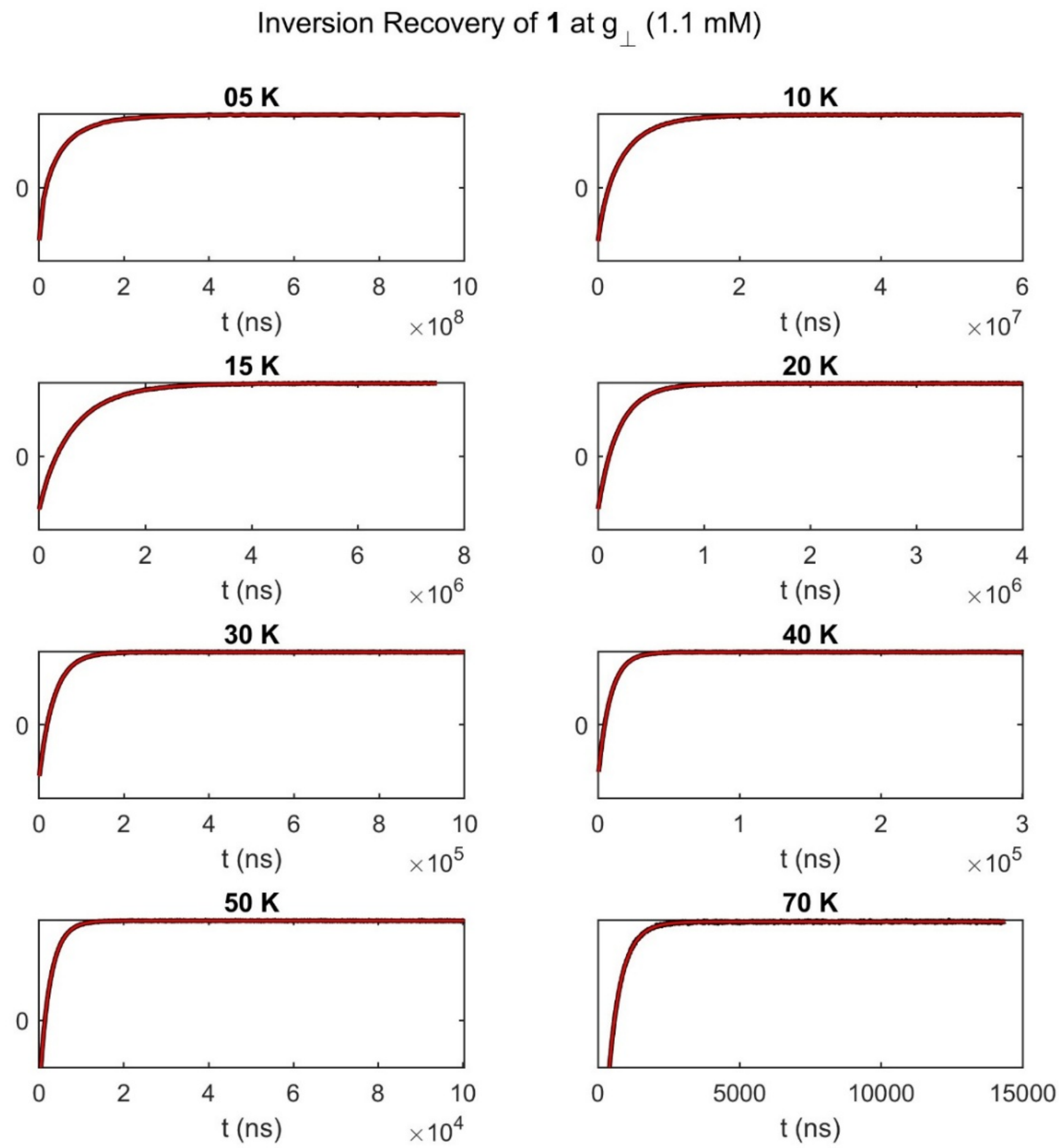

Figure S17: Inversion recovery of **1** (1.1 mM in 2-MeTHF) at $g_{\perp}$. 9.69 GHz, 3845 G

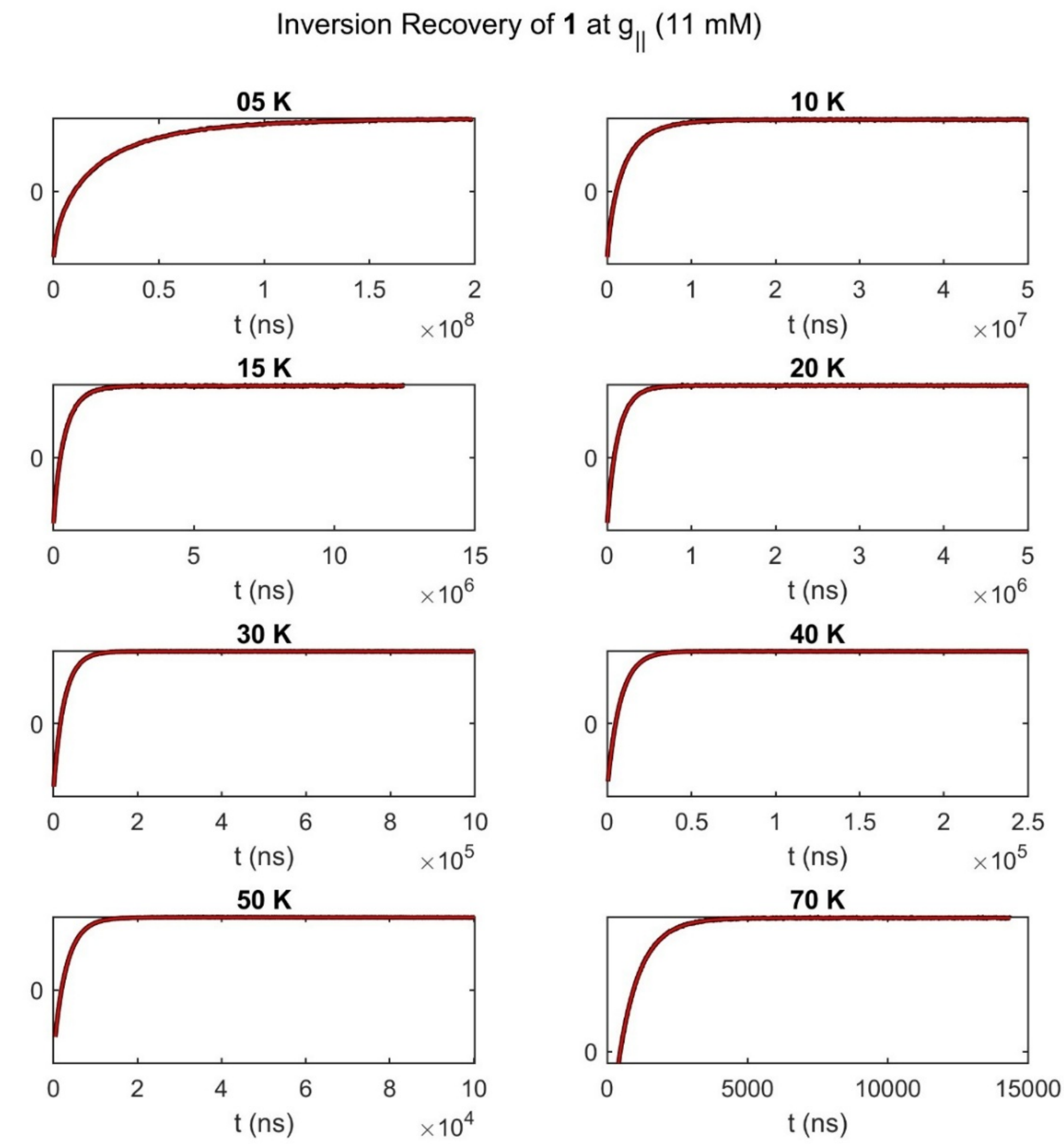

Figure S18: Inversion recovery of **1** (11 mM in 2-MeTHF) at $g_{||}$. 9.69 GHz, 4032 G

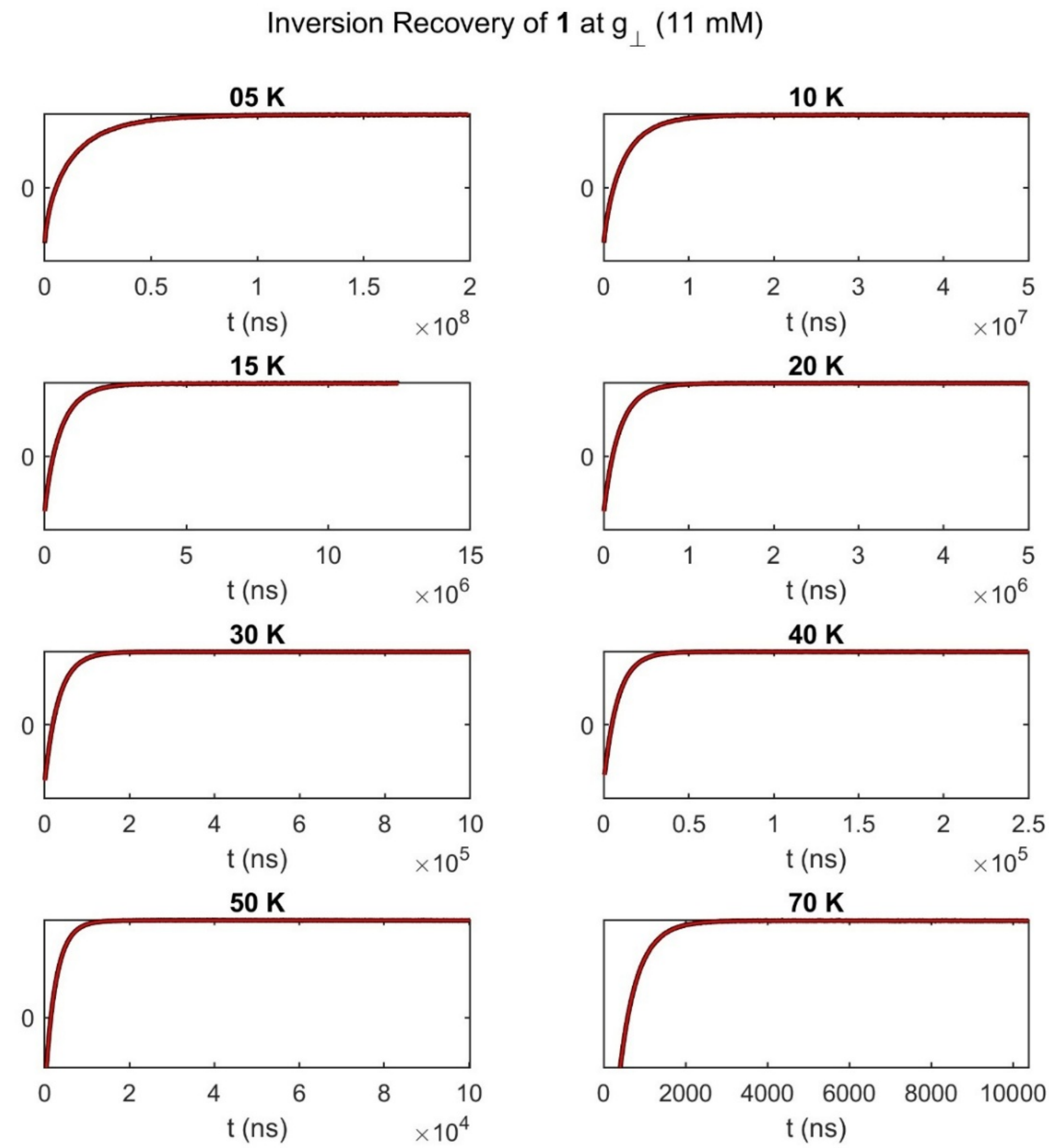

Figure S19: Inversion recovery of **1** (11 mM in 2-MeTHF) at $g_{\perp}$. 9.69 GHz, 3845 G

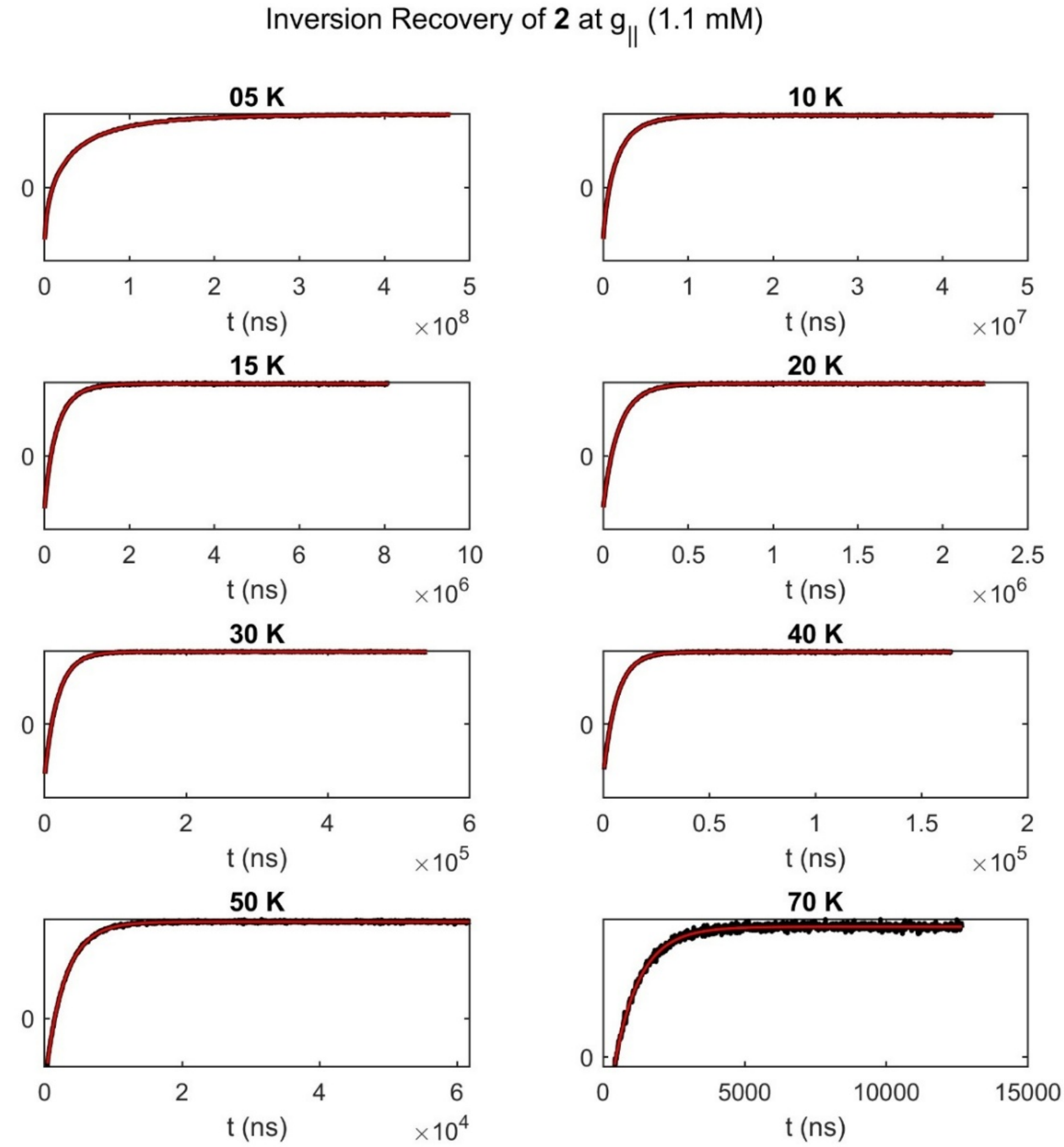

Figure S20: Inversion recovery of **2** (1.1 mM in 2-MeTHF) at $g_{||}$. 9.69 GHz, 4025 G

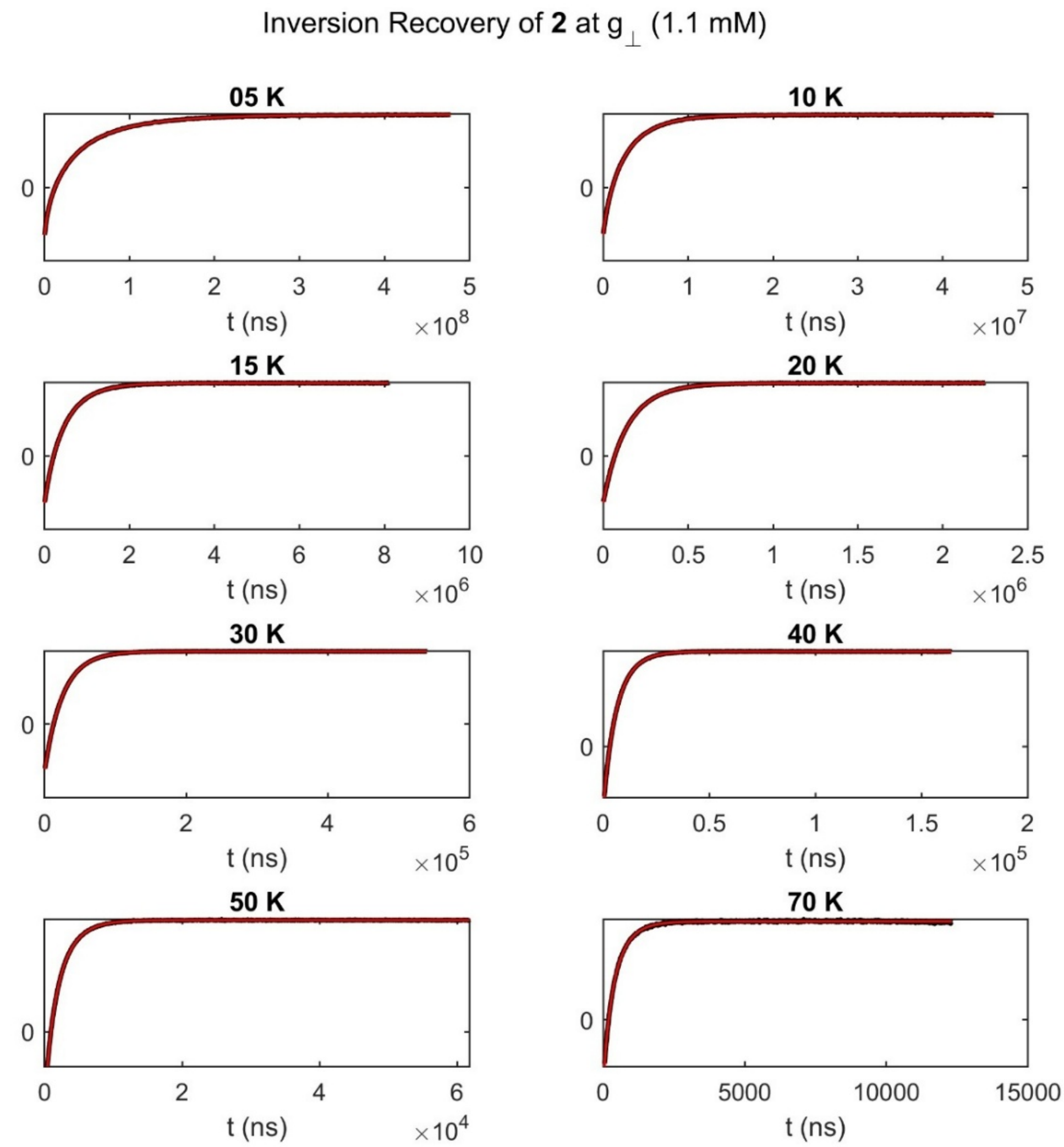

Figure S21: Inversion recovery of **2** (1.1 mM in 2-MeTHF) at $g_{\perp}$. 9.69 GHz, 3825 G

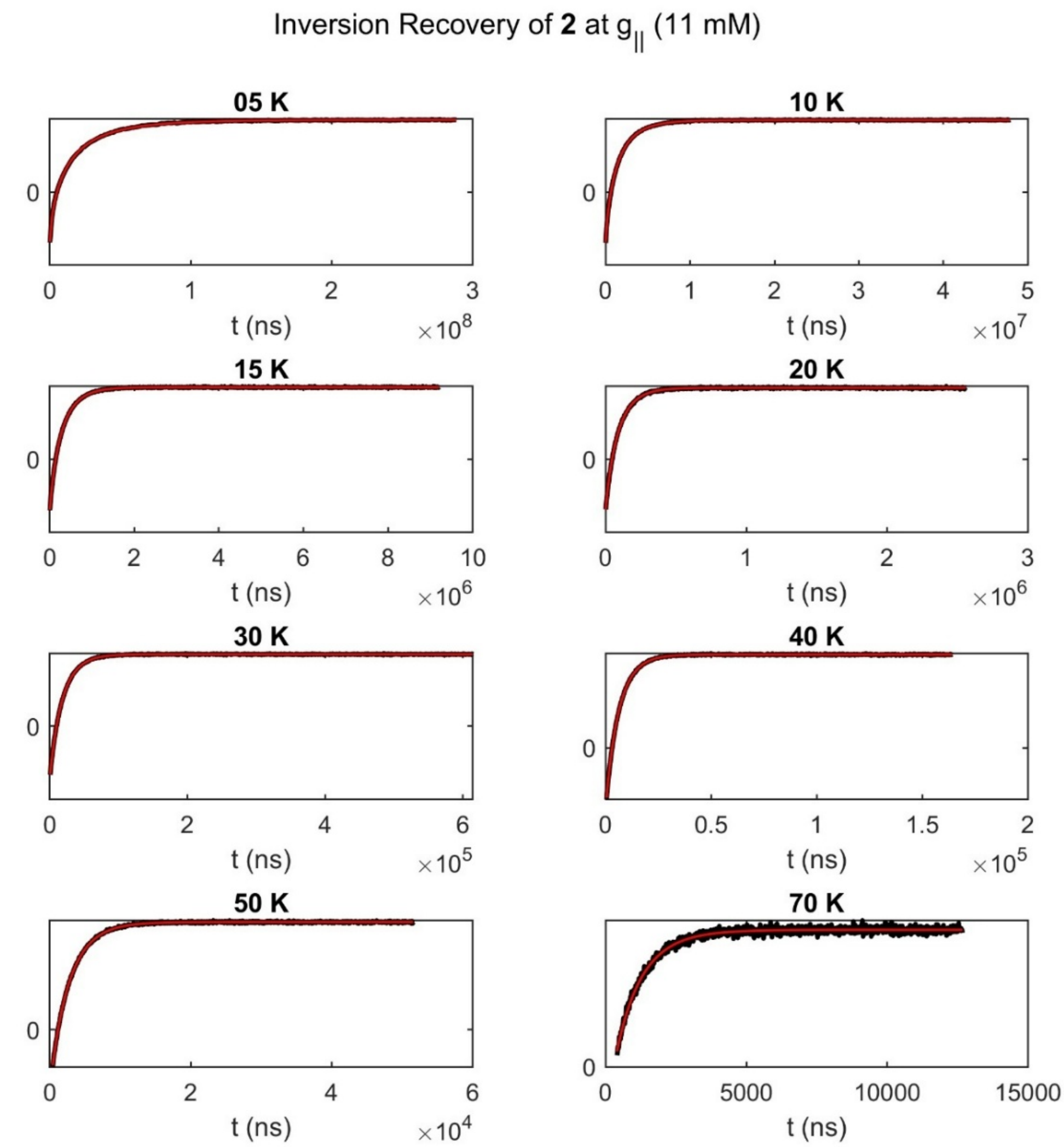

Figure S22: Inversion recovery of **2** (11 mM in 2-MeTHF) at $g_{||}$. 9.69 GHz, 4025 G

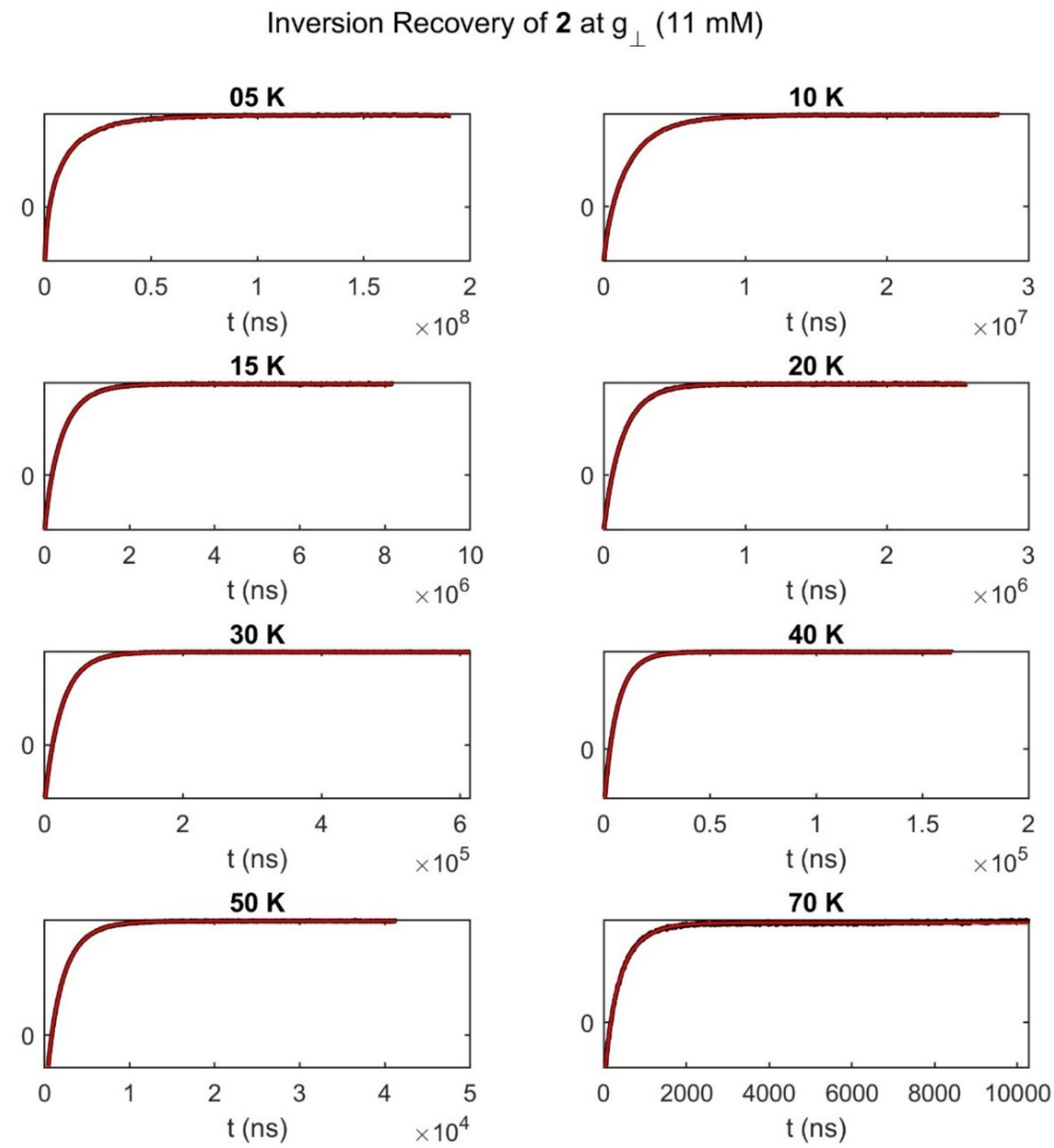

Figure S23: Inversion recovery of **2** (11 mM in 2-MeTHF) at $g_{\perp}$. 9.69 GHz, 3825 G

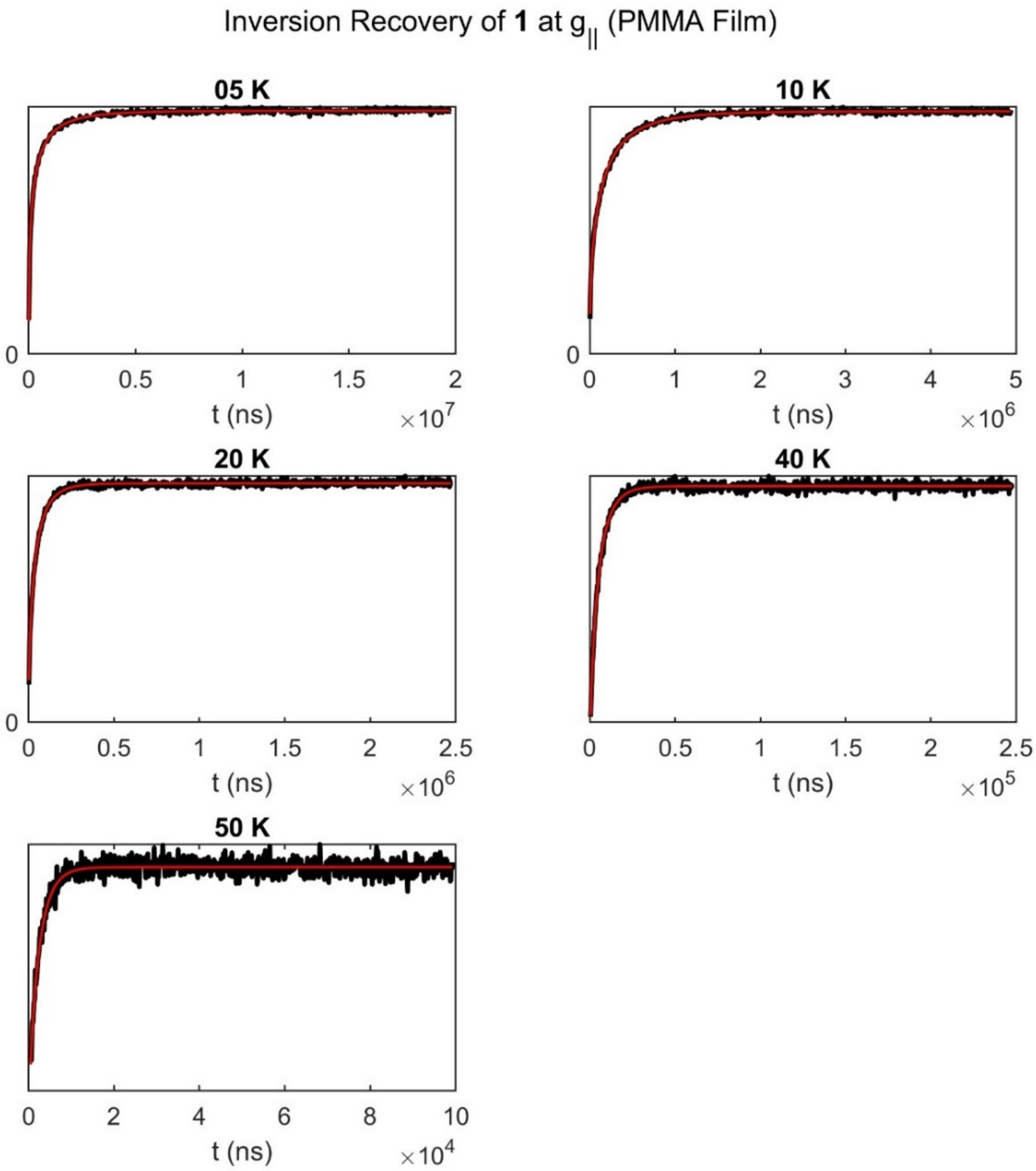

Figure S24: Inversion recovery of **1** (PMMA thin film) at $g_{||}$. 9.69 GHz, 4055 G

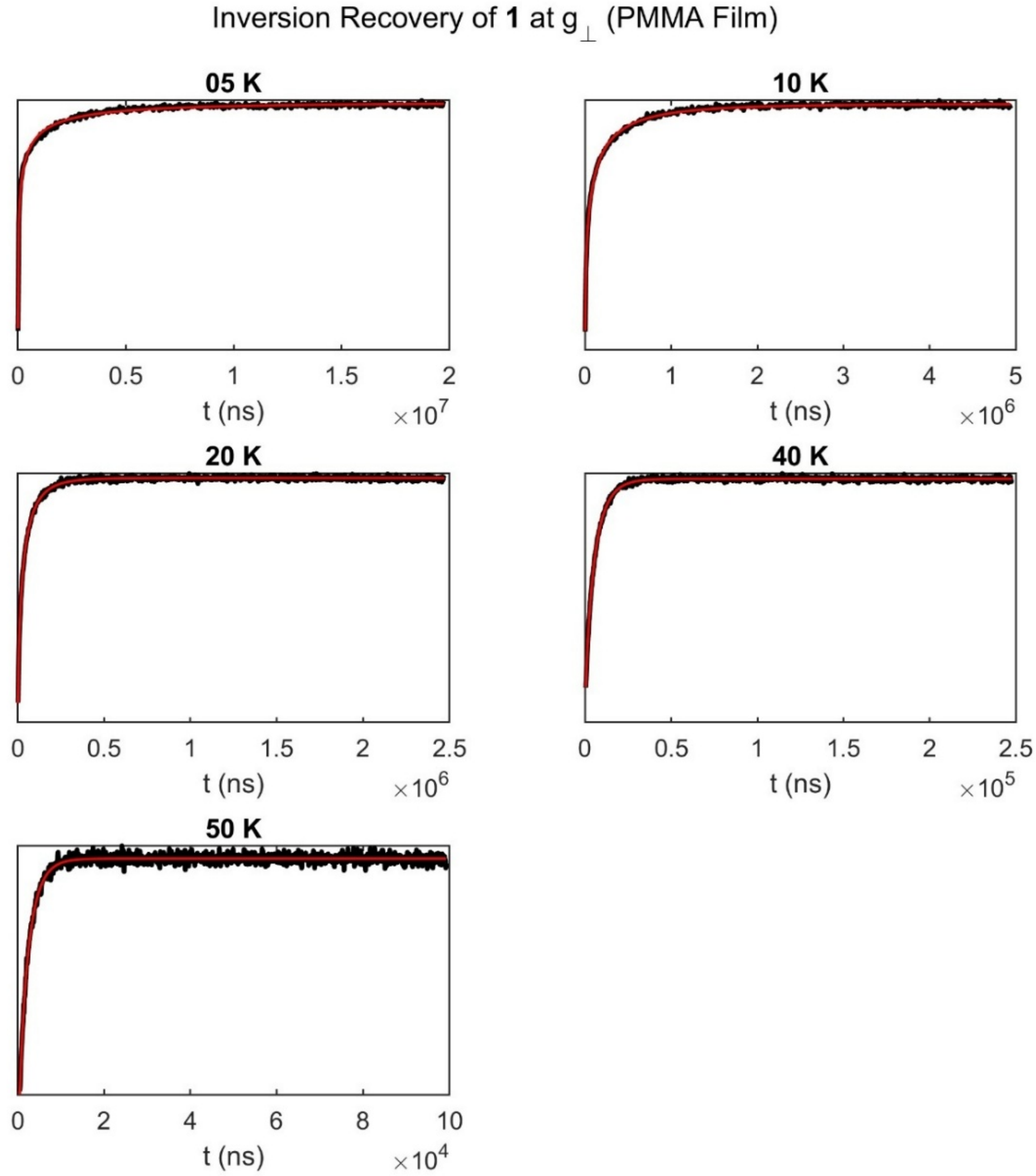

Figure S25: Inversion recovery of **1** (PMMA thin film) at $g_{\perp}$. 9.69 GHz, 3850 G

Table S2: Tabulated T_1 (inversion recovery) data for **1** in 2-MeTHF. All data in seconds. Numbers in parentheses indicate 95% confidence interval from the fit.

Temperature (K)	T_1 at $g_{\|\|}$, 1.1 mM	T_1 at $g_{\perp}$, 1.1 mM	T_1 at $g_{\|\|}$, 11 mM	T_1 at $g_{\perp}$, 11 mM
5	2.95 (7) E-02	3.76 (3) E-02	1.877 (12) E-02	1.157 (5) E-02
10	1.975 (15) E-03	3.051 (11) E-03	1.814 (10) E-03	2.237 (7) E-03
15	4.02 (2) E-04	6.168 (15) E-04	3.90 (2) E-04	5.60 (2) E-04
20	1.299 (8) E-04	1.922 (4) E-04	1.271 (5) E-04	1.843 (3) E-04
30	2.56 (2) E-05	3.465 (9) E-05	2.532 (6) E-05	3.286 (6) E-05
40	7.82 (5) E-06	8.45 (3) E-06	7.72 (2) E-06	8.223 (14) E-06
50	2.96 (4) E-06	2.465 (10) E-06	3.001 (8) E-06	2.528 (7) E-06
70	7.48 (22) E-07	4.02 (10) E-07	7.31 (8) E-07	4.04 (4) E-07

Table S3: Tabulated T_1 (inversion recovery) data for **2** in 2-MeTHF. All data in seconds. Numbers in parentheses indicate 95% confidence interval from the fit.

Temperature (K)	T_1 at $g_{\|\|}$, 1.1 mM	T_1 at $g_{\perp}$, 1.1 mM	T_1 at $g_{\|\|}$, 11 mM	T_1 at $g_{\perp}$, 11 mM
5	2.56 (2) E-02	3.182 (15) E-02	1.314 (13) E-02	7.20 (6) E-03
10	1.529 (10) E-03	2.372 (9) E-03	1.371 (9) E-03	1.584 (7) E-03
15	2.90 (2) E-04	4.489 (10) E-04	2.783 (13) E-04	3.952 (10) E-04
20	9.06 (4) E-05	1.388 (2) E-04	8.79 (6) E-05	1.306 (4) E-04
30	1.863 (8) E-05	2.558 (3) E-05	1.824 (8) E-05	2.473 (4) E-05
40	6.37 (3) E-06	6.269 (14) E-06	6.20 (3) E-06	6.061 (14) E-06
50	2.79 (3) E-06	1.890 (9) E-06	2.67 (2) E-06	1.728 (9) E-06
70	8.8 (4) E-07	3.85 (4) E-07	8.8 (6) E-07	3.44 (5) E-07

Table S4: Tabulated T_1 (inversion recovery) data for **1** in PMMA thin film. All data in seconds. Numbers in parentheses indicate 95% confidence interval from the fit.

Temperature (K)	T_1 at $g_{\|\|}$	T_1 at $g_{\perp}$
5	2.84 (4) E-07	1.89 (2) E-07
10	2.75 (4) E-07	1.85 (2) E-07
20	2.48 (4) E-07	1.75 (2) E-07
40	1.57 (3) E-07	1.319 (18) E-07
50	1.08 (3) E-07	1.012 (17) E-07

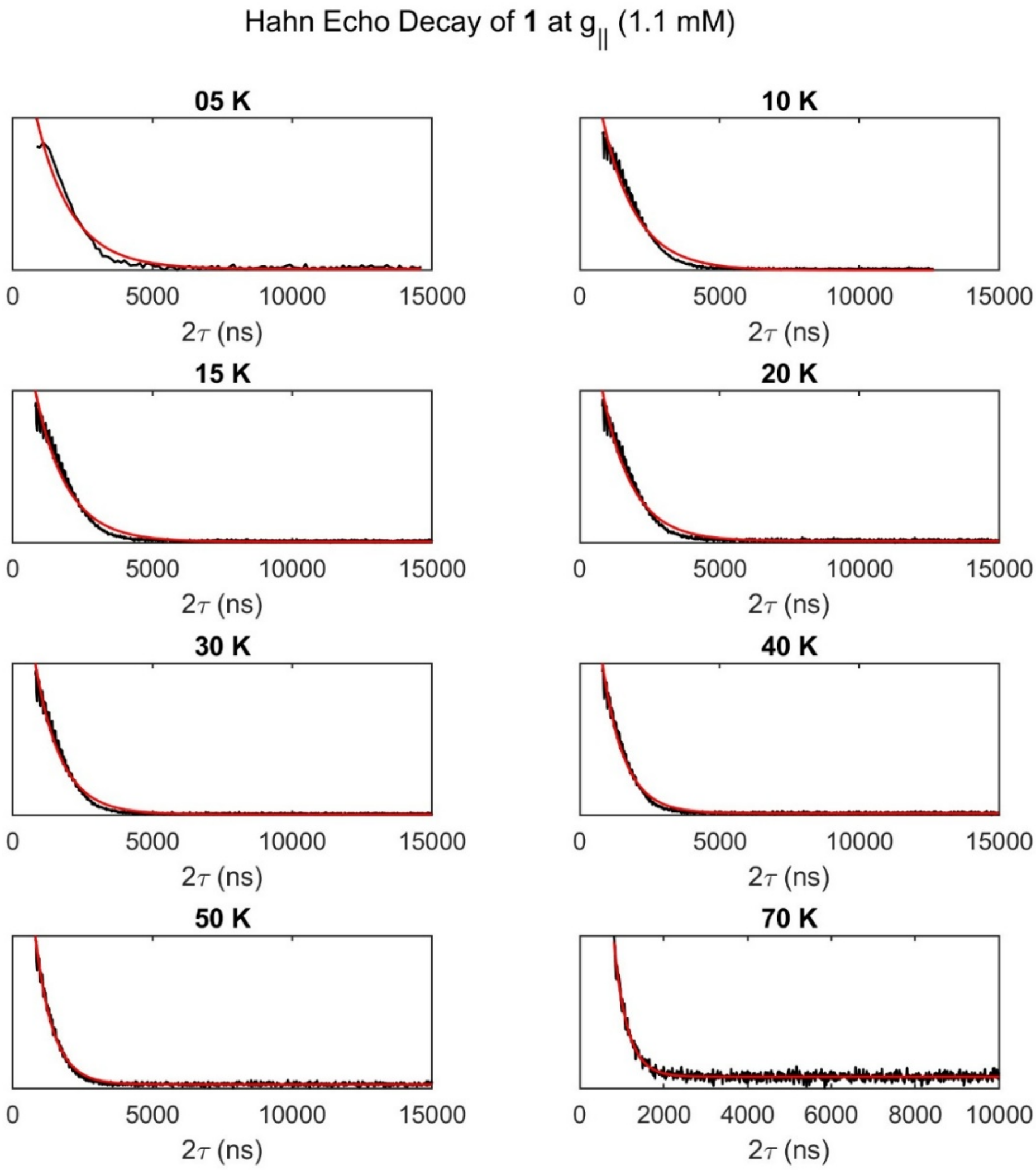

Figure S26: Hahn echo decay of **1** (1.1 mM in 2-MeTHF) at $g_{||}$. 9.69 GHz, 4032 G

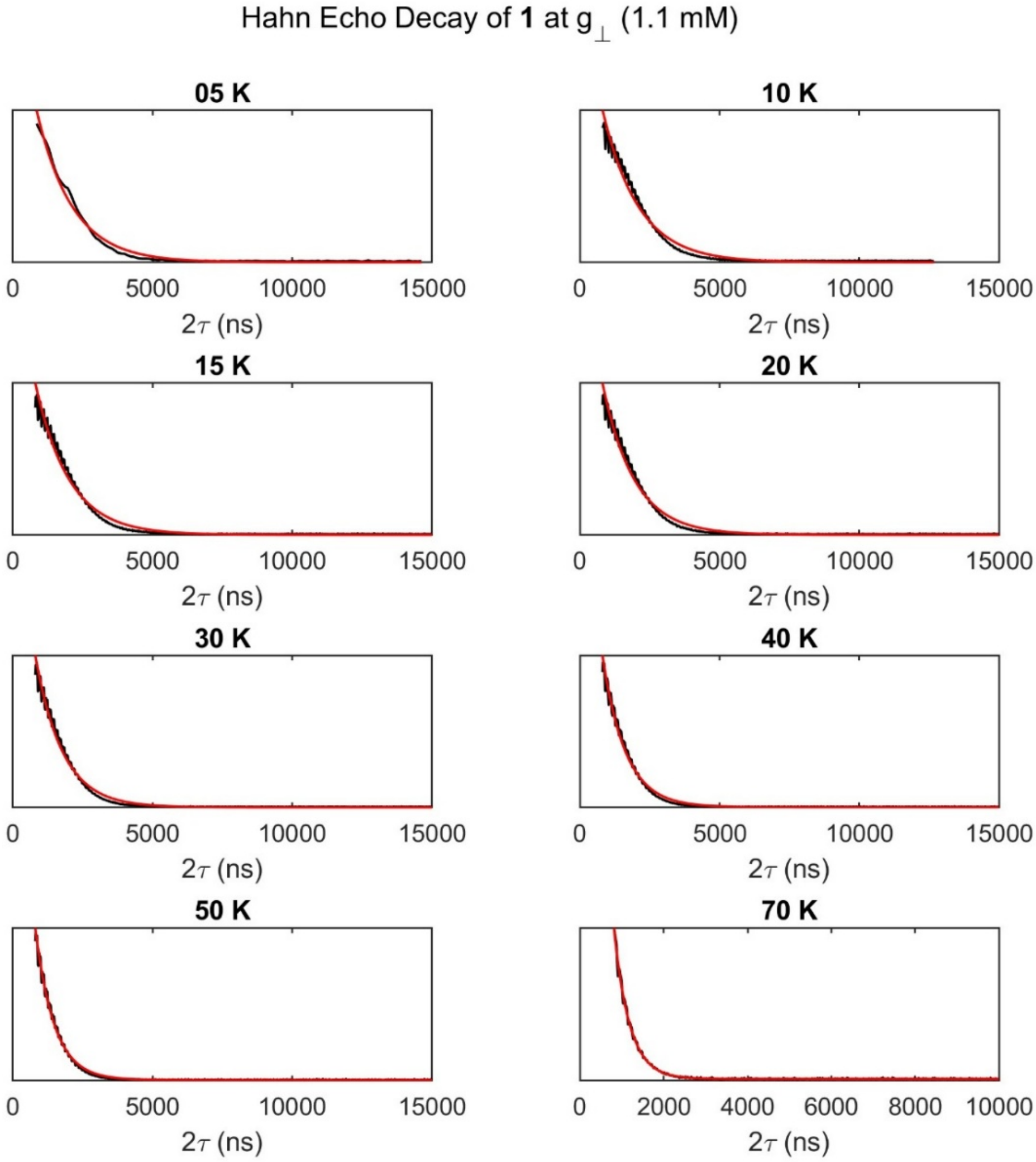

Figure S27: Hahn echo decay of **1** (1.1 mM in 2-MeTHF) at $g_{\perp}$. 9.69 GHz, 3845 G

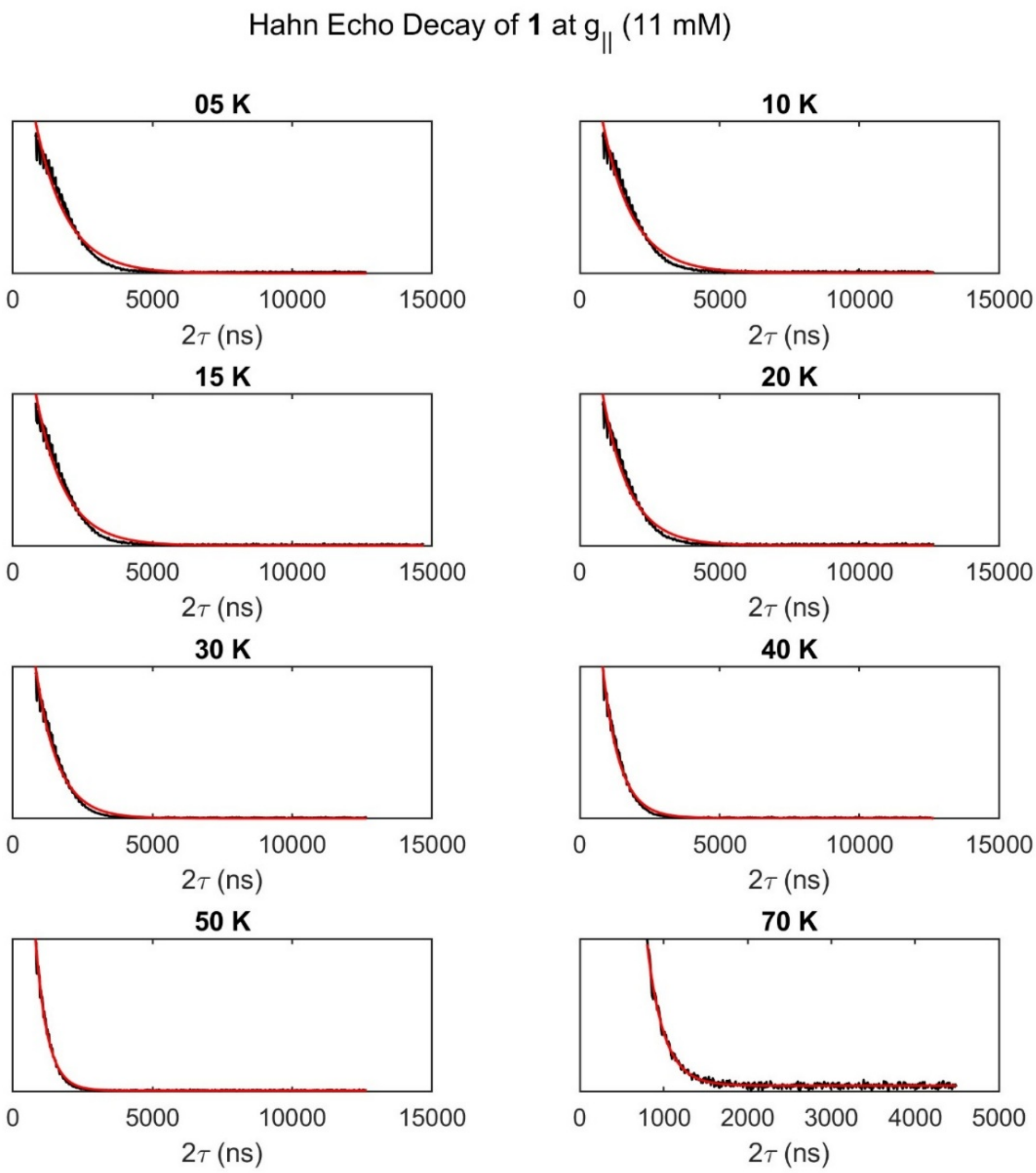

Figure S28: Hahn echo decay of **1** (11 mM in 2-MeTHF) at $g_{||}$**.** 9.69 GHz, 4032 G

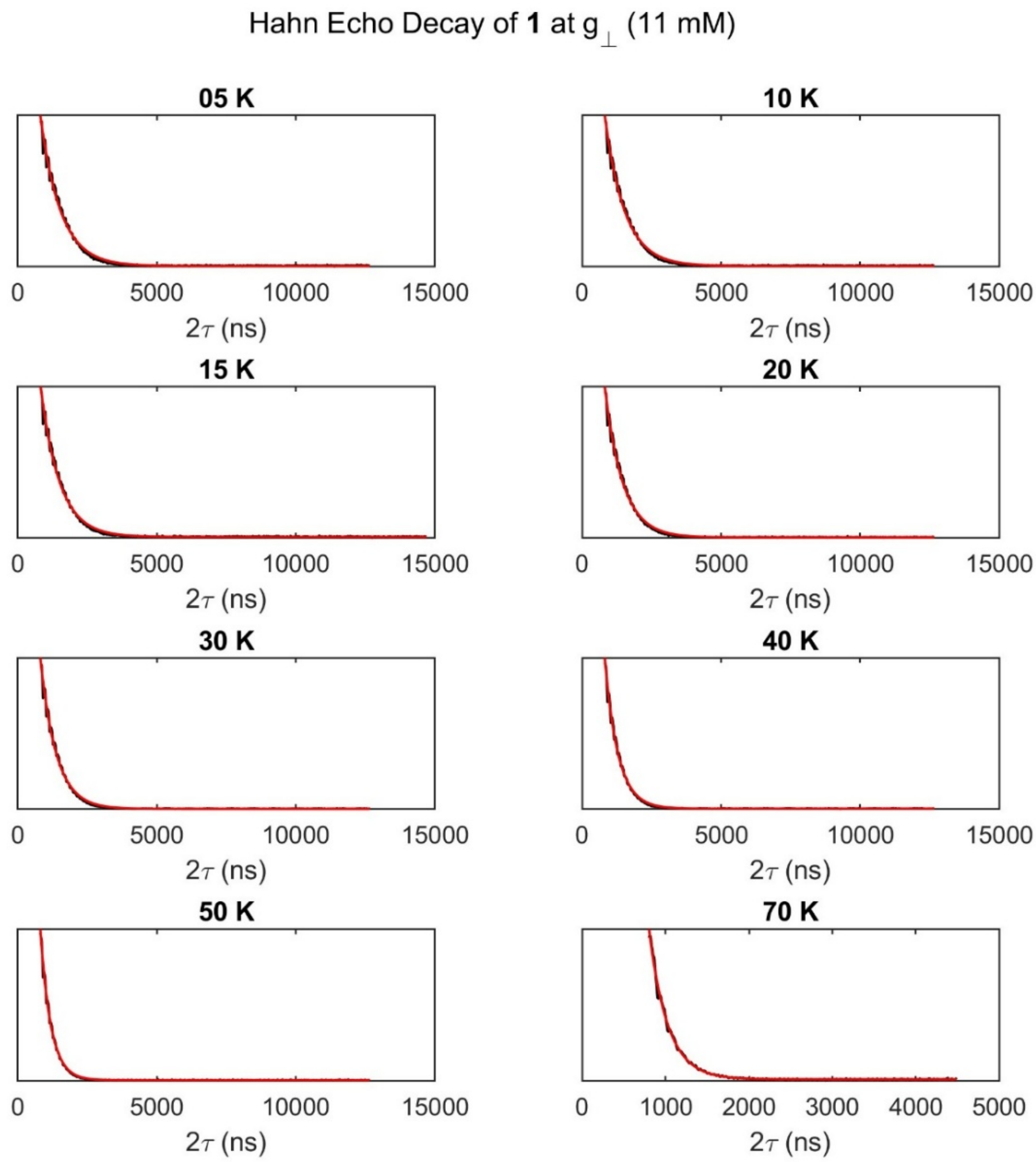

Figure S29: Hahn echo decay of **1** (11 mM in 2-MeTHF) at $g_{\perp}$. 9.69 GHz, 3845 G

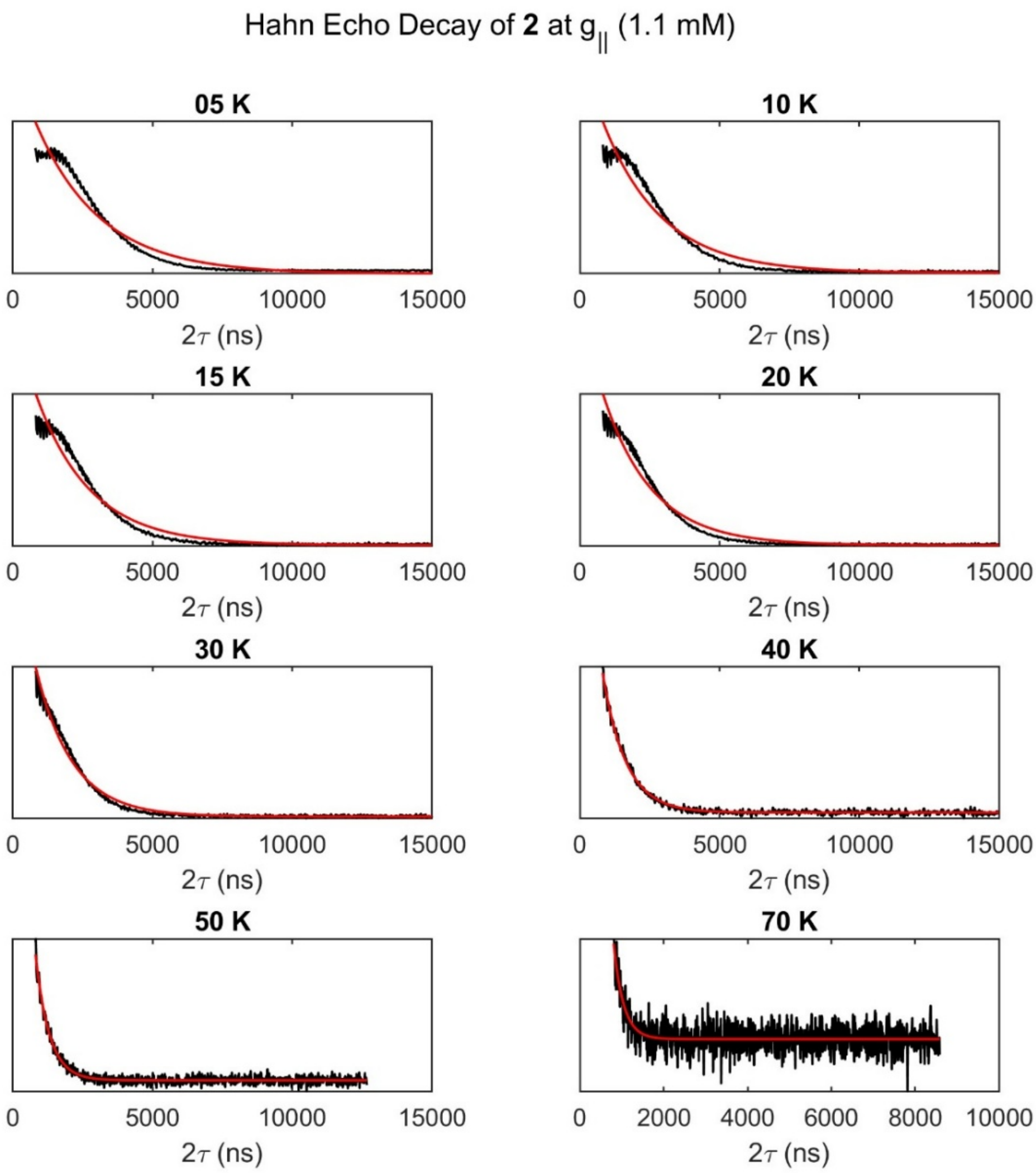

Figure S30: Hahn echo decay of **2** (1.1 mM in 2-MeTHF) at $g_{||}$. 9.69 GHz, 4025 G

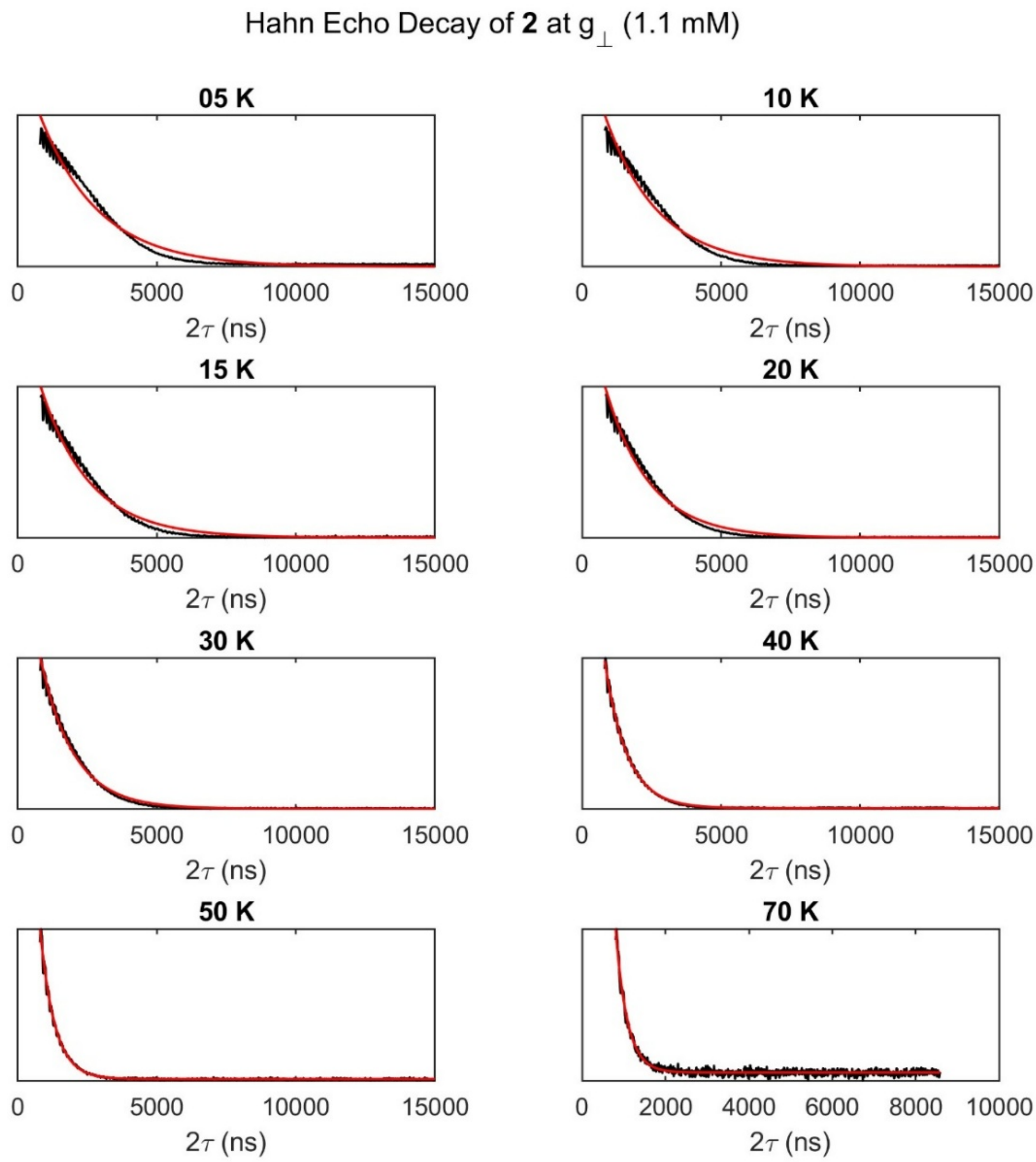

Figure S31: Hahn echo decay of **2** (1.1 mM in 2-MeTHF) at $g_{\perp}$. 9.69 GHz, 3825 G

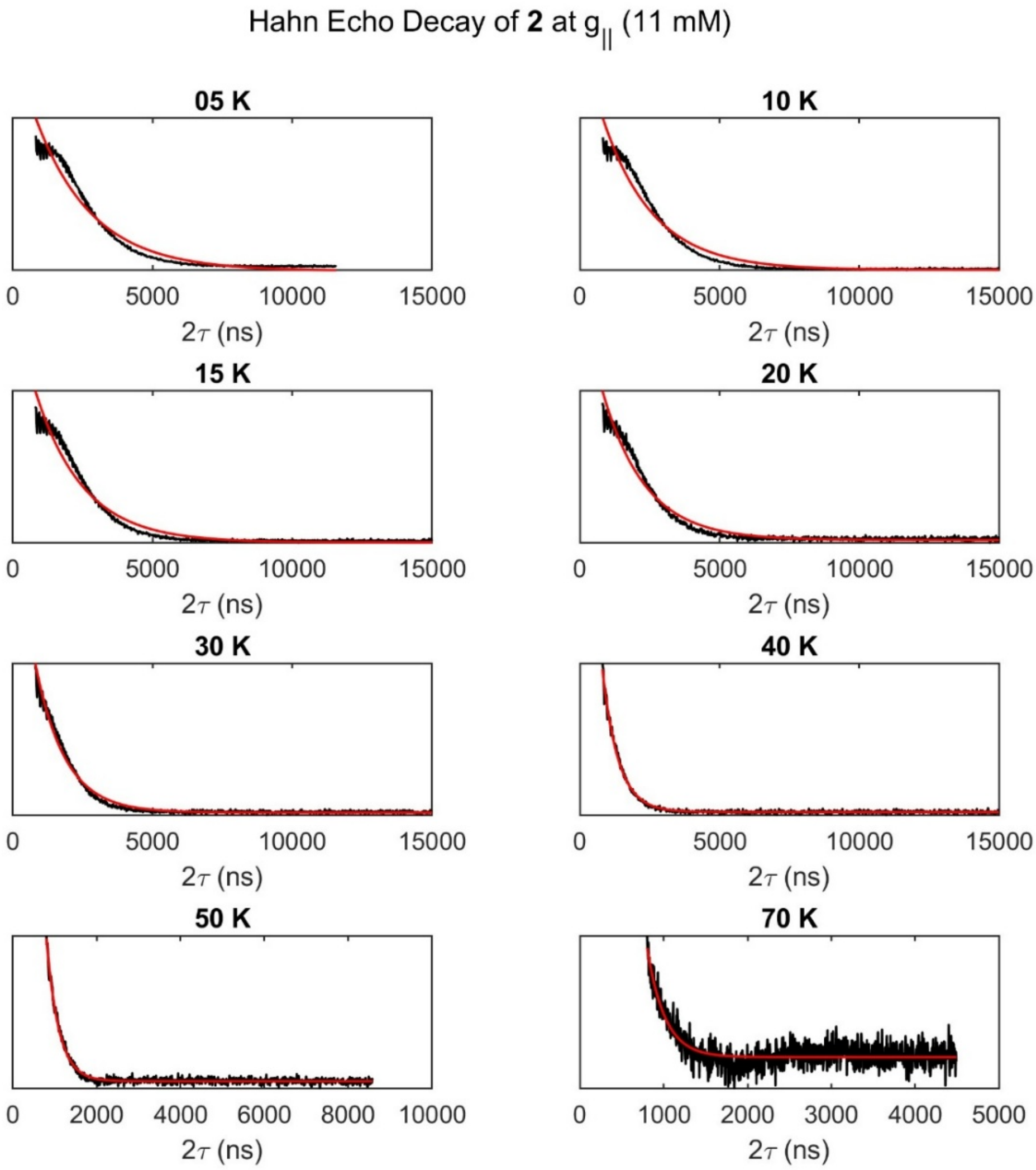

Figure S32: Hahn echo decay of **2** (11 mM in 2-MeTHF) at $g_{||}$. 9.69 GHz, 4025 G

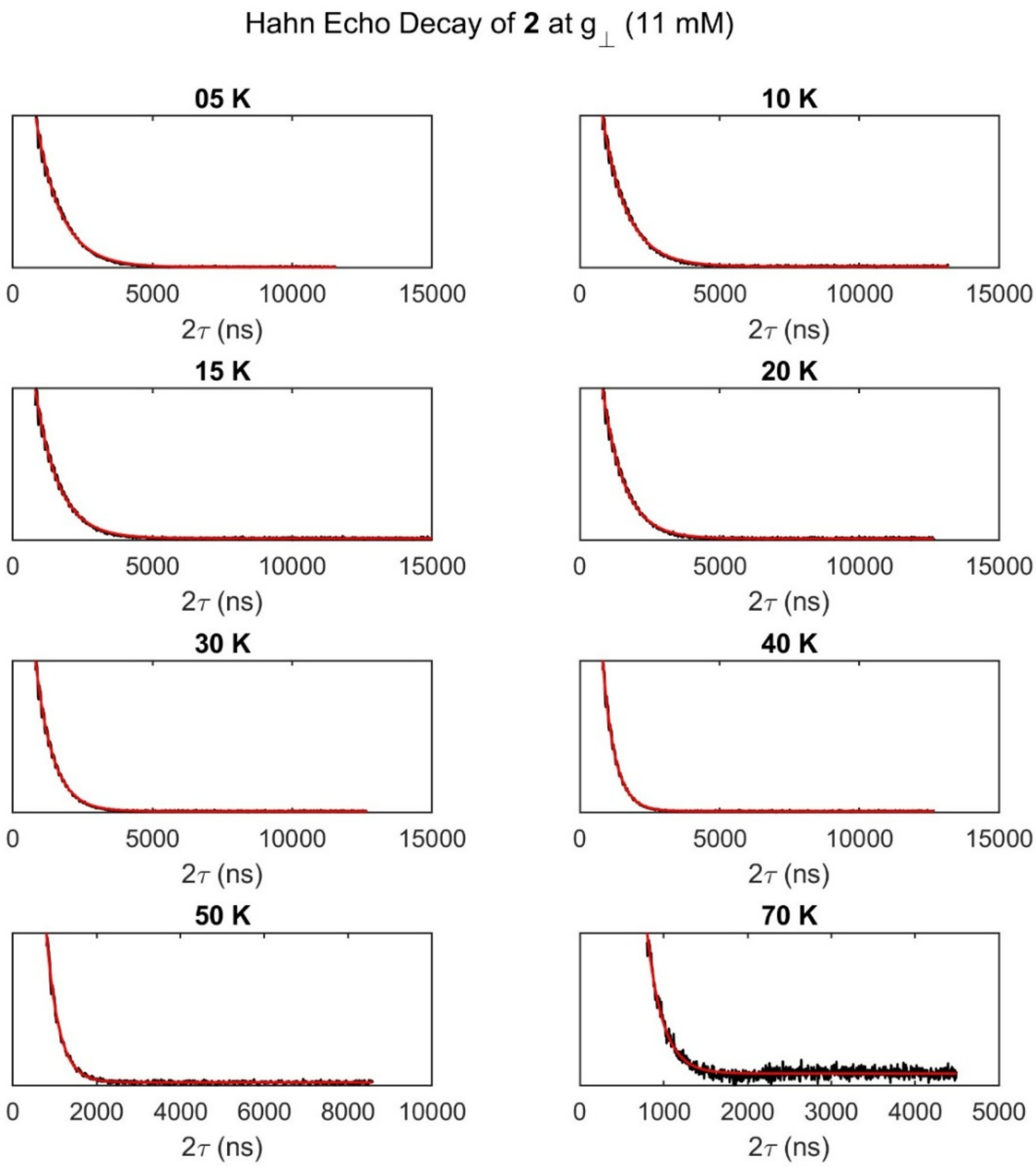

Figure S33: Hahn echo decay of **2** (11 mM in 2-MeTHF) at $g_{\perp}$. 9.69 GHz, 3825 G

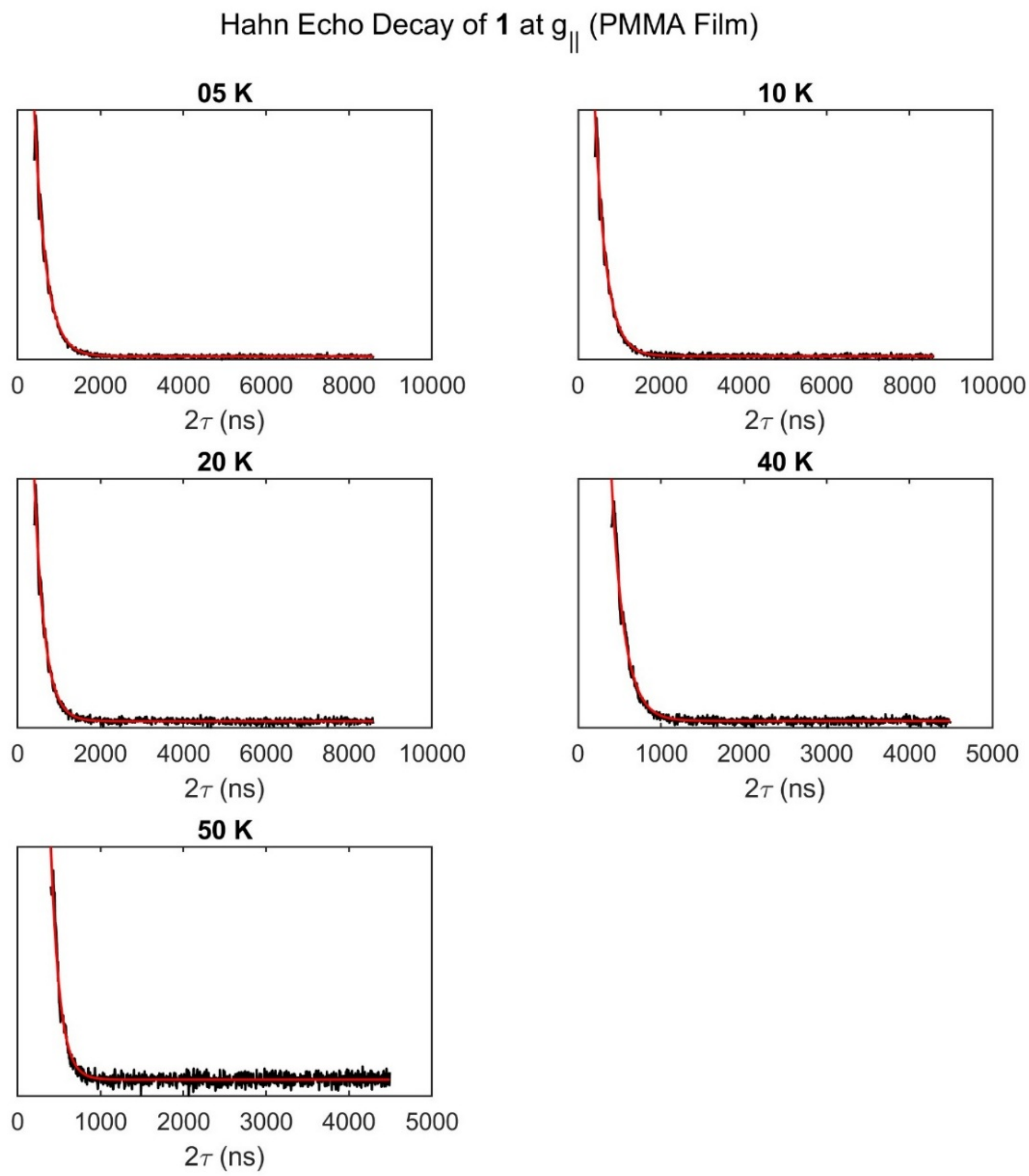

Figure S34: Hahn echo decay of **1** (PMMA thin film) at $g_{||}$. 9.69 GHz, 4055 G

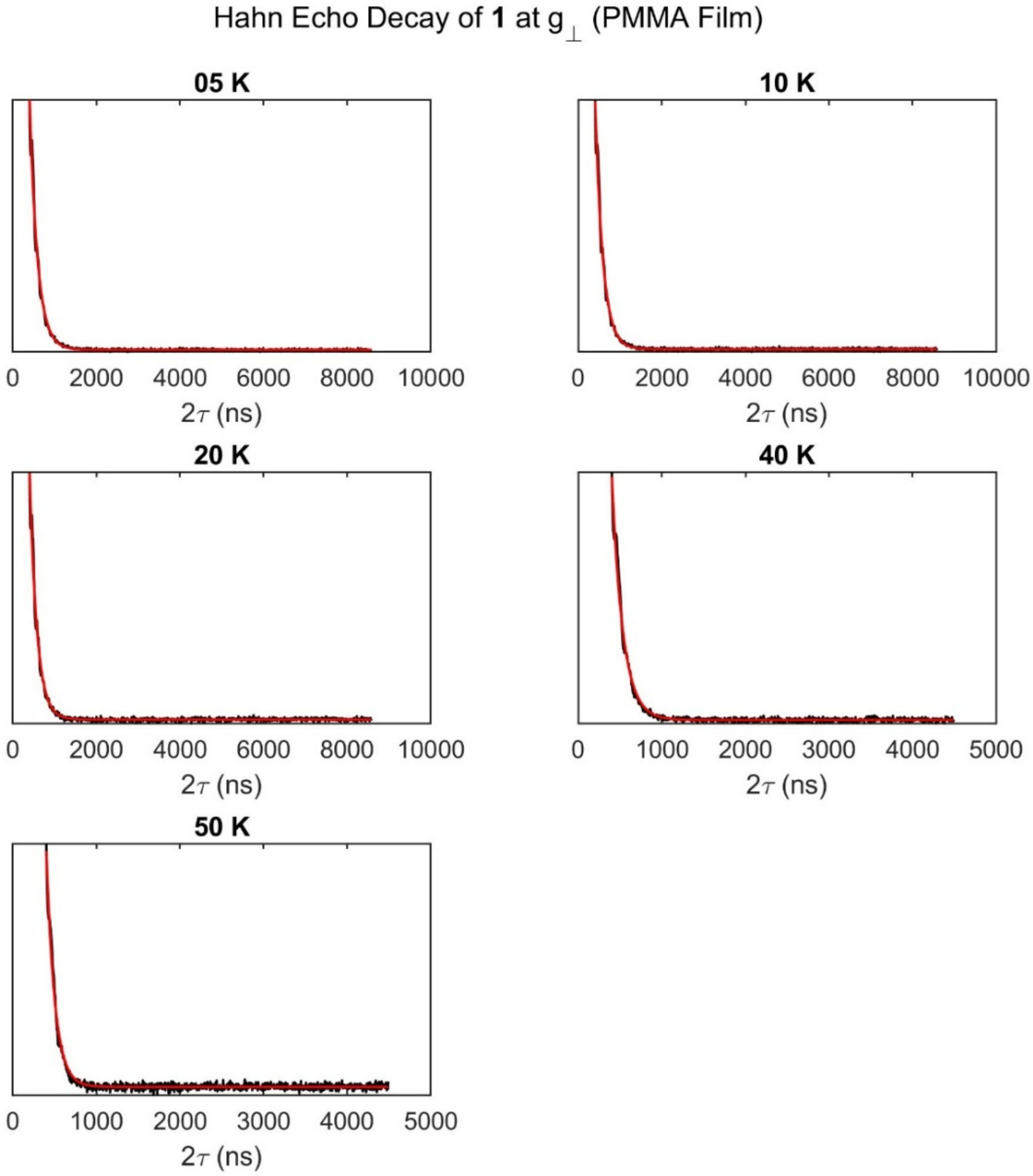

Figure S35: Hahn echo decay of **1** (PMMA thin film) at $g_{\perp}$. 9.69 GHz, 3850 G

Table S5: Tabulated T_m (Hahn echo decay) data for **1** in 2-MeTHF. All data in seconds. Numbers in parentheses indicate 95% confidence interval from the fit.

Temperature (K)	T_m at $g_{\|\|}$, 1.1 mM	T_m at $g_{\perp}$, 1.1 mM	T_m at $g_{\|\|}$, 11 mM	T_m at $g_{\perp}$, 11 mM
5	1.27 (8) E-06	1.27 (5) E-06	1.12 (3) E-06	7.27 (10) E-07
10	1.19 (3) E-06	1.28 (3) E-06	1.09 (3) E-06	7.13 (10) E-07
15	1.13 (2) E-06	1.20 (2) E-06	1.03 (2) E-06	6.79 (9) E-07
20	1.06 (2) E-06	1.13 (2) E-06	9.6 (2) E-07	6.46 (8) E-07
30	9.27 (13) E-07	9.73 (12) E-07	7.94 (15) E-07	5.72 (7) E-07
40	7.85 (10) E-07	8.21 (9) E-07	6.09 (10) E-07	4.75 (5) E-07
50	6.37 (8) E-07	6.77 (6) E-07	4.32 (6) E-07	3.77 (4) E-07
70	3.23 (9) E-07	3.70 (3) E-07	2.02 (4) E-07	2.23 (3) E-07

Table S6: Tabulated T_m (Hahn echo decay) data for **2** in 2-MeTHF. All data in seconds. Numbers in parentheses indicate 95% confidence interval from the fit.

Temperature (K)	T_m at $g_{\|\|}$, 1.1 mM	T_m at $g_{\perp}$, 1.1 mM	T_m at $g_{\|\|}$, 11 mM	T_m at $g_{\perp}$, 11 mM
5	2.31 (10) E-06	2.11 (7) E-06	2.03 (8) E-06	8.92 (12) E-07
10	2.10 (5) E-06	1.91 (4) E-06	1.80 (4) E-06	8.73 (8) E-07
15	1.93 (4) E-06	1.76 (3) E-06	1.71 (3) E-06	8.31 (7) E-07
20	1.75 (3) E-06	1.58 (2) E-06	1.52 (3) E-06	7.74 (6) E-07
30	1.31 (2) E-06	1.198 (11) E-06	1.061 (14) E-06	6.23 (5) E-07
40	8.23 (13) E-07	8.01 (6) E-07	5.69 (6) E-07	4.43 (3) E-07
50	4.97 (13) E-07	5.25 (5) E-07	2.95 (5) E-07	2.99 (3) E-07
70	2.4 (3) E-07	2.78 (5) E-07	2.1 (2) E-07	1.90 (5) E-07

Table S7: Tabulated T_m (Hahn echo decay) data for **1** in PMMA thin film. All data in seconds. Numbers in parentheses indicate 95% confidence interval from the fit.

Temperature (K)	T_m at $g_{\|\|}$	T_m at $g_{\perp}$
5	2.84 (4) E-07	1.89 (2) E-07
10	2.75 (4) E-07	1.85 (2) E-07
20	2.48 (4) E-07	1.75 (2) E-07
40	1.57 (3) E-07	1.32 (2) E-07
50	1.08 (3) E-07	1.01 (2) E-07

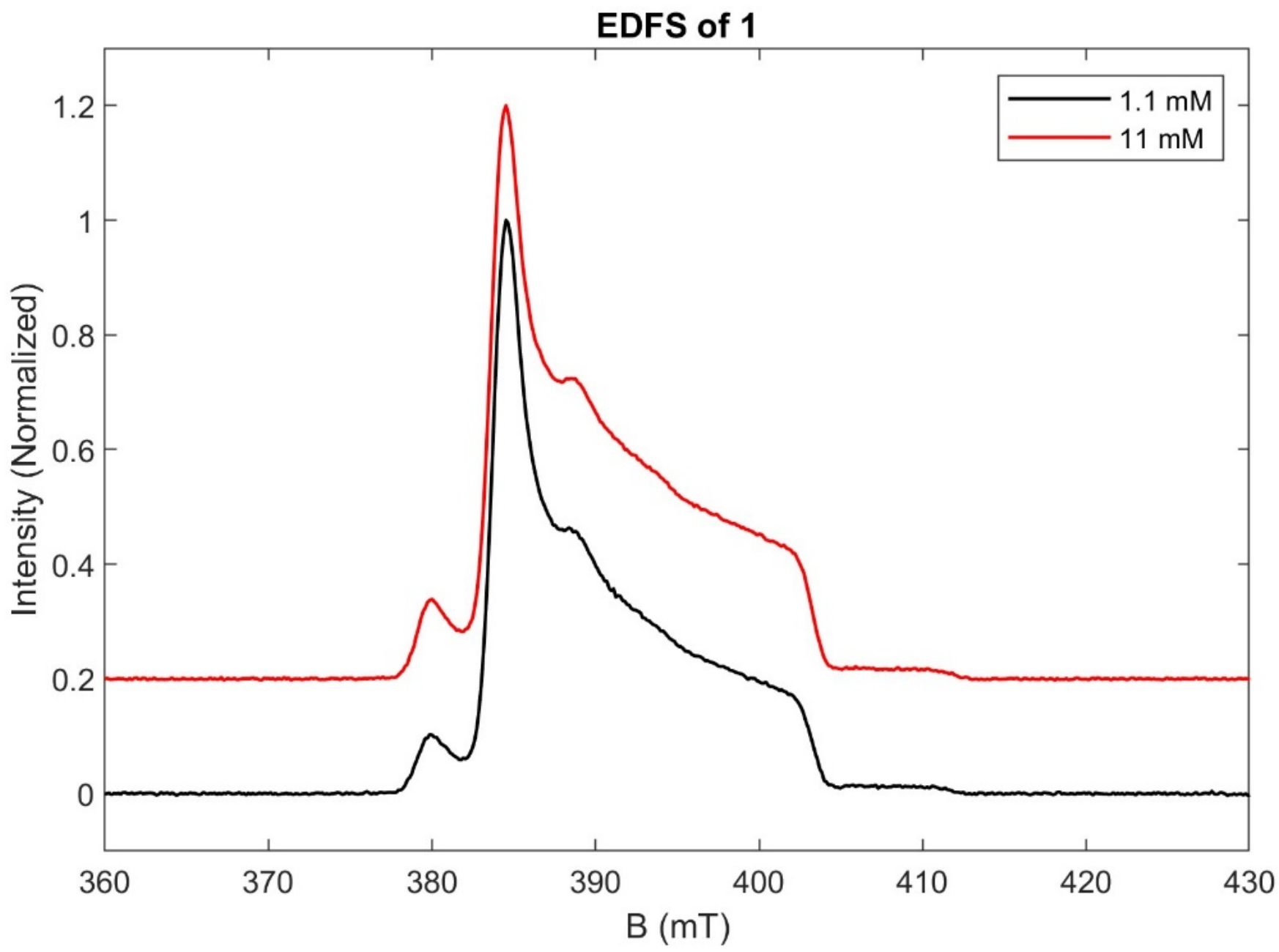

Figure S36: X-band (9.69 GHz) echo-detected field swept spectra of **1** in 2-MeTHF at 1.1 mM (black) and 11 mM (red). Collected at 5 K.

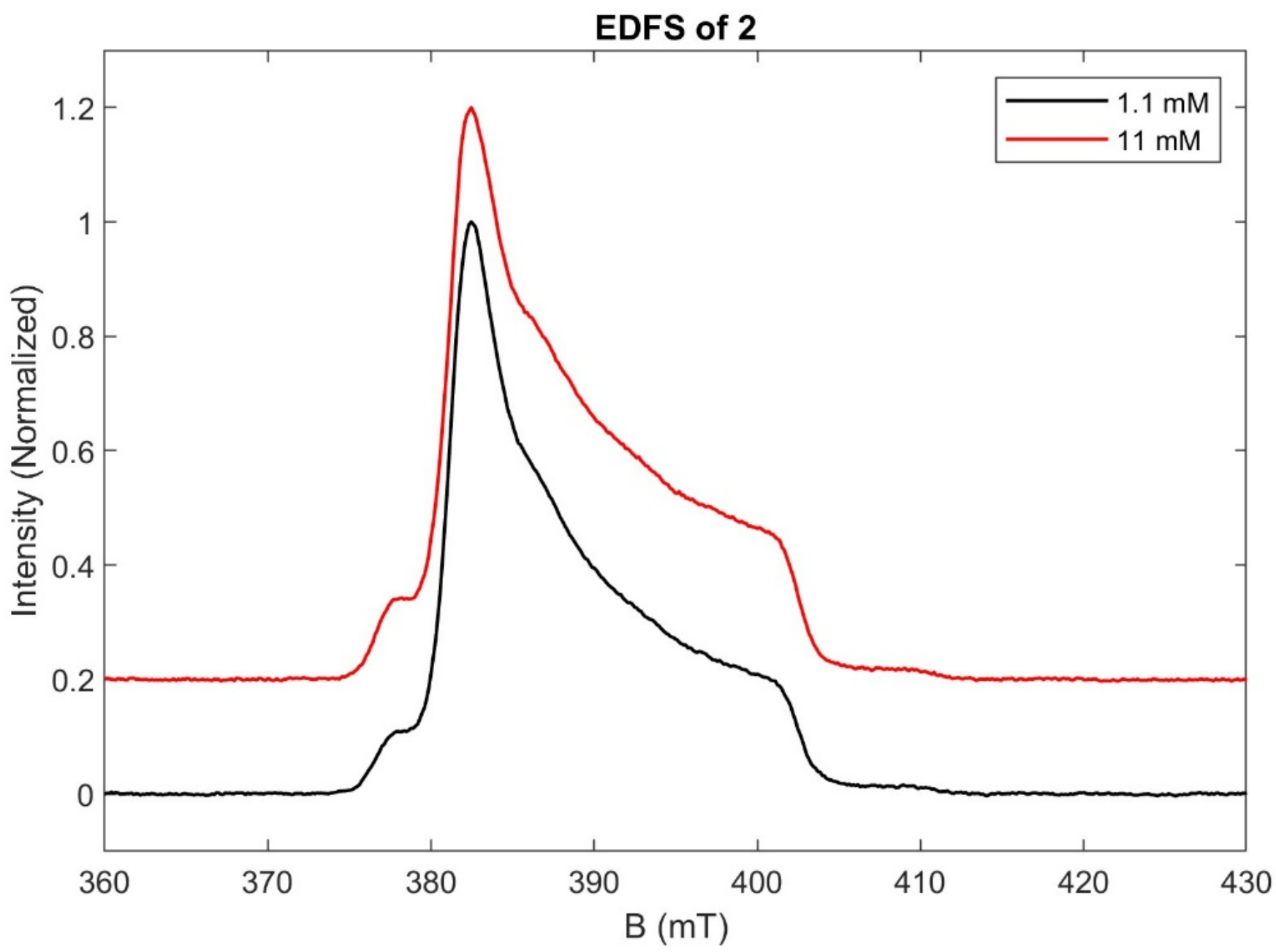

Figure S37: X-band (9.69 GHz) echo-detected field swept spectra of **2** in 2-MeTHF at 1.1 mM (black) and 11 mM (red). Collected at 5 K.

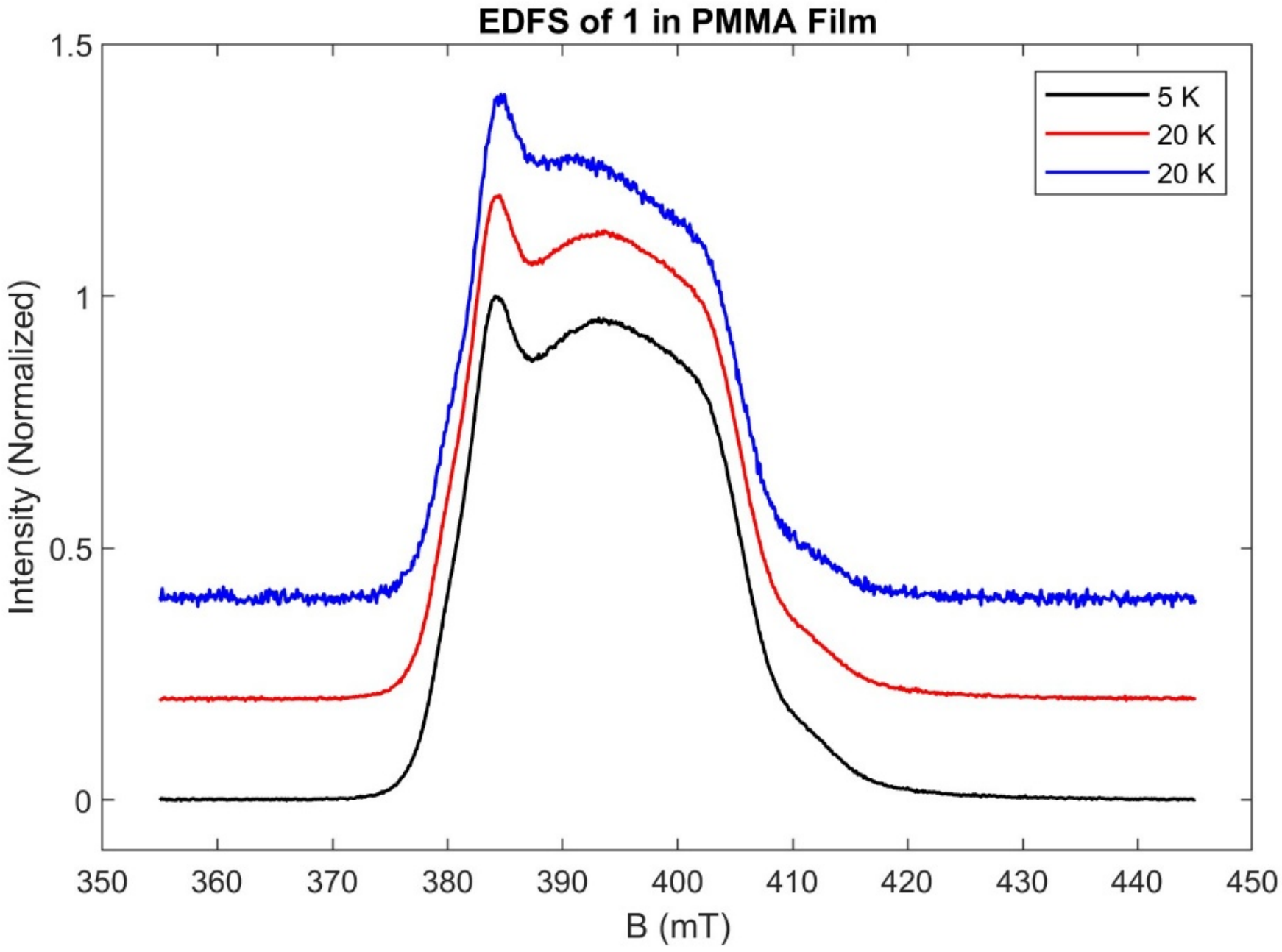

Figure S38: X-band (9.69 GHz) echo-detected field swept spectra of **1** in PMMA thin film at 5 K (black), 20 K (red), and 50 K (blue). Suppression of intensity near $g_\perp$ (i.e. near 384 mT) relative to 2-MeTHF samples is consistent with a high concentration of spins in the film.

S4. Time Resolved Faraday Spectroscopy (TRFE/R)

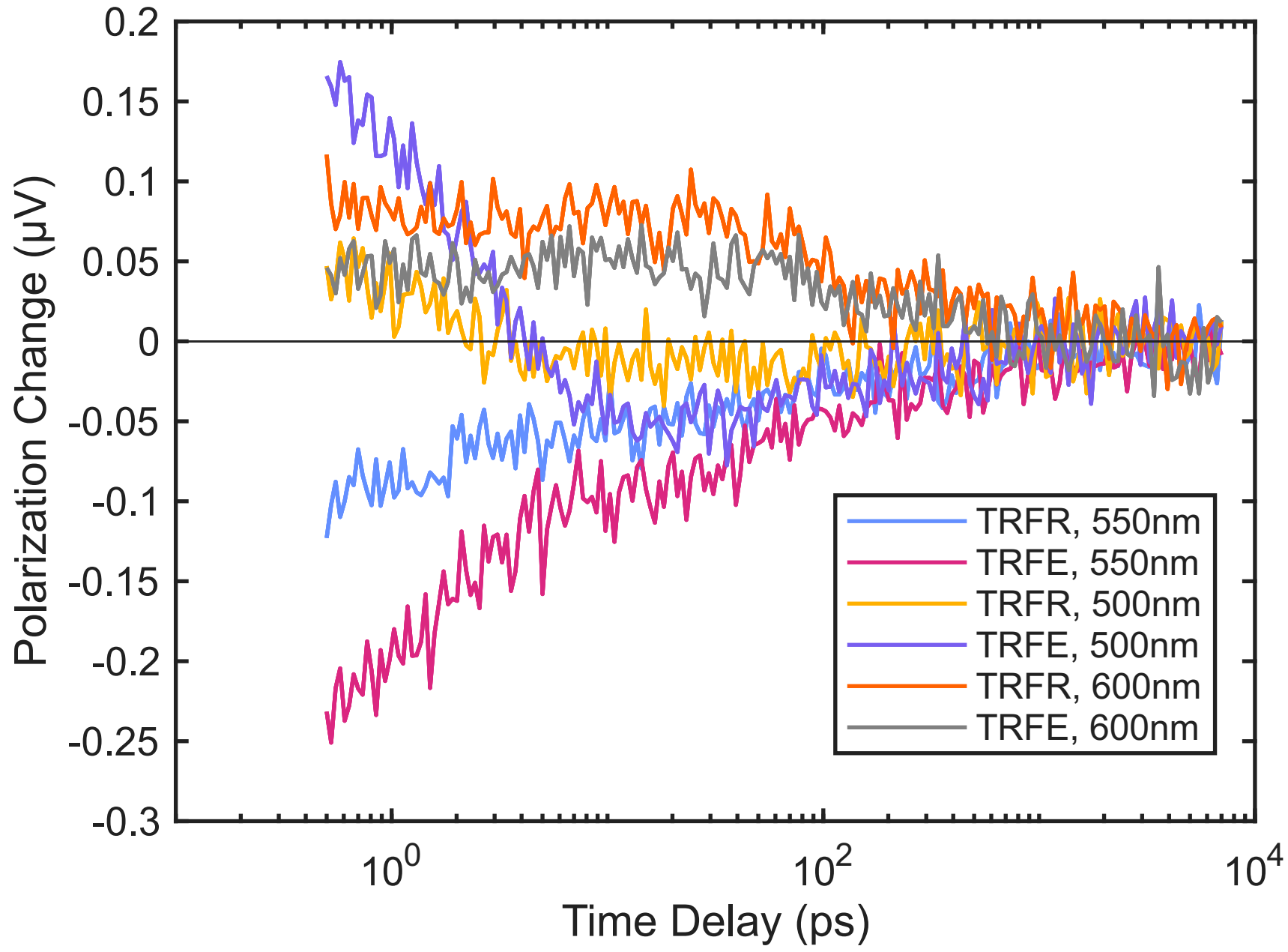

Figure S39. TRFE/R of **1** at various probe wavelengths. All appear to show the same three time constants, but the shortest flips relative to the other two at some wavelengths.

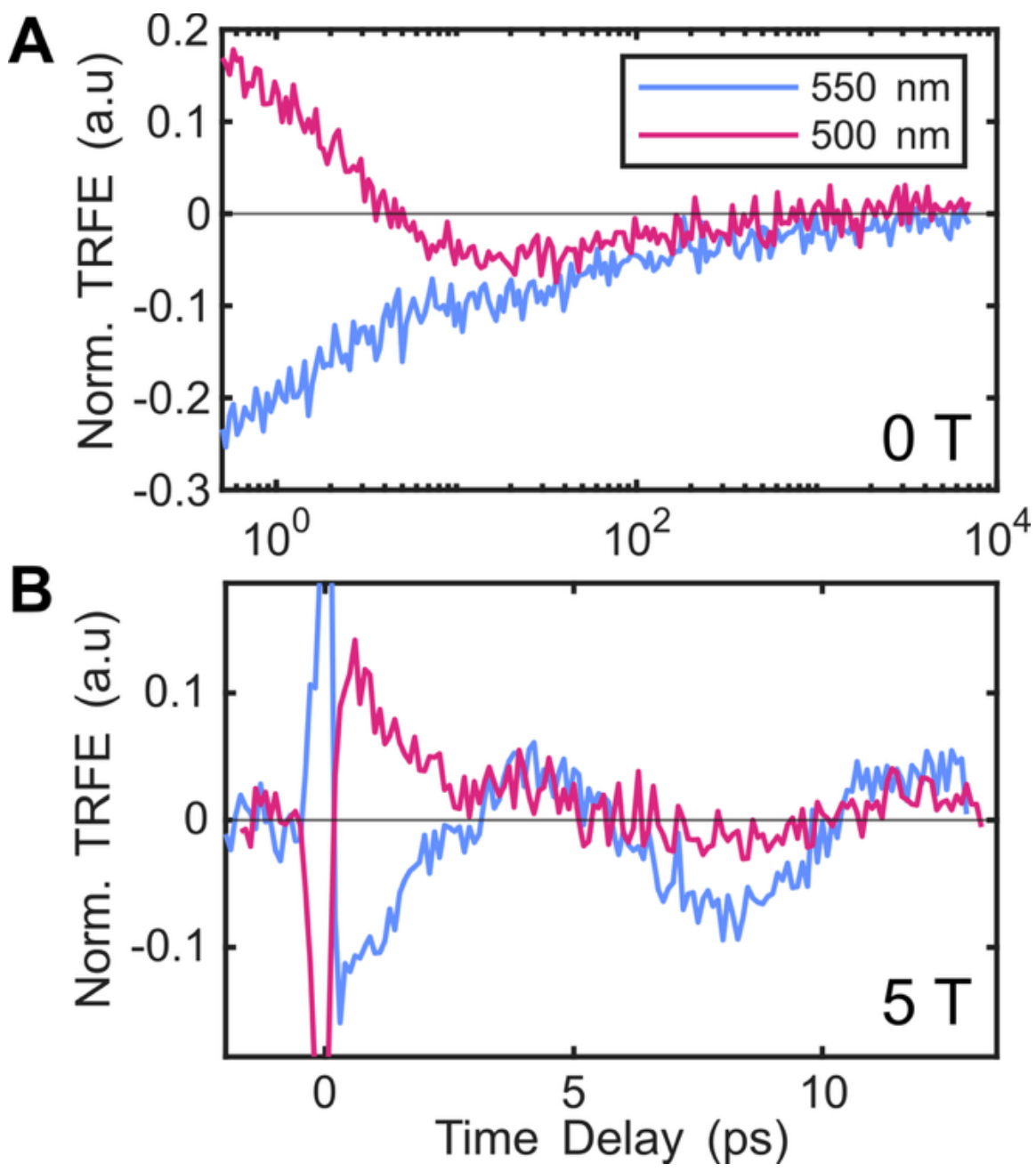

Figure S40. TRFE of **1** at two different probe wavelengths highlighting the behavior of the artefact in the first 10 ps under (**A**) 0 T and (**B**) 5 T applied magnetic fields. Oscillations are visible at 5T for both wavelengths, where the artefact leaves a small offset in the data.

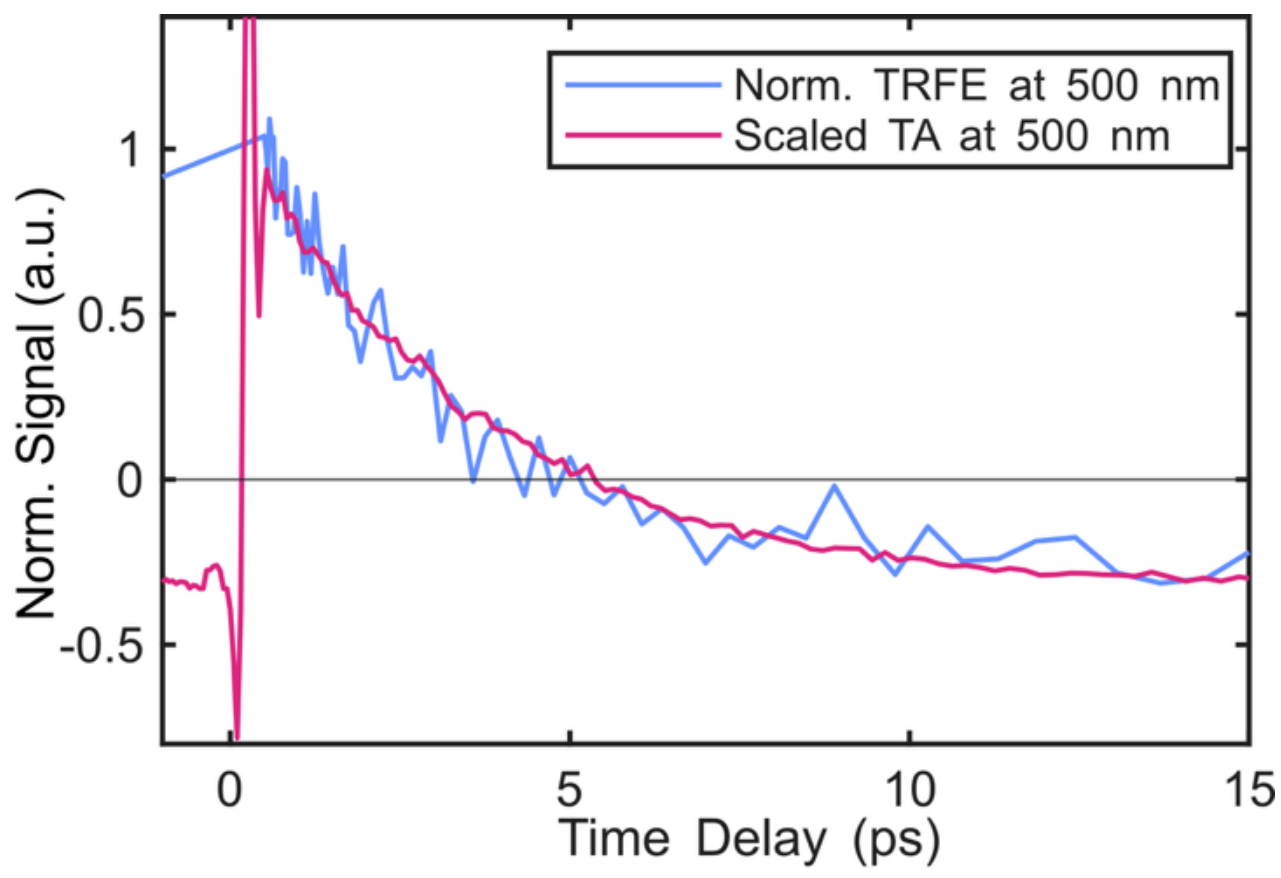

Figure S41. TRFE and TA of **1** at 500 nm probe. Both spectra are scaled such that they overlap to highlight the similar timescale of decay. TRFE data are the same as **Figure S39** and the TA is described in full in **Section S5**.

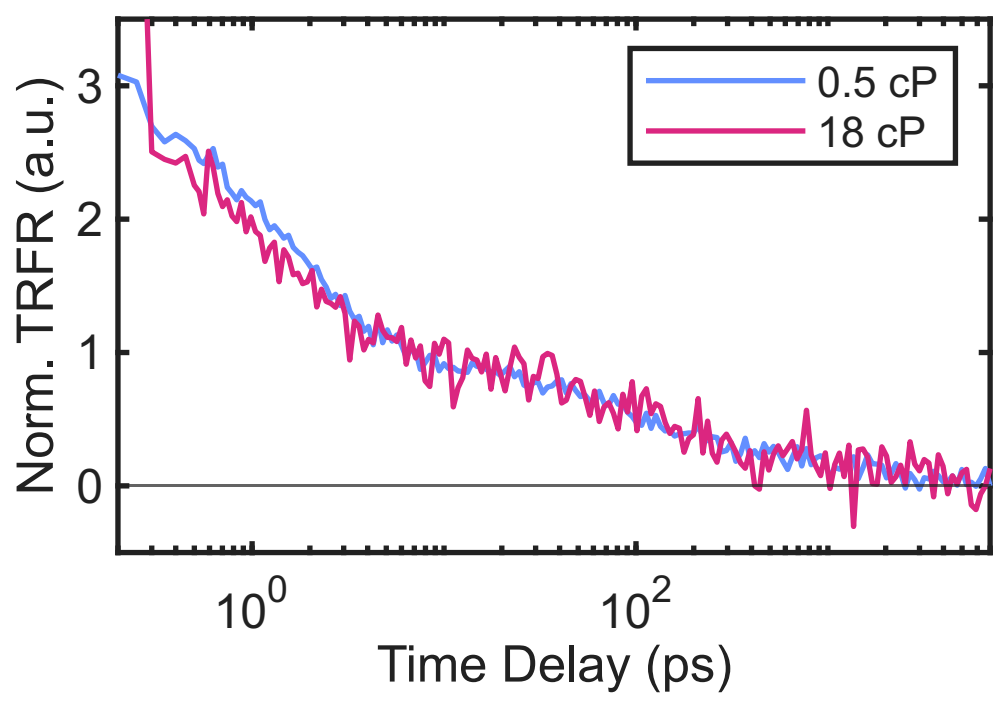

Figure S42. TRFR of **1** in neat THF (blue) and in THF with added polystyrene (pink).

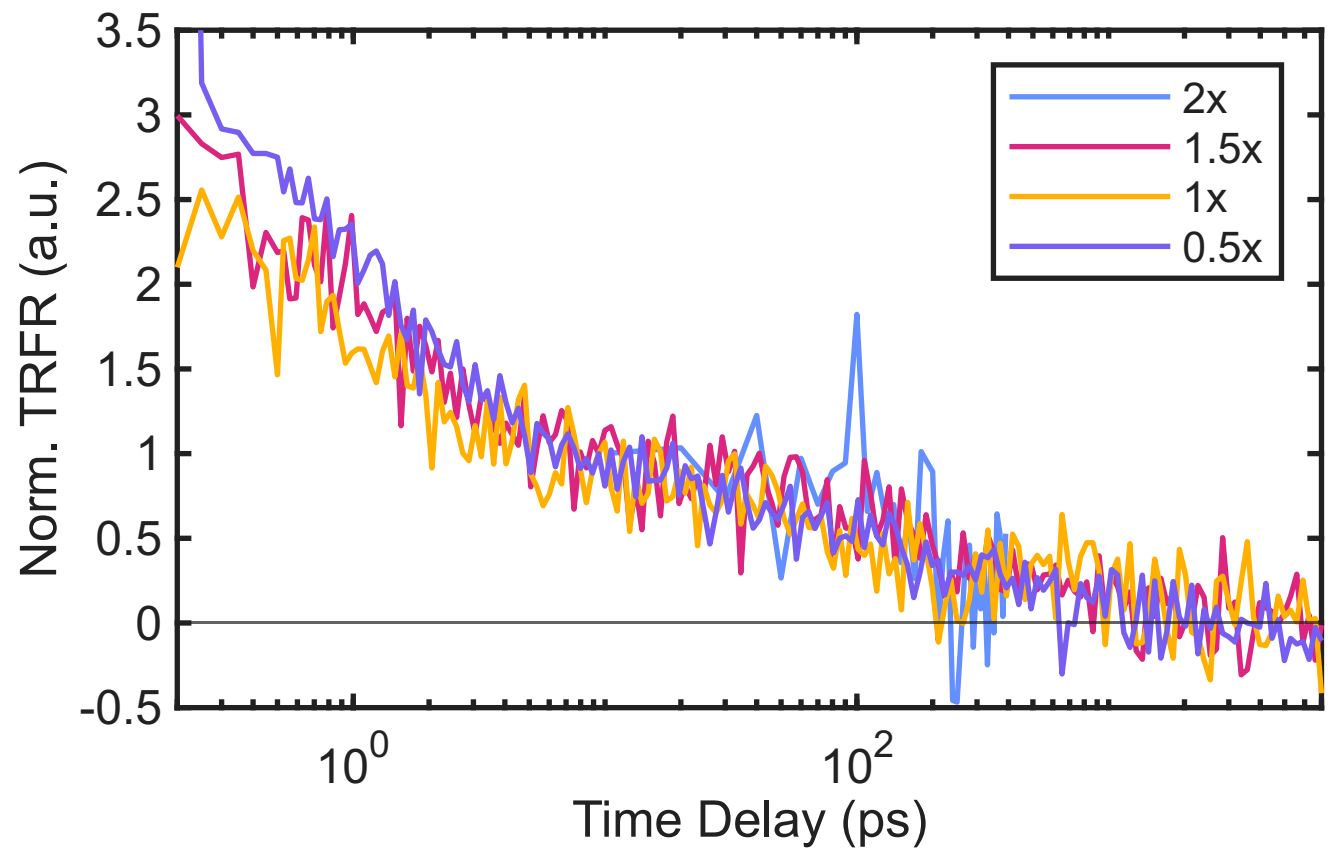

Figure S43. TRFR of **1** in THF for a range of concentrations. The legend lists the concentration relative to 11 mM; in ascending order these are roughly: 5.5, 11, 16.5, and 22 mM.

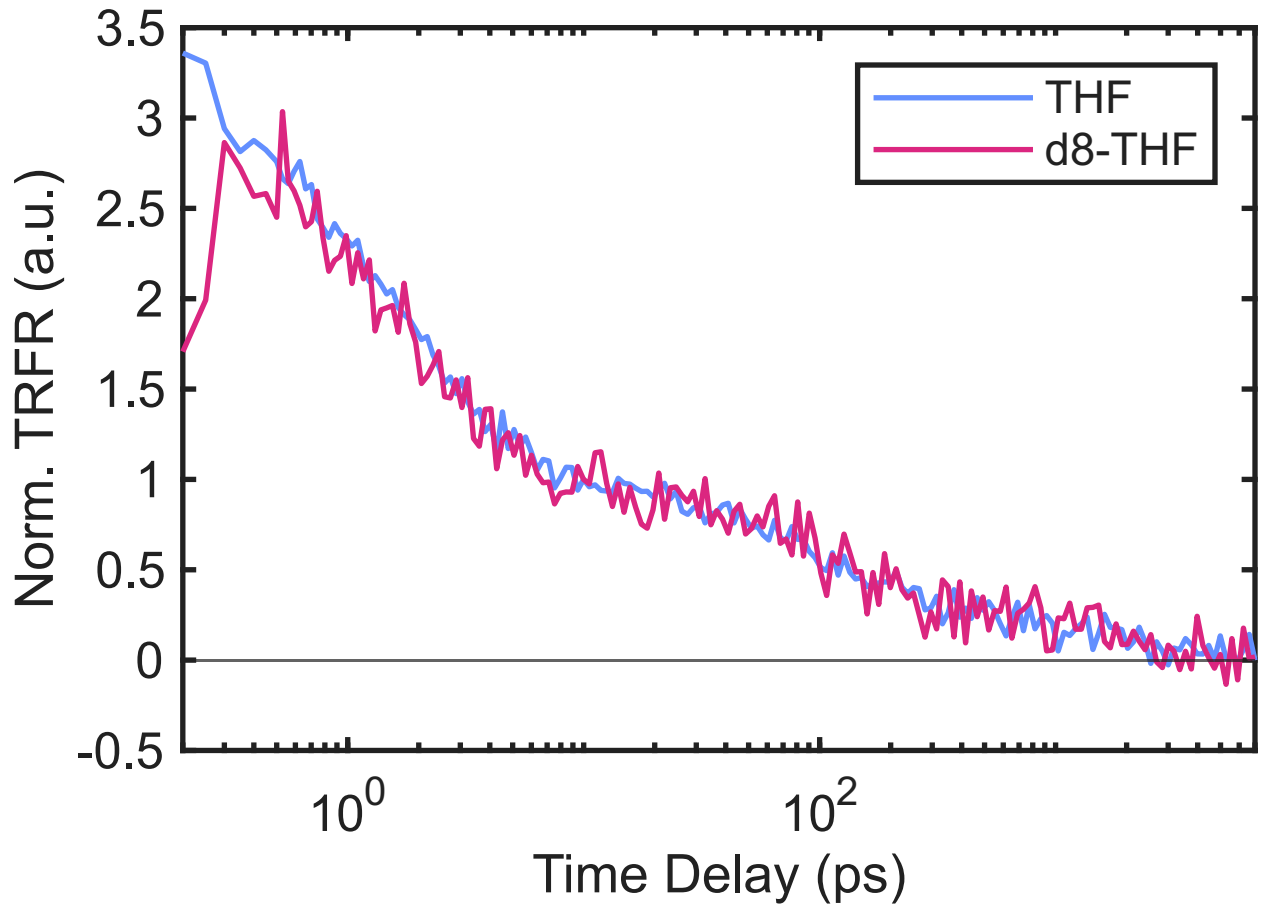

Figure S44. TRFR of **1** in THF (blue) and deuterated THF (pink).

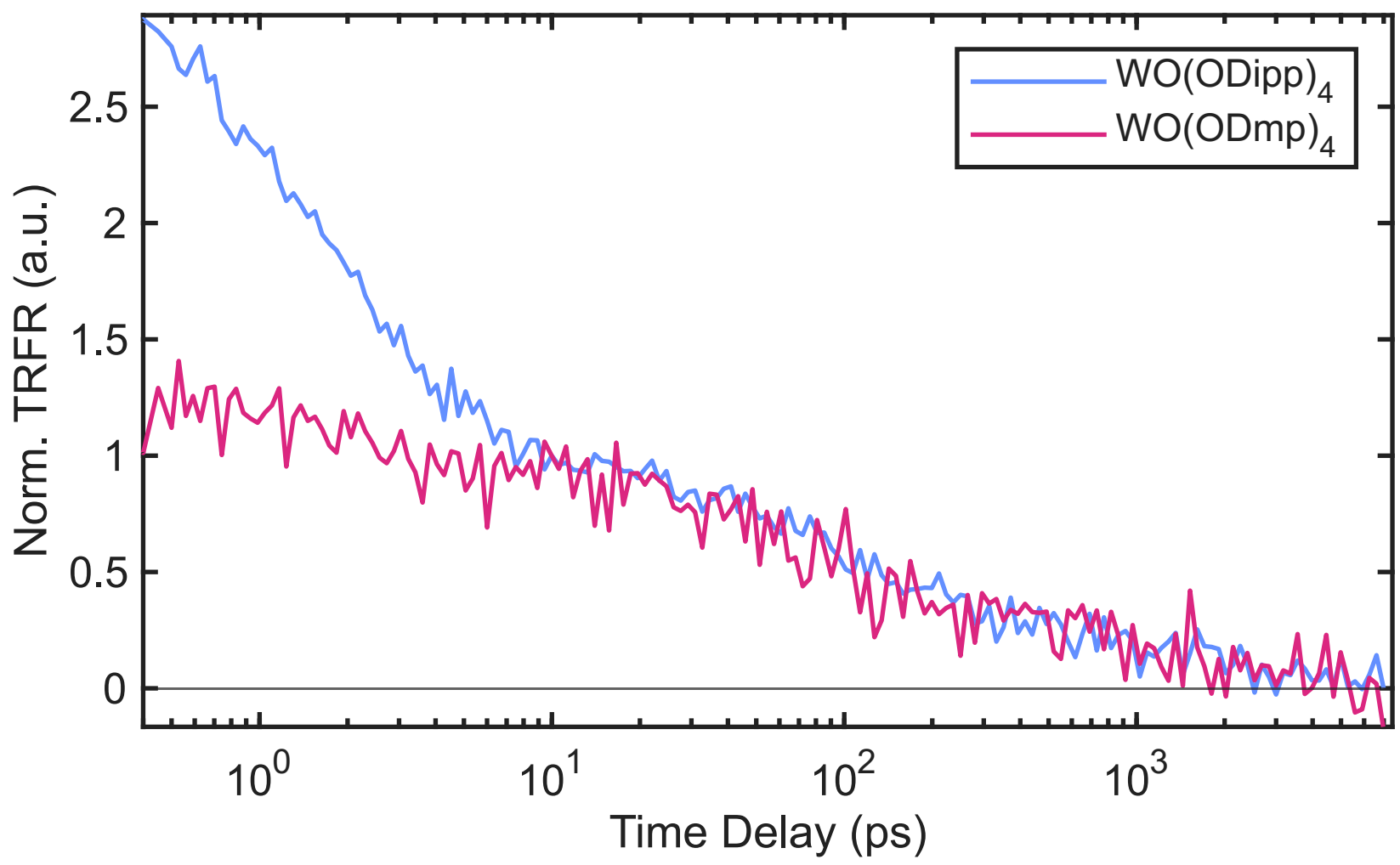

Figure S45. TRFR of **1** (blue) and **2** (pink) in THF.

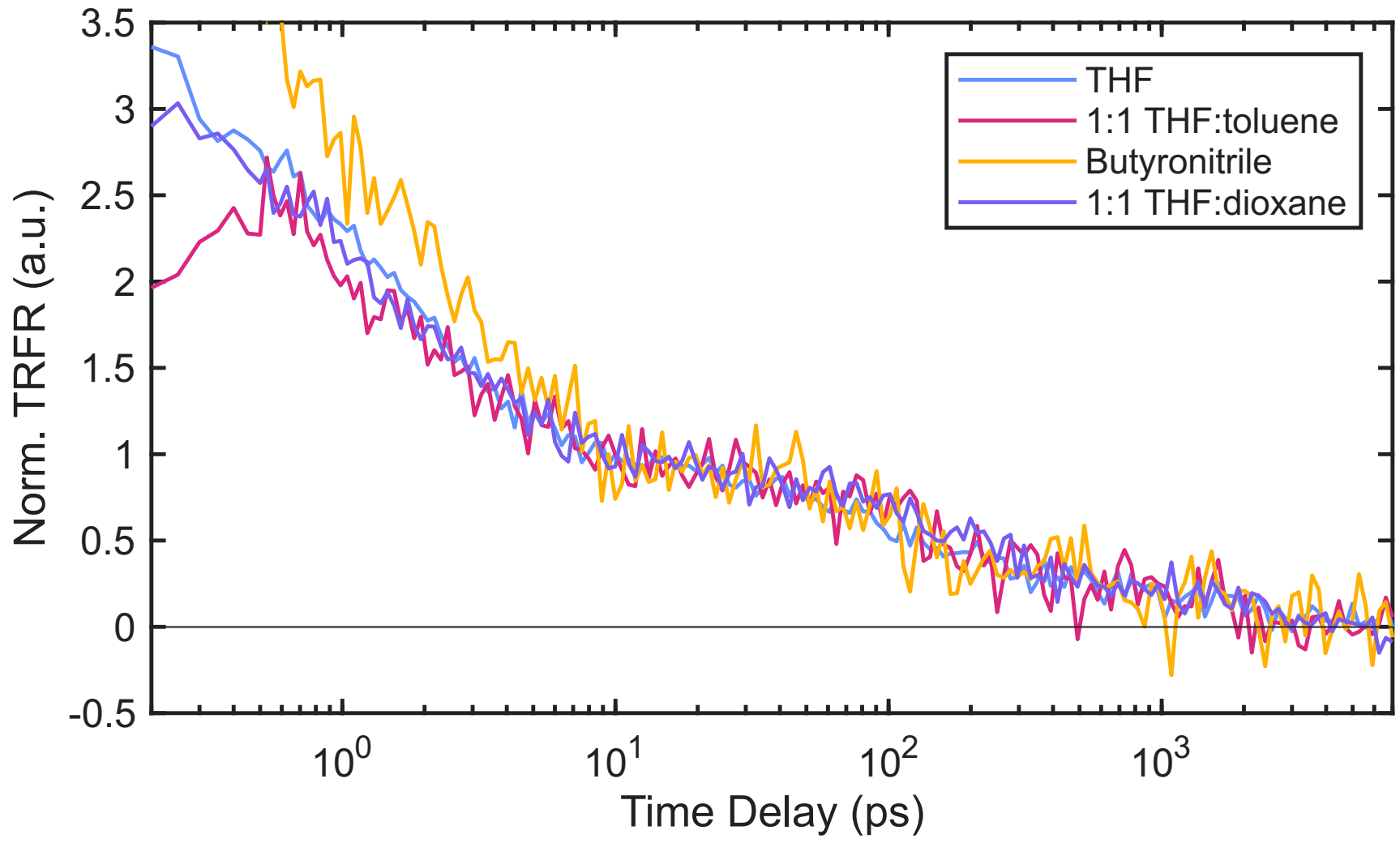

Figure S46. TRFR of **1** in various solvents, all at 11 mM.

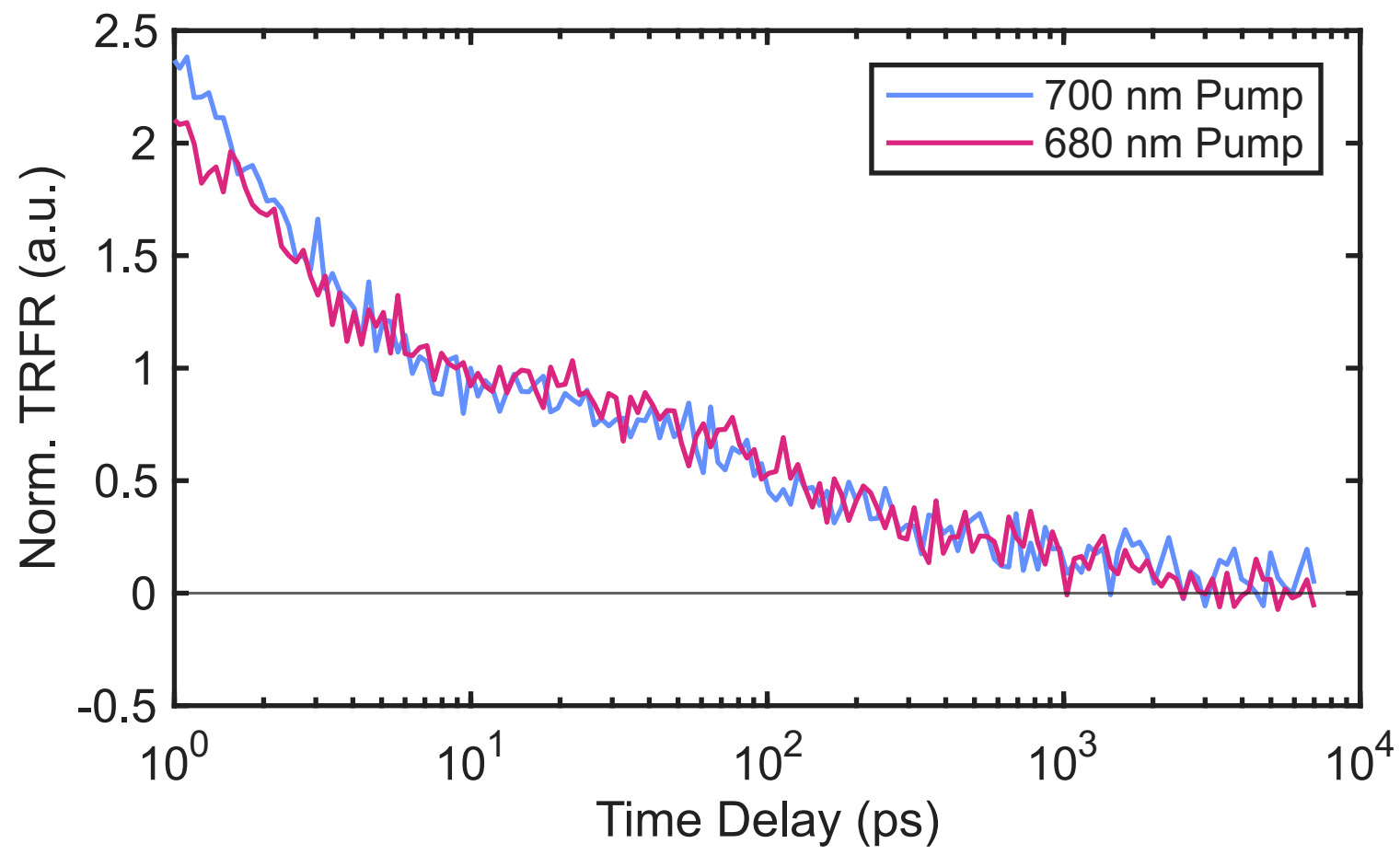

Figure S47. TRFR of **1** following photoexcitation at 700 and 680 nm, both overlap exactly.

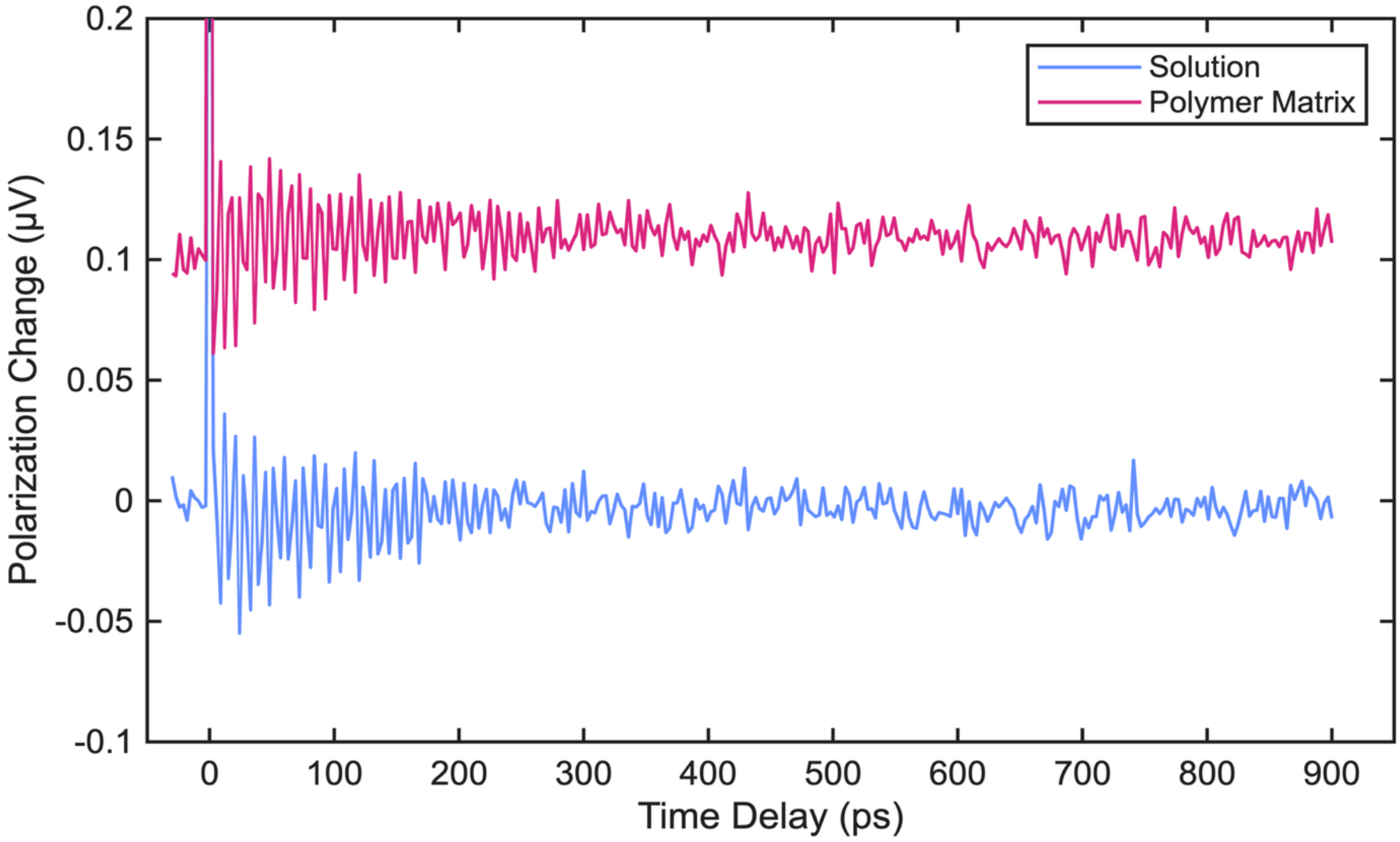

Figure S48. TRFR and TRFE of **1** at 5 T in solution and polymer matrix, respectively.

Table S8: Parameters used with the EasySpin pepper function to simulate 5 T spectrum observed for the film with TRFE. First four parameters are taken from the CW solution simulations (**Table S1**). Final three are broadening parameters used to approximate the observed linewidth.

$g_{\|\|}$	$g_{\perp}$	$A_{\|}$	$A_{\perp}$	lw	$g_{strain,\|\|}$	$g_{strain,\perp}$
1.801	1.715	228	415	[100 100]	0.013	0.01

S5. Transient Absorption (TA) Spectroscopy.

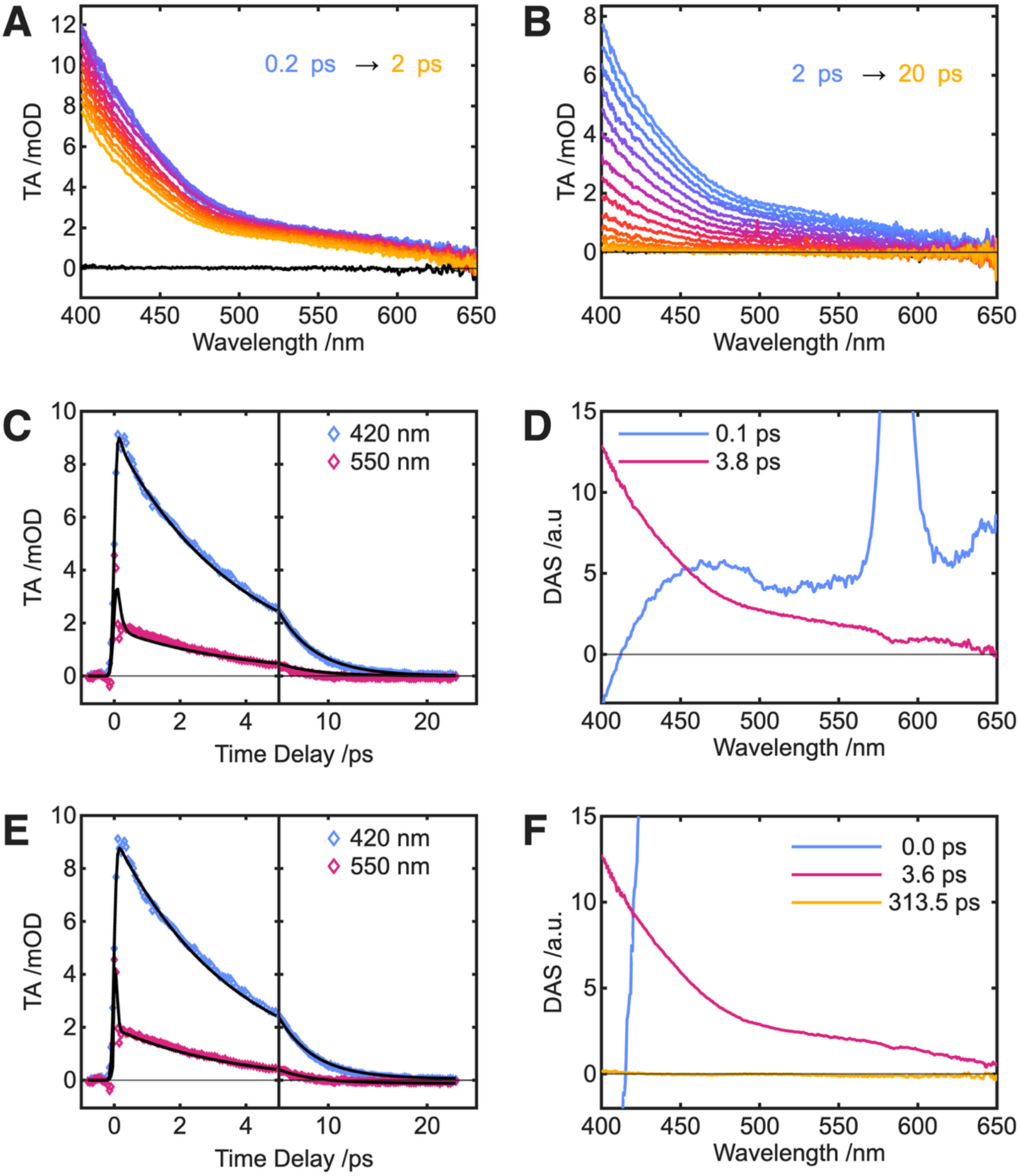

Figure S49. TA of **1** in THF following a 680 nm, 1.1 μJ pump pulse. (**A**,**B**) Difference spectra for shorter and longer time regions. Multiexponential global fits to the data with (**C**,**D**) 2 and (**E**,**F**) 3 exponentials are presented. (**C**,**E**) Fits plotted alongside kinetic traces at two wavelengths. (**D**,**F**) Decay associated spectra (DAS) for the fits with corresponding time constants denoted in the legend. Best fit achieved with three components: <0.1 ps, 3.6 ps, >100 ps. The shortest time constant fits only the cross-phase modulation artefact at time-zero so can be ignored. The longest appears to be a scattering artefact given the absence of ground state bleach. The excited state electronic lifetime (likely relaxing from the lowest doublet d-d excited state) is therefore taken as 3.6 ps, much shorter than T_2^*.

S6. Discussion of Decoherence Mechanisms

True biexponential kinetics arise from two decay processes, which may be sequential (ES2→ES1→GS) or parallel (ES2→GS, ES1→GS). Only the latter can apply here since $S = 1/2$ spins only have two available states in the absence of multiple excited spin states (neglecting hyperfine, and other higher order effects). A parallel decay would arise from decoherence across two independent species with different T_2^*. Here we discuss three potential origins for this signal: different tungsten isotopes, T_1 anisotropy, and anisotropic absorption of light.

Of stable tungsten isotopes, only ^{183}W has non-zero nuclear spin and constitutes 14% of naturally occurring tungsten atoms. This would change the hyperfine coupling to the electron spin between ^{183}W-centered complexes and those of other isotopes, which could potentially lead to a difference in decoherence rate. However, the order of magnitude difference observed is more than one would expect from hyperfine coupling, especially within the spin diffusion barrier. More importantly, since the 100 ps decay component disappears upon immobilization, its origin must be linked to the physical orientation of the molecule.

Anisotropy in the decoherence constitutes one potential cause linked to orientation and large differences in T_1 between $g_\parallel$ and $g_\perp$ have indeed been observed previously in $S = 1/2$ transition metal complexes. Isotropically oriented molecules would result in some spins excited along $g_\parallel$ and some along $g_\perp$ (and all orientations in between) which could result in two spin relaxation rates observed simultaneously. The observed difference upon freezing the molecular orientation implies that there is initially an anisotropic distribution which then tends to isotropic as the molecules tumble in solution. If the molecules are not free to rotate then the initial distribution remains the same. However, to generate an initial anisotropic distribution of spins in the randomly oriented molecules, light must be absorbed preferentially along one molecular axis. This is expected to be the case here, given the lower symmetry of the tungsten complexes. At the same time, this axial spin-photon coupling should also reduce the signal measured by the probe as the photoexcited molecules rotate away from their initial distribution, as described in the main text. We cannot rule this T_1 anisotropy mechanism out, but it can only occur if there is also strongly anisotropic electronic absorption, which itself could also give rise to a biexponential decay.

The simplest explanation that describes the observed biexponential decoherence dynamics is therefore that of anisotropic absorption of light. This of course will require further investigation to definitively prove or disprove. However, for the reasons above, we can think of no other explanation as of yet that can satisfy all observations.

Contributions to decoherence from methyl rotation also merit discussion. At cryogenic temperatures, T_m in $S = 1/2$ organic radicals[1] and transition metal complexes[2] is suppressed in the presence of methyl-containing solvents, with less hindered methyl groups (i.e. lower rotation barriers) conferring faster dephasing. At higher temperatures, however, contributions from spin-lattice relaxation and molecular tumbling become dominant, as shown for VO^{2+} and Cr(V) complexes.[2] In the case of the tungsten(V) complexes explored herein, it is plausible that methyl rotation is a major driver of decoherence at cryogenic temperatures, especially because all pulse EPR data was collected in a methyl-containing solvent (2-methyltetrahydrofuran). Changes in the quantity of methyl groups on the phenoxide ligands and their rotation barriers may also contribute to the slight differences observed in T_m between **1** and **2** below 40 K, particularly at $g_\parallel$ (**Figure S14**). It appears unlikely, however, that methyl rotation is a primary contributor to decoherence at room temperature, for several reasons: No changes in TRFR spin dynamics were observed between non-methylated (THF and 1:1 THF:dioxane) and methyl-containing (butyronitrile and 1:1 THF:toluene) solvents (**Figure S46**), nor were any changes

observed between **1** and **2** in THF (**Figure S45**). Additionally, the increased temperature dependence of T_m and near-convergence with T_1 (**Figures S10** and **S11**) between 40 and 70 K indicates entry into a T_1-limited regime well below room temperature. Finally, despite the high density of methyl groups in poly(methyl methacrylate), immobilization of **1** in a PMMA film does not (within noise) appear to alter the upper limit of coherence (~2 ns) (**Figure 4A**).

S7. X-Ray Crystallography

Low-temperature diffraction data (ϕ-and ω-scans) were collected on a Bruker AXS D8 VENTURE KAPPA diffractometer coupled to a PHOTON II CPAD detector with Mo K_α radiation (λ = 0.71073 Å) from an IμS micro-source for the structure of compound V26039. The structure was solved by direct methods using SHELXS[3] and refined against F[4] on all data by full-matrix least squares with SHELXL-2019[4] using established refinement techniques.[5] All non-hydrogen atoms were refined anisotropically. All hydrogen atoms were included into the model at geometrically calculated positions and refined using a riding model. The isotropic displacement parameters of all hydrogen atoms were fixed to 1.2 times the *U* value of the atoms they are linked to (1.5 times for methyl groups). All disordered atoms were refined with the help of similarity restraints on the 1,2- and 1,3-distances and displacement parameters as well as enhanced rigid bond restraints for anisotropic displacement parameters.

Compound V26039 (CCDC 2550952) crystallizes in the tetragonal space group *P4/ncc* with a quarter of a molecule in the asymmetric unit. The highest electron density maximum is located on the 4-fold rotation axis in a chemically unreasonable location and was not refined.

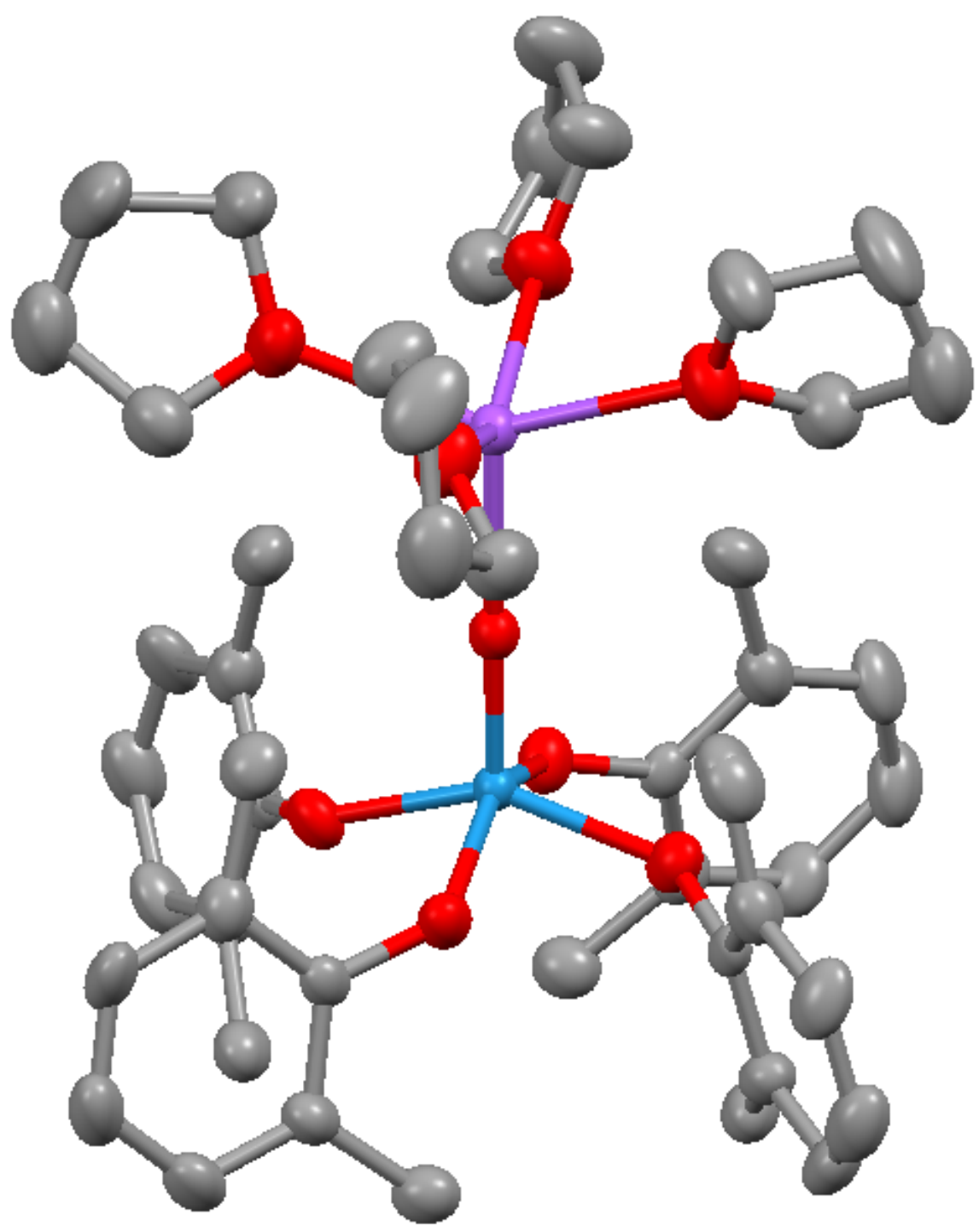

Figure S50: Crystal structure of **2**. Dimethylphenolate ligands are each disordered over two positions, but for clarity, only one position is shown above. Hydrogen atoms are also omitted for clarity.

Table S9. Crystal data and structure refinement for V26039 (CCDC 2550952)

Identification code	V26039
Empirical formula	C12 H17 Na0.25 O2.25 W0.25
Formula weight	248.96
Temperature	100(2) K
Wavelength	0.71073 Å
Crystal system	Tetragonal
Space group	P4/ncc
Unit cell dimensions	a = 13.4381(4) Å a= 90°.
	b = 13.4381(4) Å b= 90°.
	c = 25.9450(13) Å g = 90°.
Volume	4685.2(4) $Å^3$
Z	16
Density (calculated)	1.412 Mg/m^3
Absorption coefficient	2.527 mm^{-1}
F(000)	2052
Crystal size	0.100 x 0.100 x 0.100 mm^3
Theta range for data collection	2.657 to 25.027°.
Index ranges	-15<=h<=15, -15<=k<=15, -30<=l<=30
Reflections collected	98370
Independent reflections	2083 [R(int) = 0.0330]
Completeness to theta = 25.027°	99.9 %
Absorption correction	Semi-empirical from equivalents
Max. and min. transmission	0.0998 and 0.0680
Refinement method	Full-matrix least-squares on F^2
Data / restraints / parameters	2083 / 507 / 222
Goodness-of-fit on F^2	1.214
Final R indices [I>2sigma(I)]	R1 = 0.0364, wR2 = 0.0705
R indices (all data)	R1 = 0.0511, wR2 = 0.0819
Extinction coefficient	n/a
Largest diff. peak and hole	2.423 and -2.045 e.$Å^{-3}$

Note Regarding Check-Cif Alert:

PLAT971_ALERT_2_B Check Calc'd Residual Density 2.70 Å from C8A (3.08 eA^{-3})

Author Response: This residual density is not located in a chemically reasonable location. It is located on a 4-fold rotation axis and appears to be an artifact caused by the rotation axis.

S8. References.

1. Zecevic, A.; Eaton, G. R.; Eaton, S. S.; and Lindgren, M. Dephasing of electron spin echoes for nitroxyl radicals in glassy solvents by non-methyl and methyl protons. *Molecular Physics*, **95**, 1255-1263 (1998)
2. Eaton, G. R. and Eaton, S. S. Solvent and temperature dependence of spin echo dephasing for chromium(V) and vanadyl complexes in glassy solution. *Journal of Magnetic Resonance*, **136**, 63-68, (1999)
3. Sheldrick, G. M. Phase annealing in SHELX-90: direct methods for larger structures. *Acta Cryst A* **46**, 467-473 (1990).
4. Sheldrick, G. M. Crystal structure refinement with SHELXL. *Acta Cryst C* **71**, 3-8 (2015).
5. Müller, P. Practical suggestions for better crystal structures. *Crystallography Reviews* **15**, 57-83 (2009)
